# The Zinc Finger Protein ZNF658 Regulates the Transcription of Genes Involved in Zinc Homeostasis and Affects Ribosome Biogenesis through the Zinc Transcriptional Regulatory Element

**DOI:** 10.1128/MCB.01298-14

**Published:** 2015-02-18

**Authors:** Ogo A. Ogo, John Tyson, Simon J. Cockell, Alison Howard, Ruth A. Valentine, Dianne Ford

**Affiliations:** a Institute for Cell and Molecular Biosciences, Newcastle University, Newcastle upon Tyne, United Kingdom; b School of Dental Sciences, Newcastle University, Newcastle upon Tyne, United Kingdom; c Human Nutrition Research Centre, Newcastle University, Newcastle upon Tyne, United Kingdom; d Faculty of Medical Sciences, Newcastle University, Newcastle upon Tyne, United Kingdom

## Abstract

We previously identified the ZTRE (zinc transcriptional regulatory element) in genes involved in zinc homeostasis and showed that it mediates transcriptional repression in response to zinc. We now report that ZNF658 acts at the ZTRE. ZNF658 was identified by matrix-assisted laser desorption ionization–time of flight mass spectrometry of a band excised after electrophoretic mobility shift assay using a ZTRE probe. The protein contains a KRAB domain and 21 zinc fingers. It has similarity with ZAP1 from Saccharomyces cerevisiae, which regulates the response to zinc restriction, including a conserved DNA binding region we show to be functional also in ZNF658. Small interfering RNA (siRNA) targeted to ZNF658 abrogated the zinc-induced, ZTRE-dependent reduction in *SLC30A5* (ZnT5 gene), *SLC30A10* (ZnT10 gene), and *CBWD* transcripts in human Caco-2 cells and the ability of zinc to repress reporter gene expression from corresponding promoter-reporter constructs. Microarray analysis of the effect of reducing ZNF658 expression by siRNA uncovered a large decrease in rRNA. We find that ZTREs are clustered within the 45S rRNA precursor. We also saw effects on expression of multiple ribosomal proteins. ZNF658 thus links zinc homeostasis with ribosome biogenesis, the most active transcriptional, and hence zinc-demanding, process in the cell. ZNF658 is thus a novel transcriptional regulator that plays a fundamental role in the orchestrated cellular response to zinc availability.

## INTRODUCTION

Coordinated regulation of suites of genes in response to fluctuations in zinc availability is a cornerstone of cellular zinc homeostasis, which is essential across the phyla to allow adaptation to different environmental or nutritional conditions. For example, zinc must be tightly regulated for the correct population of protein metal binding sites and for other functions such as modulation of neurotransmission and intracellular signaling. These zinc-regulated genes include cell membrane zinc transporters and intracellular zinc storage proteins such as metallothionein.

Many transcription factors that control the expression of these genes have been identified and probed mechanistically at the molecular level to develop an understanding of how cells control the availability of zinc under conditions of a variable supply. The main players in bacteria fall into two groups. The ArsR and MerR families are transcriptional derepressors, where zinc binding induces a conformational change to inhibit DNA binding and allow expression of proteins that confer zinc tolerance. Transcription factors in the Fur family (specifically Zur) bind to DNA in the metal-bound form to repress transcription of zinc resistance genes (reviewed in reference 1). In the budding yeast Saccharomyces cerevisiae the transcription factor ZAP1 coordinates the expression of genes involved in zinc homeostasis generally, although not exclusively (2, 3), through a transcriptional activating function when zinc-limiting conditions promote binding to DNA. ZAP1 targets include genes coding for transporters that increase cytosolic zinc concentration under these conditions, notably the high-affinity zinc uptake transporter ZRT1 (4) and the vacuolar zinc exporter ZRT3 (5). In the fission yeast Saccharomyces pombe the transcription factor Loz1 plays a role in zinc homeostasis through responding to zinc excess to repress a suite of genes including those coding for the high-affinity zinc uptake transporter Zrt1 and for Adh4, a putative mitochondrial iron-dependent alcohol dehydrogenase that is expressed preferentially to the abundant, zinc-dependent Adh1 when zinc is limiting (6).

In mammals the transcription factor MTF1 responds to zinc to translocate from the cytoplasm to the nucleus and to bind to the metal response element (MRE). Binding of zinc to the outermost zinc fingers (1, 5, and 6) of the N-terminal DNA-binding domain, which comprises six Cys_2_His_2_ zinc fingers, appears to be responsible for the zinc-sensing function (7). Acidic, proline-rich, and serine/threonine-rich domains in the carboxyl half of the protein together form the transcription activation domain (8). The classical MTF1-induced response is activation of transcription, including transcription of the *SLC30A1* (ZnT1) gene (9), whose product mediates efflux of zinc from the cell (10), and of metallothionein genes (11). However, MTF1 can also repress gene transcription, as observed for *Slc39a10* (ZIP10) genes in both zebrafish (12) and mice (13), and for human and mouse selenoprotein H genes (14). Kruppel-like factor 4 (KLF4) has a role in the inducing transcription of the *Slc39A4* (Zip4) gene in mouse intestine under conditions of restricted zinc supply (15), but potential wider-ranging actions of this transcription factor in zinc homeostasis have not been reported.

Major gaps in knowledge and understanding of transcriptional regulatory processes important in zinc homeostasis remain. Notably, the identity of a metazoan transcriptional regulatory protein with a function to repress transcription of multiple genes to coordinate a homeostatic cellular response under conditions of elevated zinc availability has not been uncovered. Our recent identification of a regulatory DNA sequence element as the binding site for a protein with this function—the ZTRE (zinc transcriptional regulatory element) (16)—advanced our understanding of this process. We report here the identity of a transcription factor that acts through the ZTRE to mediate transcriptional repression in response to zinc of a suite of genes in human cells. We also report that this transcription factor plays a role in the control of ribosome biogenesis, thus coordinating this highly zinc-demanding process with the available zinc supply.

## MATERIALS AND METHODS

### Cell culture, transfection, and treatment with zinc.

Caco-2 cells were grown and passaged under standard conditions as described previously (17) and seeded at 3.5 × 10^5^ cells/9.6-cm^2^ well in six-well plates 24 h prior to transfection with small interfering RNA (siRNA) targeted to ZNF658 (see Table S1 in the supplemental material) or with a control siRNA using Lipofectamine RNAiMAX (Invitrogen), according to the manufacturer's instructions and using 0.35 μg of siRNA per well (10 nM). Medium containing the siRNA transfection mixture was replaced with complete medium after 24 h. To measure the effect of ZNF658 knockdown on the response of genes to zinc by reverse transcription-quantitative PCR (RT-qPCR), medium was replaced with serum-free medium containing either no additional zinc (3 μM) or containing 100 μM ZnSO_4_, and RNA was isolated from cells after a further 24 h. To measure the effect of ZNF658 knockdown on the response of promoter-reporter plasmid constructs to zinc, the cells were then transfected using Lipofectamine 2000 (Invitrogen), according to the manufacturer's instructions, and using 1 μg of plasmid DNA per well, which was added after the cells had been maintained in complete medium for 24 h after ZNF658 knockdown. Medium containing the transfection mixture was replaced with complete medium after 24 h, and after a further 24 h the medium was replaced with serum-free medium either containing no additional zinc (3 μM) or containing 100 μM ZnSO_4_. Protein was extracted from cells for the measurement of reporter gene (β-galactosidase) activity after a further 24 h. The same procedures were used to determine the effect of siRNA targeted to ZNF658 on expression of recombinant ZNF658 (with a C-terminal myc epitope tag), but the order of transfection was reversed such that Caco-2 cells were transfected first with the plasmid construct (pCMV6-ZNF658-myc-DDK) and then with siRNA.

### Plasmid constructs.

Promoter-reporter plasmids (pBlue*SLC30A5*prom, pBlue*SLC30A10*prom, and pBlue*CBWD*prom) were as described previously (16, 18). Plasmid pBlue*SLC30A5*prom_Δ−91to−84_, corresponding to pBlue*SLC30A5*prom but with the 5′ side of the ZTRE (corresponding to bases −91 to −84 relative to the transcriptional start site [TSS] of the *SLC30A5* gene) deleted, was generated from pBlue*SLC30A5*prom by PCR using a pair of complementary primers with sequences that included the ZTRE, omitting the 8 bases to be deleted: _−109_GCGCAGACGCAAGGCTGGG_(CACTCCCC)_CGGGAGTGAGGGTTGCTGGG_−64_ and _−64_CCCCAGCAACCCTCACTCCCG_(GGGGAGTG)_CCCAGCCTTGCGTCTGCGC_−109_. (Sequence numbering is expressed relative to the *SLC30A5* TSS. The full ZTRE sequence spans bases −91 to −76. The subscript text shows the deleted section excluded from the primers. The underlined bases represent the 3′ side of the ZTRE.) The primer concentration was 0.5 μM, and ∼100 ng of template was used. Amplification was performed with HotStarTaq DNA polymerase (Pol; Qiagen), using the supplied buffer with thermal cycling parameters of 95°C for 5 min, followed by 40 cycles of 94°C for 30 s, 57°C for 30 s, and 72°C for 60 s. The (methylated) template was then subjected to digestion using DpnI (Promega), which cleaves at only a methylated target sequence, and then the product, after purification using NucleoSpin gel and PCR Cleanup (Macherey-Nagel), was used to transform Top10 competent Escherichia coli (Invitrogen). Minipreparations of plasmid DNA (QuickLyse miniprep kit; Qiagen) were sequenced (MWG Eurofins) to confirm the presence of the mutation.

Plasmid pCMV6-ZNF658-myc-DDK used for the overexpression of ZNF658 with the myc epitope as a C-terminal fusion, was purchased from Origene. Plasmid pCMV6-ZNF658ΔZF12-15-myc-DDK (for the expression of ZNF658 with zinc fingers 12 to 15 [amino acids 770 to 904] deleted) was generated from pCMV6-ZNF658-myc-DDK by PCR with primers that annealed either side of the required deletion to generate a linear product that was then self-ligated. The primers were _2878_TCAGGGGAGAAGCCCTATGAATGCAGTG_2905_ and _2459_GGGTTTCTCCCCTGTGTGAATTCTCCGATG_2430_ (numbering according to ZNF658 RefSeq NM_033160.5) and used at 1 μM with ∼100 ng of template. Amplification was performed with Phusion high-fidelity DNA polymerase (Thermo Scientific), using the supplied buffer plus 5% (vol/vol) dimethyl sulfoxide under thermal cycling parameters of 98°C for 3 min and then 35 cycles of 98°C for 30 s and 72°C for 4 min and 30 s. Digestion of the template using DpnI and product purification was carried out as described above. The purified product was then ligated using T4 DNA ligase (Promega) and used to transform Top10 competent E. coli (Invitrogen). Minipreparations of plasmid DNA (QuickLyse miniprep kit) were sequenced (MWG Eurofins) to confirm presence of the mutation.

### EMSA and preparation of protein sample for mass spectrometry.

Electrophoretic mobility shift analysis (EMSA) of an infrared dye (IRD)-labeled probe corresponding to a 212-bp region of the ZnT5 (*SLC30A5*) promoter (positions −156 to +46 relative to the transcription start site; GTGGCGGGAGGAGCCTAAGGGACGAGGAAAGGCGAGTGTTCTGCTTGCGCAGACGCAAGGCTGGGCACTCCCCCGGGAGTGAGGGTTGCTGGGCCTGATGACGTGGCTTGGCAACGTCCCTACCGCCGCTGCTTCCCGGGAACCTGGCGCCGCCGGAACTGATCGCGGCCTAGTCCCGACGCGTGTGTGCTAGTGAGCCGGA [the ZTRE is underlined]) was performed using nuclear extract from Caco-2 cells maintained for 24 h in serum-free medium containing 100 μM zinc, as described previously (16). The band corresponding to the specific complex formed, which involved binding to the ZTRE sequence, as shown by competition using a 50-bp double-stranded oligonucleotide competitor containing the ZTRE (positions −124 to −75 of the *SLC30A5* gene) (16), was excised, cut into small pieces (ca. 1 mm^2^), and dried under vacuum. The gel pieces were then rehydrated on ice with 10 μl of digestion buffer (25 mM Tris-HCl [pH 8], 5 mM CaCl_2_; Promega) containing 25 ng of trypsin. After 10 min, a further 10 to 20 μl of the same buffer (without trypsin) was added to cover the gel slices, and the digestion was incubated for 16 h at 35°C. The resulting tryptic peptides were extracted twice using 10 to 20 μl of 0.1% trifluoroacetic acid in 60% acetonitrile at 56°C for 30 min, dried under vacuum, dissolved in 10 μl of 0.1% trifluoroacetic acid, and purified with Zip-Tip C18 pipette tips (Millipore), according to the manufacturer's recommended protocol. Peptides were eluted from the tip directly onto the matrix-assisted laser desorption ionization (MALDI) plate with matrix solution of alpha cyano-4-hydroxycinnamic acid (10 mg/ml) saturated in 50% acetonitrile and 0.1% trifluoroacetic acid.

EMSA using protein extract from Caco-2 cells transfected transiently with pCMV6-ZNF658-myc-DDK was used to demonstrate the binding of recombinant ZNF658 specifically to the ZTRE in *SLC30A5* by demonstrating supershifting of the specific band by anti-myc antibody. EMSA using protein extract from Caco-2 cells transfected transiently with pCMV6-ZNF658ΔZF12-15-myc-DDK was used to test the requirement for zinc fingers 12 to 15 for binding to the *SLC30A5* promoter. As described previously (16), the quantities of probe (positions −156 to +46 of the *SLC30A5* gene) and protein extract were 50 fmol and 5 μg, respectively, and the 50-bp oligonucleotide competitor that includes the ZTRE, when included in binding reactions, was used at a 200-fold excess over probe. Where included, anti-myc antibody (Sigma, catalog no. F1804) was added at 1 μg per binding reaction, followed by incubation for 20 min before loading.

An IRD-labeled probe corresponding to the same 212-bp region of the *SLC30A5* promoter used in the EMSA prior to band excision for analysis by mass spectrometry as described above (positions −156 to +46 relative to the TSS) but with the 5′ side of the ZTRE (corresponding to bases −91 to −84 relative to the TSS) deleted was generated as described previously for the equivalent probe without the deletion (16) but using plasmid pBlue*SLCC30A5*prom_Δ−91to−84_ (described above) in place of plasmid pBlue*SLCC30A5*prom.

### MALDI-time of flight (TOF) mass spectrometry.

The peptide mixture obtained by trypsin digestion of the protein sample prepared after EMSA of Caco-2 cell nuclear protein using the ZnT5 probe was analyzed using a Voyager DE-STR MALDI-TOF mass spectrometer (Applied Biosystems, Inc.). The instrument was equipped with a delayed extraction ion source, used a nitrogen laser at 337 nm and was operated in reflector mode at accelerating voltages of 20 to 25 kV. Mass spectra were obtained over a mass range of 900 to 4,000 Da, and monoisotopic peptide mass fingerprints were assigned and used for database searches. Identifications were performed using the peptide mass fingerprint data and the Mascot search engine program (Matrix Science, Ltd.) in which the peptide mass tolerance was limited to 50 ppm and searched against the MSDB (release 20063108, built 31 August 2006) protein sequence database.

### Measurement of relative RNA levels by RT-qPCR.

RNA was prepared from Caco-2 cells using TRIzol reagent (Invitrogen), and integrity was confirmed by using an Agilent 2100 bioanalyzer. RNA was treated with DNase (Roche; according to the manufacturer's instructions), and then first-strand cDNA synthesis was carried out using Superscript III RNase H^−^ reverse transcriptase (Invitrogen) and random hexamer primers (Promega), following the manufacturer's instructions. The relative levels of ZnT5, ZnT10, CBWD, ZNF658, GAPDH (glyceraldehyde-3-phosphate dehydrogenase), and β-actin mRNA and of 45S and 18S rRNA were measured using a Roche LightCycler 480 and SYBR green I master mix (Roche) with 20-μl reactions set up in 96-well format containing 0.5 μM concentrations of each primer. Primer sequences and thermal cycling parameters for ZnT5, ZnT10, CBWD, and GAPDH primers were as specified previously (16). The primers used to measure ZNF658 to confirm knockdown were as follows: GCTGCGCACCTGGGCTGAAC (forward) and CCCGGGTGAATTCCACAGTCACG (reverse). The primers for 45S rRNA were GTCAGGCGTTCTCGTCTCC (forward; bases 312 to 330 in NR_046235) and CGTCACCACATCGATCACGA (reverse; bases 446 to 427 in NR_046235). The primers for 18S rRNA were purchased from Primer Design, Southampton, United Kingdom (the sequences were not specified by the supplier), and amplify the 93-bp region comprising bases 92 to 184 in NR_003286. The primers for β-actin were CTGGAACGGTGAAGGTGACA (forward; bases 1359 to 1378 in NM_001101) and AAGGGACTTCCTGTAACAATGCA (reverse; bases 1455 to 1433 in NM_001101). Thermal cycling parameters were 95°C for 5 s, 60°C for 10 s, and 72°C for 10 s for 45 cycles. The ΔΔ*C_T_* method was used to calculate the relative levels of specific mRNAs at 100 μM compared to 3 μM zinc (standard serum-free culture medium) or where 2 μM TPEN was added to standard serum-free culture medium, with GAPDH or β-actin as the reference gene, as specified in Results.

### Western blotting.

Protein preparation, SDS-PAGE, blotting, and incubation with antibody (anti-myc; Sigma catalog no. F1804, used at a 1:1,000 dilution) were as described previously (19).

### Measurement of reporter gene expression.

Protein extracts were prepared from Caco-2 cells, and the activity of the β-galactosidase reporter gene was measured using chlorophenol red-β-d-galactopyranoside (CPRG) as the substrate, as described previously (18). The protein concentration of samples was measured using Bradford reagent (Bio-Rad Laboratories), and the relative specific activities were calculated.

### Statistical analysis.

Measurements of relative mRNA levels and of reporter gene activity at 100 μM zinc were expressed relative to the corresponding measurements made at 3 μM zinc or after the addition of 2 μM TPEN and then analyzed by one-way analysis of variance (ANOVA) and Tukey's posttest (for multiple comparisons) or by two-tailed Student unpaired *t* test (for single comparisons), taking *P* < 0.05 as significant.

### Analysis of RNA by hybridization to oligonucleotide microarrays.

Total RNA was isolated from Caco-2 cells (*n* = 3 each for cells transfected as described above with each of two siRNAs targeted to ZNF658 or with control siRNA) using a Pure Link RNA minikit (Ambion) following the manufacturer's protocol, and the RNA integrity was confirmed by using an Agilent bioanalyzer before hybridization to Illumina HT12 bead chip arrays. RNA labeling, amplification, and hybridization were performed by ServiceXS, Netherlands. Raw microarray scan files were exported using the Illumina Beadstudio program and loaded into R for downstream analysis. The data were transformed and normalized using the variance stabilizing normalization method. Probes with signals that fulfilled the criteria of Illumina probe detection *P* value of <0.01 were scored as positive. The data were then analyzed by the Newcastle Bioinformatics Support Unit. Significant changes of >1.2-fold were calculated using the rank product method.

### Accession number.

The data described above have been deposited in the Gene Expression Omnibus (GEO) database under accession number GSE59385.

## RESULTS

### Identification of ZNF658 by mass spectrometry as a protein that binds to the ZTRE.

**FIG 1:**
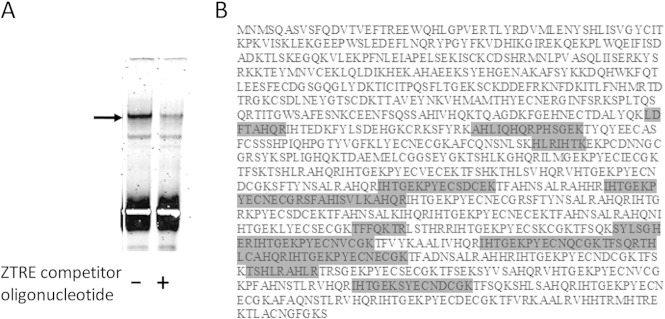
Identification of ZNF658 as a candidate ZTRE-binding protein. (A) Identification by EMSA of a specific band resulting from binding of a protein factor to the ZTRE in the SLC30A5 promoter that was subsequently analyzed by MALDI-TOF. An IRD-labeled probe (50 fmol), corresponding to the region from positions −156 to +46 of the *SLC30A5* gene was electrophoresed through a nondenaturing 5% polyacrylamide gel after incubation with protein extract prepared from Caco-2 cells. Competitor oligonucleotide, including the ZTRE (200-fold excess over probe) was included in the binding reaction as indicated. The arrow indicates the position of the band analyzed. (B) Peptides generated by trypsin-mediated hydrolysis of ZNF658 with masses corresponding to fragments detected by MALDI-TOF mass spectrometry in the protein mixture obtained from the band shown in panel A. The highlighted regions of protein sequence indicate the peptides, which cover 168 of the 1,058 amino acids (16%).

**FIG 2:**
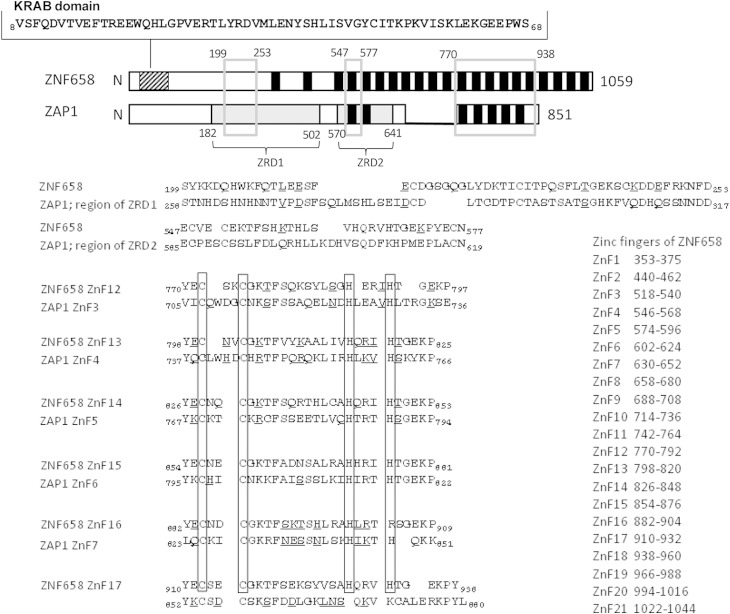
Alignment of ZNF658 and yeast ZAP1, highlighting regions of similarity. The 21 zinc fingers of ZNF658 and the 7 zinc fingers of ZAP1 are shown as black boxes. The KRAB domain of ZNF658 is indicated by a hatched box, and the amino acid sequence (positions 61 to 68) is shown. The zinc-responsive domains (ZRDs) of ZAP1 are shaded gray and labeled, with numbering indicating amino acid positions. Open boxes (gray lines) identify regions of similarity, with numbering indicating amino acid positions; alignments between these regions are shown below with identical amino acids highlighted in boldface, and conservative substitutions are indicated by underlining. For the region including zinc fingers 3 to 7 of ZAP1, the alignment with the corresponding region of ZNF658 is shown for each individual zinc finger, with the two cysteines and two histidines (or arginine) highlighted. The positions of the 21 zinc fingers of ZNF658 in the amino acid sequence are indicated.

We identified the ZTRE previously as a promoter sequence element involved in transcriptional repression of three different genes (*SLC30A5* [ZnT5 gene], *SLC30A10* [ZnT10 gene], and *CBWD*) in response to zinc (16). We identified the ZTRE sequence initially by virtue of observing that a specific band revealed by EMSA using the zinc-responsive region of the *SLC30A5* promoter was dependent on the retention of this sequence in the probe. Our approach to identify the transcriptional regulatory protein responsible for mediating zinc-induced transcriptional repression through the ZTRE was to excise this specific band from the gel and identify peptide fragments released by trypsin digestion by MALDI-TOF protein mass spectrometry. Alignment between proteins and peptides predicted by the mass profile using Mascot software (Matrix Science) detected ZNF658 (NM_033160.5) (*P* < 0.05; probability-based Mowse score > 64). Based on theoretical translation, ZNF685 is a protein of 1,059 amino acids. The peptide mass fragments corresponding to this protein covered 168 of these amino acids (16%) and numbered 16 of the 60 fragments that would be generated by trypsin-mediated hydrolysis of the protein (Fig. 1). When aligned with the Saccharomyces cerevisiae genome database (http://www.yeastgenome.org/) using BLASTP the alignment with the lowest E value (3.7e^−30^) was with ZAP1, the yeast zinc-dependent transcription factor responsible for coordinating the yeast transcriptional response to zinc-limiting conditions (20). Inspection of the sequence of ZNF658 (for the motif C-X_2_-C-X_12_-H-X_3_-H) indicates an unusually large number of zinc fingers, totaling 21 (of which the first has a HisCys-His_2_ configuration and the sixteenth has the unusual Cys_2_-His-Arg configuration). Zinc fingers 12 to 16 in ZNF658 align with the DNA binding domain (zinc fingers 3 to 7 [20]) of ZAP1 (Fig. 2). Inspection of the sequence of ZNF658 reveals the presence of a KRAB (Kruppel-associated box) domain at the N terminus (Fig. 2). Approximately one-third of human zinc finger proteins include this N-terminal domain, which typically represses transcription where it is bound by virtue of interaction with factors involved with chromatin remodeling (21).

### Knockdown of ZNF658 by siRNA abrogates or attenuates transcriptional repression by zinc of genes regulated through the ZTRE.

**FIG 3:**
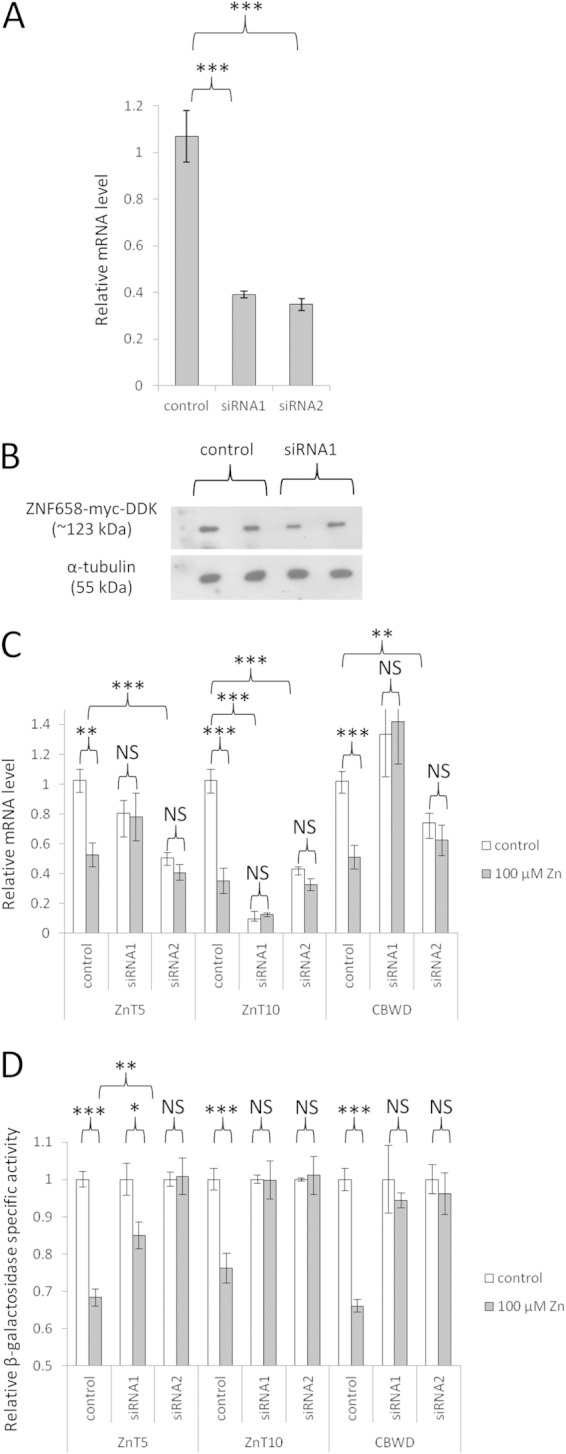
Effect of siRNA-mediated knockdown of ZNF658 on the response to zinc of ZnT5, ZnT10, and CBWD. (A) Confirmation of efficacy of siRNA-mediated knockdown of ZNF658 to reduce ZNF658 mRNA. Caco-2 cells were transfected with 10 nM either of two siRNAs targeted to ZNF658 (siRNA1 or siRNA2), or with the negative-control siRNA, and RNA was extracted after 48 h. ZNF658 mRNA was quantified by RT-qPCR using GAPDH as a reference gene. The data are means ± the SEM from three independent experiments (*n* = 7) for siRNA1 and from two independent experiments (*n* = 12) for siRNA2. ***, *P* < 0.001 (Student unpaired, two-tailed *t* test). (B) Confirmation of efficacy of siRNA-mediated knockdown of ZNF658 to reduce ZNF658 protein. Caco-2 cells were transfected with a plasmid construct to express recombinant ZNF658 with a C-terminal epitope tag (pCMV6-ZNF685-myc-DDK) and then with siRNA, and protein was analyzed by Western blotting with anti-myc or α-tubulin (loading control) antibody. Quantification by densitometry of signal intensities for myc expressed as a ratio of α-tubulin gave values of 1 ± 0.01 (control siRNA) versus 0.85 ± 0.03 (siRNA1) in means ± the standard deviations (*P* < 0.05 [Student unpaired two-tailed *t* test]). (C) Effect of siRNA-mediated knockdown of ZNF658 on mRNA levels. Caco-2 cells were transfected with 10 nM siRNA targeted to ZNF658 (or negative control) and treated with 100 μM zinc (or control) 24 h after transfection. RNA was extracted after a further 24 h, and the levels of the specific transcripts, relative to the level at the control (siRNA and zinc) condition, were measured by RT-qPCR using GAPDH as a reference gene. For siRNA1, data represent means ± the SEM from three independent experiments for ZnT5 (*n* = 4 to 6) and from two independent experiments (*n* = 3 or 4) for ZnT10 and CBWD. For siRNA2, data are means ± the SEM from two independent experiments (*n* = 6). **, *P* < 0.01; ***, *P* < 0.001 (one-way ANOVA and then Tukey's posttest; NS, not significant). (D) Effect of siRNA-mediated knockdown of ZNF658 on gene promoter activity. Caco-2 cells were cotransfected with 10 nM siRNA targeted to ZNF658 (or negative control) plus promoter reporter constructs comprising the region immediately upstream of and including part of the 5′ untranslated region (UTR) of each of the human ZnT5 (*SLC30A5*), ZnT10 (*SLC30A10*), and *CBWD* genes upstream of the β-galactosidase reporter gene in the vector pBlue-TOPO (Invitrogen). Cells were treated with 100 μM zinc 24 h after transfection, and the reporter gene activity was measured after a further 24 h and is expressed as a specific activity relative to total protein as determined using Bradford reagent, with bovine serum albumin (BSA) as the standard, which was then normalized for each gene and siRNA condition to the value measured at the lower zinc concentration. The data are means ± the SEM for 5 or 6 samples from two independent experiments for siRNA1 and for 3 samples from one experiment for siRNA2. *, *P* < 0.05; **, *P* < 0.01; ***, *P* < 0.001 (one-way ANOVA and then Tukey's posttest; NS, not significant).

The findings presented above indicated that ZNF685 was likely to be a zinc-dependent transcriptional regular that binds to the ZTRE. We thus investigated the effect of siRNA-mediated knockdown of ZNF658 on a panel of three genes known to respond to zinc through the ZTRE: *SLC30A5* (ZnT5 gene), *SLC30A10* (ZnT10 gene), and *CBWD* (16). Successful knockdown of ZNF658 in Caco-2 cells (by at least 60% compared to a control siRNA) was confirmed by RT-qPCR for each of two siRNAs targeted to different regions of the ZNF658 CDS used for these experiments (Fig. 3A). The effect of siRNA on expression of ZNF658 protein was investigated by Western blotting, using an anti-myc antibody and extract from cells expressing recombinant ZNF658 from a cytomegalovirus (CMV) promoter with a C-terminal myc epitope tag. Although the reduction in protein expression achieved was modest (15%; *P* < 0.05; Fig. 3B), as expected based on the high level of overexpression of the recombinant protein from the CMV promoter, together the data demonstrated efficacy of siRNA-mediated knockdown of ZNF658 protein, as well as mRNA. We used two complementary approaches to determine whether ZNF658 mediated transcriptional regulation of the panel of three genes in response to zinc. First, we sought to determine whether the reduced expression of ZNF658 abrogated the effect of zinc to reduce expression of each corresponding endogenous transcript. Second, we investigated whether the effect of zinc to repress expression of a reporter gene driven by *SLC30A5* (ZnT5 gene), *SLC30A10* (ZnT10 gene), and *CBWD* promoters in promoter-reporter constructs was affected by knockdown of ZNF658. Both approaches generated data consistent with ZNF658 being required for the response to zinc. The levels of all three mRNAs were reduced at 100 μM compared to 3 μM zinc in Caco-2 cells transfected with the control siRNA, replicating the response we measured previously (16). However, when expression of ZNF658 was reduced by either of the two targeted siRNAs these responses were abrogated (Fig. 3C). In addition, one of the two siRNAs (siRNA2) reduced the levels of ZnT5, ZnT10, and CBWD mRNAs. The other siRNA (siRNA1) had no effect on the levels of ZnT5 and CBWD mRNAs *per se* (only on their response to zinc) but reduced the expression of ZnT10 mRNA (Fig. 3C). Using the promoter-reporter constructs, the transcriptional repression in response to 100 μM zinc observed consistently in this experimental model (16) and reproduced in the current experiments in the presence of the control siRNA, was abrogated by ZNF658 knockdown using siRNA2 for all three test genes. When knockdown of ZNF658 was by siRNA1 the response to zinc was abrogated for ZnT10 and CBWD, whereas for ZnT5 the response was not abrogated but was attenuated (Fig. 3D).

### ZNF658 binds specifically to the ZTRE.

**FIG 4:**
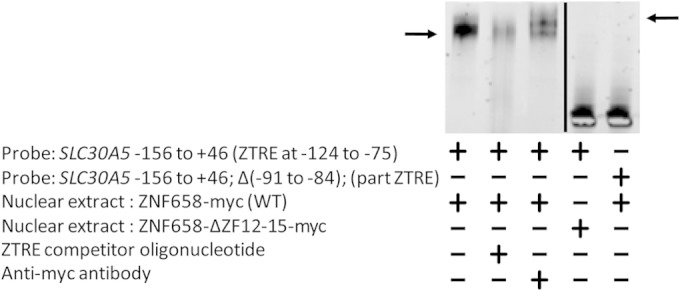
EMSA was performed to show directly the binding of ZNF658 to the ZTRE in *SLC30A5*and that zinc fingers 12 to 15 of ZNF658 plus both sides of the ZTRE are required for binding to occur. An IRD-labeled probe (50 fmol), corresponding to the region from −156 to +46 of the *SLC30A5* gene, or the same region excluding base pairs −91 to −84, as indicated, was electrophoresed through a nondenaturing 5% polyacrylamide gel after incubation with protein extract prepared from Caco-2 cells transfected to overexpress transiently wild-type ZNF658, a variant with the region corresponding to zinc fingers 12 to 15 deleted, or a negative-control protein (ZnT8) with the myc epitope as a C-terminal fusion. Competitor oligonucleotide, including the ZTRE (200-fold excess over probe), and/or anti-myc antibody was included in the binding reaction as indicated. The arrow on the left indicates the position of the specific complex between the probe and recombinant, myc-tagged ZNF658. The arrow on the right indicates the position of the supershifted band observed when anti-myc antibody was included in the binding reaction. The results are representative of multiple repeats of the experiments.

To demonstrate that ZNF658 binds specifically to the ZTRE, we carried out EMSA using as the labeled probe the same region of the *SLC30A5* promoter that we used in EMSA to identify this protein initially, as described above, and using protein extract from Caco-2 cells expressing recombinant ZNF658 with a C-terminal myc epitope tag. Competition by excess unlabeled double-stranded oligonucleotide that includes the ZTRE demonstrated specificity of the band observed (Fig. 4, lane 2). A major proportion of this band underwent a supershift when anti-myc antibody was included in the binding reaction, demonstrating that it represented a complex including the labeled probe with ZNF658 bound to the ZTRE (Fig. 4, lane 3).

### Zinc fingers 12 to 15 of ZNF658 are required for binding to the ZTRE.

Based on the alignment between the sequence of ZNF658 and yeast ZAP1, we proposed that the DNA binding domain includes zinc fingers 12 to 15. To test this idea, we deleted this region from the plasmid construct containing the ZNF658 coding sequence fused to a C-terminal myc epitope tag and expressed this recombinant protein in Caco-2 cells. Analysis by EMSA of the nuclear extract from these cells revealed that no complex formed with the probe containing the ZTRE (Fig. 4B, lane 5). At least one of the zinc fingers in the deleted region is thus necessary for the binding of ZNF655 to the ZTRE.

### One side of the palindromic ZTRE is insufficient as a binding target for ZNF658 or to mediate transcriptional repression in response to zinc.

**FIG 5:**
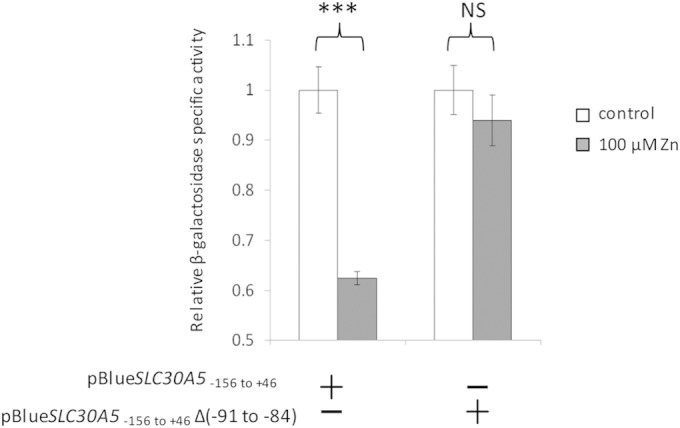
Effect of deleting the 3′ side of the ZTRE palindrome on the response of the *SLC30A5* promoter to zinc. Caco-2 cells were transfected with a promoter reporter construct comprising the region immediately upstream of and including part of the 5′ UTR of the human ZnT5 (*SLC30A5*) gene (−156 to +46 relative to the TSS) or with an equivalent construct in which the region −91 to −84, comprising the 3′ side of the ZTRE palindrome, was deleted, upstream of the β-galactosidase reporter gene in the vector pBlue-TOPO (Invitrogen). Cells were treated with 100 μM zinc 24 h after transfection, and the reporter gene activity was measured after a further 24 h and is expressed as the specific activity relative to the total protein as determined using Bradford reagent, with BSA as the standard, which was then normalized to the value measured at the lower zinc concentration. The data are means ± the SEM for *n* = 3. ***, *P* < 0.001 (one-way ANOVA and then Tukey's posttest; NS, not significant).

To advance our knowledge of the sequence specifications of functional ZTREs, we investigated whether both sides of the palindrome are necessary for the binding of ZNF658 and for mediating transcriptional repression in response to zinc. Deletion of the 3′ side of the ZTRE from the *SLC30A5* probe used for EMSA abrogated binding of recombinant ZNF658 (Fig. 4B, lane 6). Expression of the reporter gene from the *SLC30A5* promoter-reporter construct with the same region deleted became unresponsive to zinc (Fig. 5). Both sides of the ZTRE are thus required for the binding of ZNF658 and for transcription to be repressed by zinc.

### The profile of transcripts affected by reducing expression of ZNF658 indicates a role in the regulation of ribosome biogenesis.

**FIG 6:**
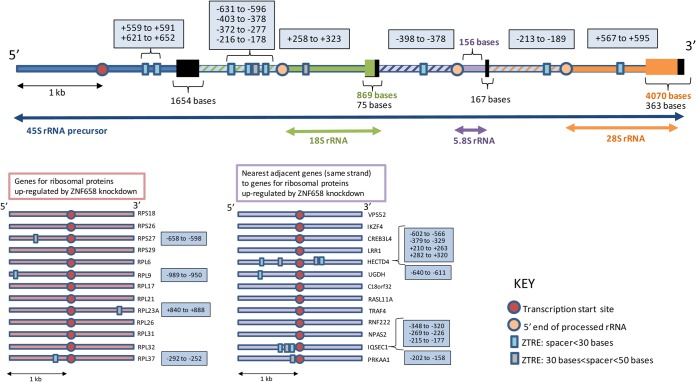
Clustering of ZTREs in the 45S rRNA precursor. Sequences within the regions mapped (1 kb 5′ and 3′ to a transcription start site or 5′ end of a processed rRNA, shown as color coded circles as in the key) that match the ZTRE [C-A/C-C-T/A/G-C-C-T/C-N(0-50)-G/A-G-A/T/C-G-T/G-G] are indicated as small boxes, color-coded as shown in the key to indicate whether the region between the two sides of the palindrome (N) is smaller than 30 bases or between 30 and 50 bases. ZTRE positions relative to either a transcription start site or 5′ end of a processed rRNA are given in the blue boxes. On the 45S rRNA precursor, the unmapped regions are collapsed and indicated by boxes with the sizes specified. The group of genes for ribosomal proteins upregulated by knockdown of ZNF658 and the group of their nearest adjacent neighbors are ordered such that they are paired (e.g., VPS52 is adjacent to RPS18). Note that the density of ZTREs in the mapped region of the 45S rRNA precursor is 10 in 14 kb (0.71/kb), whereas the densities in the mapped regions of the ribosomal protein genes and their nearest neighbors are 4 in 26 kb (0.15/kb) and 9 in 26 bp (0.35/kb), respectively (i.e., less than one-quarter and one-half, respectively).

To probe further the effects of ZNF658 on gene expression, we determined the effect of knockdown using siRNA on the transcriptome of Caco-2 cells. Seventy-seven probes on an Illumina HT12 bead chip microarray detected transcripts that were increased by knockdown of ZNF658 and 69 probes detected transcripts that were reduced (Table 1). rRNA 18S5 showed the biggest change in abundance, being reduced by 9.70-fold. rRNA28S5 was reduced by 2.6- to 1.68-fold. Also, 29 of the probes corresponding to transcripts increased by ZNF658 knockdown corresponded with ribosomal proteins or ribosomal protein pseudogenes. A search for the ZTRE motif “C-A/C-C-T/A/G-C-C-T/C-N(0-50)-G/A-G-A/T/C-G-T/G-G” 1 kb on either side of the TSS of the human 45S rRNA precursor and 1 kb on either side of the 5′ end of each of the mature rRNAs generated through its posttranscriptional processing (18S, 5.8S, and 28S rRNAs) revealed 10 copies (Fig. 6 and Fig. S1 in the supplemental material). We considered the possibility that the ribosomal protein pseudogenes upregulated by knockdown of ZNF658 are, in fact, functional genes repressed by ZNF658 when the zinc supply is adequate and expressed to substitute for other (zinc-dependent) ribosomal proteins when zinc supply is limited. However, a search for the ZTRE in the 1 kb of sequence on either side of the first exon in each of these 13 genes revealed copies in only two of the genes, so this scenario is unlikely. Microarray analysis does not distinguish reliably between ribosomal protein pseudogenes (which, by definition, should not be expressed) and expression of the corresponding functional ribosomal protein genes (22); thus, we assume that matches to ribosomal protein pseudogenes represent the corresponding functional gene. On this basis, our analysis detected 13 ribosomal protein genes expressed at higher levels under conditions of ZNF658 knockdown, and in which the ZTRE occurs infrequently (Table 1 and Fig. 6; see Fig. S2 and S3 in the supplemental material). The remarkable clustering of ZTREs in the rRNA precursor, along with the large changes in abundance of rRNAs brought about by reducing ZNF658 expression, is a strong indication that ZNF658 has a role (as yet undefined) in rRNA expression. In preliminary work we attempted to determine whether ZNF658 acting at ZTREs in the 45S rRNA precursor was required for transcription *per se* or for correct processing to the mature rRNAs. We used RT-qPCR to determine whether knockdown of ZNF658 caused a substantial shift in the relative proportion of the 45S rRNA precursor to 18S rRNA. The level of 18S rRNA was approximately 1.3 × 10^3^-fold greater than the level of the 45S precursor molecule, requiring the use of 20-fold less of the RT reaction for its measurement. The difference between *C_T_* values for the two molecules was not affected by knockdown of ZNF658 (6.54 ± 0.32 for control versus 6.36 ± 0.12 with knockdown of ZNF658 (means ± the standard errors of the mean [SEM]; *n* = 4). Thus, there was no change in relative abundance of the precursor and mature rRNAs of sufficient magnitude to be detectable using this assay. However, relative quantification was limited severely by the huge difference in abundance of the two species. The likelihood that the two rRNA species have very different half-lives further confounds interpretation of these data. Hence, we do not draw any conclusions at this stage. We also investigated whether zinc affected rRNA levels, using the 18S rRNA primers and β-actin as the reference gene. We observed that the level under conditions of zinc supplementation was ca. 50% of the level when zinc was depleted (using TPEN) (1.0 ± 0.06 versus 0.54 ± 0.06 [means ± the SEM, *n* = 6, *P* < 0.001]), a finding consistent with the ZTREs in the 45S rRNA precursor being functional with respect to mediating effects of zinc. We speculated that if ribosome biogenesis is controlled by the action of ZNF658 at the ZTRE, then the expression of RNA Pol I may also be regulated through this mechanism. Supporting this idea, we found two copies of the ZTRE close to the TSS in each of *POLR1D* and *POLR1E*, which code for two of the five subunits of RNA Pol I for which we found annotated genes (see Fig. S4 in the supplemental material).

**TABLE 1:**
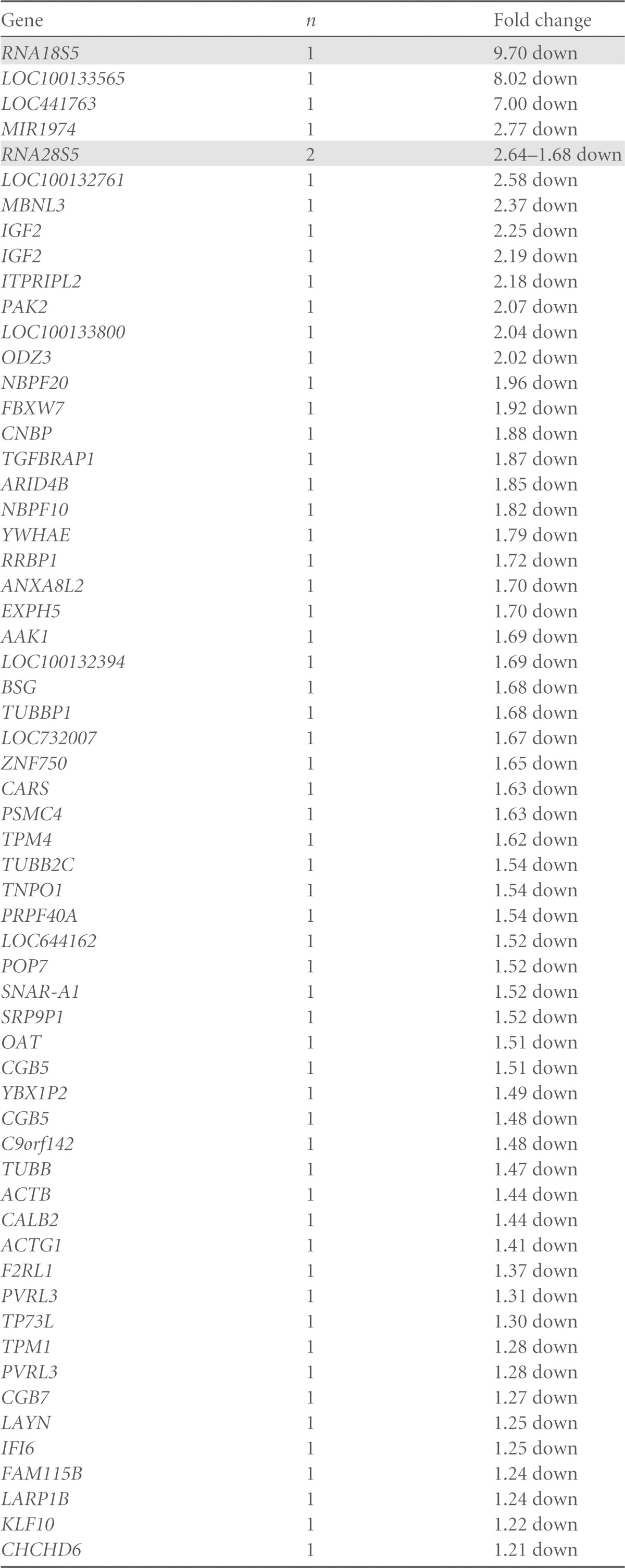

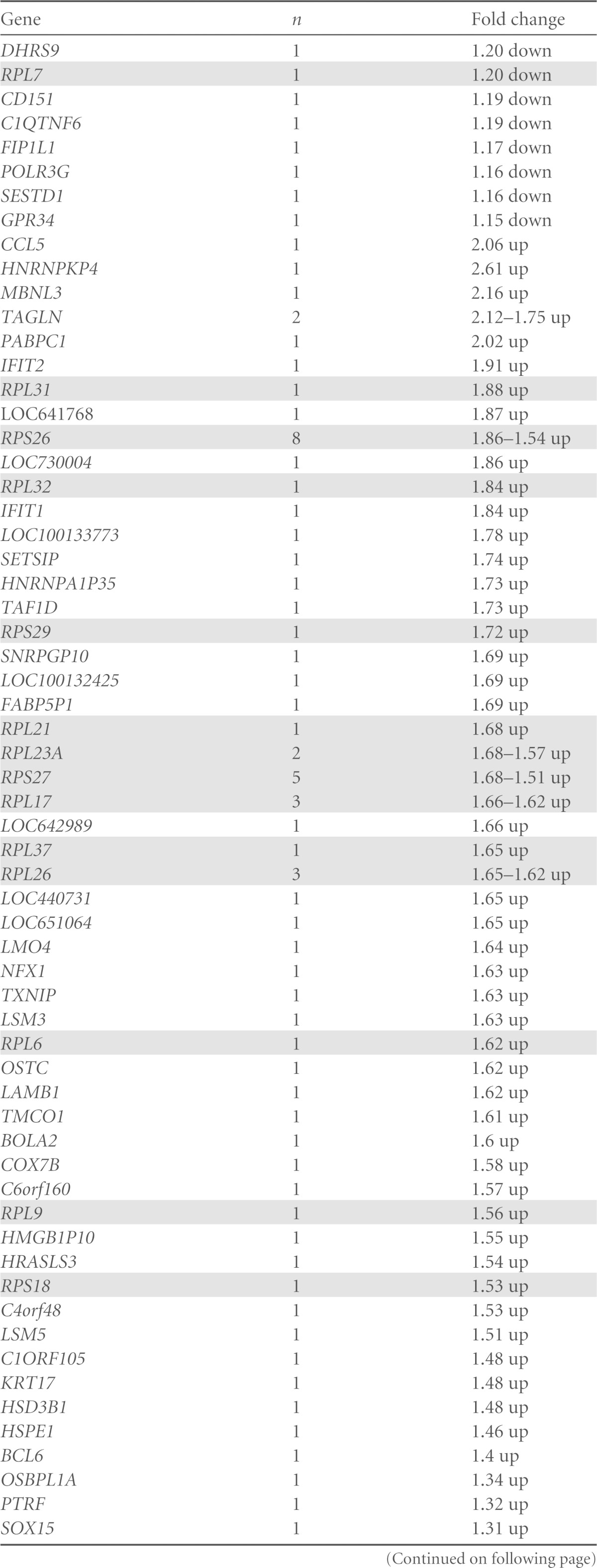

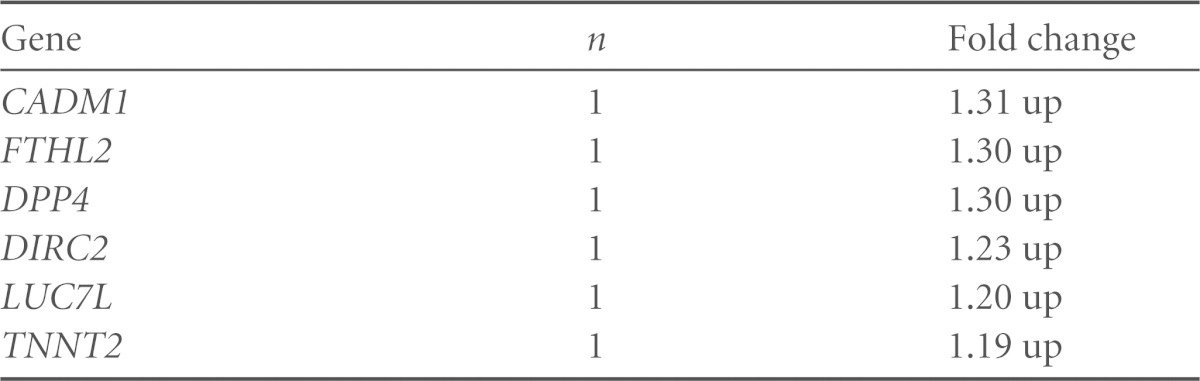
Transcripts detected at altered levels under conditions of ZNF658 knockdown compared to control^*a*^

a The genes listed are those for which transcript levels were detected at levels that differed by 1.2-fold (to 2 significant figures) or greater by hybridization of RNA to DNA oligonucleotide microarrays. Genes are ranked according to the magnitude of change detected, separated according to the direction of change (up or down). Data for genes corresponding to ribosomal proteins or ribosomal RNAs are shaded. The number of probes on the microarray that detected differential expression of each gene is stated (*n*). Probes listed as corresponding to ribosomal protein pseudogenes are considered to reveal expression of the corresponding bona fide gene and are grouped accordingly.

## DISCUSSION

We demonstrate that reduced expression of the zinc finger protein ZNF658, for which a specific function has not been reported previously, attenuated or abrogated an effect of zinc to repress expression of three genes that contain a functional copy of the ZTRE sequence. We also demonstrate that recombinant ZNF658 binds specifically to the ZTRE in a cell-free system. Previously, we showed that the ZTRE mediates transcriptional repression at elevated zinc concentrations (16). We thus show that ZNF658 plays a role in repressing transcription of these genes at higher zinc concentrations by binding to the ZTRE. In addition, we show that binding of ZNF658 to the ZTRE requires both sides of the palindromic sequence to be present and is through the zinc finger domain that corresponds with the DNA binding domain of ZAP1, the most similar protein in S. cerevisiae, which coordinates zinc-homeostatic gene expression changes when zinc becomes limiting. Moreover, we uncovered evidence that ZNF658 regulates ribosome biogenesis. These findings are of fundamental importance in understanding mechanisms of zinc homeostasis and consequences of its perturbation in metazoans. First, ZNF658 is the first identified transcription factor that represses the expression of multiple genes in response to zinc, thus providing the basis of a coordinated cellular response to restore zinc balance. Second, the involvement of ZNF658 in the control of ribosome biogenesis links directly this fundamental process into which a large proportion of zinc in the cell is channeled with zinc homeostasis.

The fact that ZAP1is the yeast protein most similar to ZNF658 may reflect common ancestry or acquisition of a similar DNA binding domain that is exquisitely sensitive to zinc occupancy. We confirm here that binding of ZNF658 to the ZTRE is through the zinc finger domain of ZNF658 that aligns with the DNA binding region of ZAP1. Each of the five zinc fingers in this region of ZAP1 is individually necessary for binding to DNA (20). Elucidation of whether or not any of the separate zinc fingers in the DNA binding domain of ZNF658 is dispensable for binding should be an avenue for future work to characterize the mechanism of ZNF658 function in more detail. The fact that ZAP1 is a transcriptional activator and ZNF658 an inhibitor is presumably attributable to the dissimilar regions of the two proteins interacting with regulatory proteins that have contrasting actions. A scenario in which ZNF658 acts at the ZTRE in complex with other proteins is likely given that regulation of transcription in general, including regulation in response to zinc through the MRE (23), is mediated by protein complexes, rather than single proteins.

Knockdown of ZNF658 also reduced the endogenous transcript level of the three genes we studied. With the exception of ZnT10, where both siRNAs reduced endogenous levels of transcript, this effect was observed only with siRNA2. The reason for this difference is unknown but, speculatively, may be due to the two siRNAs targeting different ZNF658 splice variants, which is formally possible since they target different exons (exon 5 for siRNA1 and the exon 3/4 boundary for siRNA2). We confirmed that siRNA1 reduced ZNF658 protein (as well as mRNA) and thus exclude lack of efficacy at the level of protein expression as the reason for the difference between the effects of the two siRNAs. The fact that the ZTRE in the *SLC30A10* (ZnT10) gene is located downstream of the transcription start site, but upstream in *SLC30A5* (ZnT5 gene) and *CBWD* (16), may be a reason for the difference.

When ZNF658 was expressed in cells at levels reduced by siRNA a number of transcripts were detected at higher levels by microarray analysis consistent with ZNF658 having a repressive effect on their expression, which is the classical action of KRAB-zinc finger proteins (21). However, other transcripts were detected at lower levels. These effects could be secondary to repression of direct gene targets of ZNF658. However, positive regulation of the expression of specific genes by ZNF658 should not be ruled out. Indeed, there are reports in the literature of KRAB-zinc finger proteins binding directly to genes in parallel with transcripts increasing in abundance (21, 24).

A surprising but important finding was that knockdown of ZNF658 resulted in a large (up to 9.7-fold) reduction in levels of rRNAs. This observation was authenticated by the discovery of multiple copies of the ZTRE in the 45S rRNA precursor. Two main factors point toward the role of ZNF658 in rRNA expression being at the level of RNA processing rather than transcription. First, the rRNA precursor is transcribed by RNA Pol I, whereas the proven gene targets of ZNF658 (*SLC30A5*, *SLC30A10*, and *CBWD*) are, like other protein-coding genes, transcribed by RNA Pol II. Although it appears not without precedent that a common transcription factor can be involved in the control of Pol I- and Pol II-mediated transcription (25), this appears an uncommon paradigm. Second, the ZTREs in the rRNA precursor sequence are clustered not around the TSS but, most densely, several hundred bases upstream of the sequence of the first mature transcript, 28S rRNA. It is not implausible that ZNF658 (possibly expressed as different splice variants) has a dual role, regulating both (Pol II-mediated) transcription of specific target genes and RNA processing given that other members of the KRAB-domain containing ZNF protein family appear to have such a dual role (21, 26–28). An effect on rRNA abundance of changing posttranscriptional processing could be due to differences in turnover rates of the precursor versus the mature transcripts. The position within the 45S rRNA precursor sequence of the three probes on the array through which a change in rRNA expression was detected (two within the 18S rRNA sequence and one within the 28S rRNA sequence; see Fig. S1 in the supplemental material) means that the analysis would not distinguish between a change in the abundance of the full 45S rRNA precursor and a change in processing to mature rRNAs with different half-lives. However, *C_T_* values derived using RT-qPCR indicated that the 45S rRNA precursor was at a level more than 3 orders of magnitude lower than the processed 18S rRNA transcript. Thus, the effect detected is likely to reflect a change in the levels of the mature rRNAs. This large difference in abundance between the 45S rRNA precursor and the processed 18S rRNA product confounded our attempt to detect whether the ratio of the precursor to mature product was affected by knockdown of ZNF658 using RT-qPCR. Further work is thus required to provide insight into the precise function of ZNF658 in rRNA biosynthesis by determining whether 45S rRNA transcription or processing is affected by knockdown of this factor. A battery of approaches is likely to be required to tease out the affected points and could include metabolic labeling of nascent 45S rRNA with [^3^H]uridine, nuclear run-on measurements, psoralen cross-linking (to distinguish rRNA genes undergoing active transcription from genes in a repressed chromatic configuration), and RNA Pol I chromatin immunoprecipitation (ChIP) (29).

Zinc is essential for ribosome biogenesis because RNA Pol I, like the other major eukaryotic RNA polymerases, requires zinc (30, 31), and also because zinc-binding proteins are abundant in the ribosome. Our discovery of ZTREs in the genes for subunits D and E of RNA Pol I in positions likely to confer zinc-responsive transcriptional control suggests that control of RNA Pol I biosynthesis is an additional mechanism through which ZNF658 regulates ribosome biogenesis according to zinc availability. It has been suggested that the suppression of ribosome biogenesis seen in yeast under conditions of zinc deficiency is a mechanism to prioritize the synthesis of essential RNA Pol II-transcribed proteins (32). There is thus strong rationale for there being a tight link between the regulation of ribosome biogenesis and cellular processes for zinc homeostasis. The indications we uncovered that ZNF658 acting through the ZTRE coordinates these two processes highlights the urgent need to elucidate the details of this process, which is potentially of substantial fundamental importance in the understanding of basic cell function with possible widespread translational implications, for example, in better harnessing the ability of cells to express commercially important proteins.

An effect on ribosome biogenesis of knocking down expression of ZNF658 was indicated also by a change in the expression of a raft of ribosomal protein genes. The ZTRE occurs at even lower frequency in the region around the TSS of these ribosomal protein genes than it does in their closest adjacent genes, suggesting that the ribosomal protein genes are not direct targets of ZNF658 and that these changes in expression are secondary to other perturbed processes. Thus, the fact that expression of these genes was increased, opposing the effect on rRNAs, may be a compensatory response driven by mechanisms separate from ZNF658. Linkage of these observations in this manner requires that the control of ribosome biogenesis in mammalian cells differs from yeast, where it has been reported that the activity of RNA Pol I determines and coordinates the RNA Pol II-mediated expression of the RP genes (33). Given that the process in yeast and mammals differs in several ways, this scenario is not implausible.

In summary, we report here for the first time the identity of a protein, ZNF658, that has a dual function in cellular zinc homeostasis and allocation in metazoans. First, ZNF658 can coordinate a homeostatic response to changes in cellular zinc availability through effects on the transcription of multiple genes. Second, the same protein can act on rRNA to affect ribosome biogenesis, a process into which a substantial proportion of zinc in the cell is allocated. We identify a specific region of zinc fingers necessary for binding to the double-stranded DNA target. To uncover more details about the actions of ZNF658 in metal homeostasis, future work should probe the identity of putative proteins that interact with ZNF658 (for example, by coimmunoprecipitation), explore the full range and identity of nucleic acid targets to which ZNF658 binds (for example, by ChIP-seq, rRNA-targeted ChIP approaches, and UV cross-linking, partial RNase digestion followed by sequencing of cDNA [CRAC]), and elucidate how the individual zinc fingers and domains of the protein contribute to its function (for example, by further mutagenesis and also the application of biophysical techniques to probe structure and zinc binding). The function of *CBWD* genes, regulated by zinc through this mechanism, has not been elucidated. However, our current research indicates a role in protecting cells against extremes of zinc concentration, a finding consistent with a homeostatic function, which we are currently probing in more depth.

## Supplementary Material

Supplemental material
