# Supplementary material for: The Zinc Finger Protein ZNF658 Regulates the Transcription of Genes Involved in Zinc Homeostasis and Affects Ribosome Biogenesis through the Zinc Transcriptional Regulatory Element
Source: Mol Cell Biol. 2015 Feb 18;35(6):977–87. doi: 10.1128/MCB.01298-14 (PMC4333095; doi:10.1128/MCB.01298-14)
Supplement: Supplemental material [file supp_35_6_977__index.html]

The Zinc Finger Protein ZNF658 Regulates the Transcription of Genes Involved in Zinc Homeostasis and Affects Ribosome Biogenesis through the Zinc Transcriptional Regulatory Element — Supplemental material 

# The Zinc Finger Protein ZNF658 Regulates the Transcription of Genes Involved in Zinc Homeostasis and Affects Ribosome Biogenesis through the Zinc Transcriptional Regulatory Element

## Supplemental material

**Files in this Data Supplement:**

- Supplemental file 1 -

  Fig. S1 (Sequence of human 45S rRNA precursor RNA45S5)

  PDF, 181K
- Supplemental file 2 -

  Fig. S2 (Occurrence of ZTRE for ribosomal protein genes)

  PDF, 264K
- Supplemental file 3 -

  Fig. S3 (Occurrence of ZTRE for genes adjacent to ribosomal protein genes)

  PDF, 318K
- Supplemental file 4 -

  Fig. S4 (Occurrence of ZTRE for RNA pol I subunit genes)

  PDF, 262K
- Supplemental file 5 -

  Table S1 (siRNAs used to knock down expression of ZNF658)

  PDF, 217K
