# Supplementary material for: The Zinc Finger Protein ZNF658 Regulates the Transcription of Genes Involved in Zinc Homeostasis and Affects Ribosome Biogenesis through the Zinc Transcriptional Regulatory Element
Source: Mol Cell Biol. 2015 Feb 18;35(6):977–87. doi: 10.1128/MCB.01298-14 (PMC4333095; doi:10.1128/MCB.01298-14)
Supplement: Supplemental material [file MCB.01298-14_zmb999100760so1.pdf]

CTCGGGAAGAGCTTCTCGACTCACGGTTTCGCTTTCGCGGTCCACGGGCCGCCCTGCCAGCCGGATCTGTCTCGC
TGACGTCCGCGGCGGTTGTCGGGCTCCATCTGGCGGCCGCTTTGAGATCGTGCTCTCGGCTTCCGGAGCTGCGGT
GGCAGCTGCCGAGGGAGGGGACCGTCCCCGCTGTGAGCTAGGCAGAGCTCCGGAAAGCCCGCGGTCGTCAGCCCG
GCTGGCCCGGTGGCGCCAGAGCTGTGGCGCGTCGCTTGTGAGTCACAGCTCTGGCGTGCAGGTTTATGTGGGGGA
GAGGCTGTCGCTGCGCTTCTGGGCCCGCGGCGGGCGTGGGGCTGCCCGGGCCGGTCGACCAGCGCGCCGTAGCTC
CCGAGGCCCGAGCCGCGACCCGCGGGACCCGCCGCGCGTGGCGCGGGAGGCTGGGGACGCCCTTCCCGGCCCGG
TCGCGGGTCCGCGCTCATCCTGGCCGTCTGAGGCGGCGGCCGAATTCGTTTCCGAGTCCCCGTGGGAGCCGGGG
ACCGTCCCGCCCCCGTCCCCCGGGTGCCGGGGAGCGGTCCCCGGGCCGGGCCGCGGTCCCTCTGCCGCGATCCTT
TCTGGCGAGTCCCCGTGCGGAGTCGGAGAGCGCTCCCTGAGCGCGCGTGCGGCCCGAGAGGTCGCGCCTGGCCGG
CCTTCGGTCCCTCGTGTGTCCCGGTCGTAGGAGGGGCCGGCCGAAAATGCTTCCGGCTCCCGCTCTGGAGACACG
GGCCGGCCCCCTGCGTGTGGCACGGGCGGCCGGGAGGGCGTCCCCGGCCCGGCGCTGCTCCCGCGTGTGTCCTGG
GGTTGACCAGAGGGCCCCGGGCGCTCCGTGTGTGGCTGCGATGGTGGCGTTTTTGGGGACAGGTGTCCGTGTCGC
GCGTCGCCTGGGCCGGCGGCGTGGTCGGTGACGCGACCTCCCGGCCCCGGGGGAGGTATATCTTTCGCTCCGAGT
CGGCATTTTGGGCCGCCGGGTTATTGCTGACACGCTGTCCTCTGGCGACCTGTCGCTGGAGAGGTTGGGCCTCCG
GATGCGCGCGGGGCTCTGGCCTACCGGTGACCCGGCTAGCCGGCCGCGCTCCTGCTTGAGCCGCCTGCCGGGGCC
CGCGGGCCTGCTGTTCTCTCGCGCGTCCGAGCGTCCCGACTCCCGGTGCCGGCCCGGGTCCGGGTCTCTGACCCA
CCCGGGGGCGGCGGGGAAGGCGGCGAGGGCCACCGTGCCCCCGTGCGCTCTCCGCTGCGGGCGCCCGGGGCGGCC
GCGACAACCCCACCCCGCTGGCTCCGTGCCGTGCGTGTCAGGCGTTCTCGTCTCCGCGGGGTTGTCCGCCGCCCC
TTCCCCGGAGTGGGGGGTTGGCCGGAGCCGATCGGCTCGCTGGCCGGCCGGCCGGCCTCCGCTCCCGGGGGGCTC
TTCGTGATCGATGTGGTGACGTCGTGCTCTCCCGGGCCGGGTCCGAGCCGCGACGGGCGAGGGGCGGACGTTCGT
GGCGAACGGGACCGTCCTTCTCGCTCCGCCCCGCGGGGGTCCCCTCGTCTCTCCTCTCCCCGCCCGCCGGCGGTG
CGTGTGGGAAGGCGTGGGGTGCGGACCCCGGCCCGACCTCGCCGTCCCGCCCGCCGCCTTCTGCGTCGCGGGGCG
GGCCGGCGGGGTCCTCTGACGCGGCAGACAGCCCTCGCTGTCGCCTCCAGTGGTTGTCGACTTGCGGGCGGCCCC
CCTCCGCGGCGGTGGGGGTGCCGTCCCGCCGGCCCGTCGTGCTGCCCTCTCGGGGGGTTTGCGCGAGCGTCGGCT
CCGCCTGGGCCCTTGCGGTGCTCCTGGAGCGCTCCGGGTTGTCCCTCAGGTGCCCGAGGCCGAACGGTGGTGTGT
CGTTCCCGCCCCCGGCGCCCCCTCCTCCGGTCGCCGCCGCGGTGTCCGCGCGTGGGTCCTGAGGGAGCTCGTCGG
TGTGGGGTTCGAGGCGGTTTGAGTGAGACGAGACGAGACGCGCCCCTCCCACGCGGGGAAGGGCGCCCGCCTGCT
CTCGGTGAGCGCACGTCCCGTGCTCCCCTCTGGCGGGTGCGCGCGGGCCGTGTGAGCGATCGCGGTGGGTTCGGG
CCGGTGTGACGCGTGCGCCGGCCGGCCGCCGAGGGGCTGCCGTTCTGCCTCCGACCGGTCGTGTGTGGGTTGACT
TCGGAGGCGCTCTGCCTCGGAAGGAAGGAGGTGGGTGGACGGGGGGGCCTGGTGGGGTTGCGCGCACGCGCGCAC
CGGCCGGGCCCCCGCCCTGAACGCGAACGCTCGAGGTGGCCGCGCGCAGGTGTTTCCTCGTACCGCAGGGCCCCC
TCCCTTCCCCAGGCGTCCCTCGGCGCCTCTGCGGGCCCGAGGAGGAGCGGCTGGCGGGTGGGGGGAGTGTGACCC
ACCCTCGGTGAGAAAAGCCTTCTCTAGCGATCTGAGAGGCGTGCCTTGGGGGTACCGGATCCCCCGGGCCGCCGC
CTCTGTCTCTGCCTCCGTTATGGTAGCGCTGCCGTAGCGACCCGCTCGCAGAGGACCCTCCTCCGCTTCCCCCTC
GACGGGGTTGGGGGGGAGAAGCGAGGGTTCCGCCGGCCACCGCGGTGGTGGCCGAGTGCGGCTCGTCGCCTACTG
TGGCCCGCGCCTCCCCCTTCCGAGTCGGGGGAGGATCCCGCCGGGCCGGGCCCGGCGTTCCCAGCGGGTTGGGAC
GCGGCGGCCGGCGGGCGGTGGGTGTGCGCGCCCGGCGCTCTGTCCGGCGCGTGACCCCCTCCGCCGCGAGTCGGC
TCTCCGCCCGCTCCCGTGCCGAGTCGTGACCGGTGCCGACGACCGCGTTTGCGTGGCACGGGGTCGGGCCCGCCT
GGCCCTGGGAAAGCGTCCCACGGTGGGGGCGCGCCGGTCTCCCGGAGCGGGACCGGGTCGGAGGATGGACGAGAA
TCACGAGCGACGGTGGTGCGGGCGTGTCGGGTTCGTGGCTGCGGTCGCTCCGGGGCCCCCGGTGGCGGGGCCCCG
GGGCTCGCGAGGCGGTTCTCGGTGGGGGCCGAGGGCCGTCCGGCGTCCCAGGCGGGGCGCCGCGGGACCGCCCTC
GTGTCTGTGGCGGTGGGATCCCGCGGCCGTGTTTTCCTGGTGGCCCGGCCGTGCCTGAGGTTTCTCCCCGAGCCG
CCGCCTCTGCGGGCTCCCGGGTGCCCTTGCCCTCGCGGTCCCCGGCCCTCGCCCGTCTGTGCCCTCTTCCCCGCC
CGCCGCCCGCCGATCCTCTTCTTCCCCCCGAGCGGCTCACCGGCTTCACGTCCGTTGGTGGCCCCGCCTGGGACC
GAACCCGGCACCGCCTCGTGGGGCGCCGCCGCCGGCCACTGATCGGCCCGGCGTCCGCGTCCCCCGGCGCGCGCC
TTGGGGACCGGGTCGGTGGCGCCCCGCGTGGGGCCCGGTGGGCTTCCCGGAGGGTTCCGGGGGTCGGCCTGCGGC
GCGTGCGGGGGAGGAGACGGTTCCGGGGGACCGGCCGCGACTGCGGCGGCGGTGGTGGGGGCAGCCGCGGGGATC
GCCGAGGGCCGGTCGGCCGCCCCGGGTGCCGCGCGGTGCCGCCGGCGGCGGTGAGGCCCCGCGCGTGTGTCCCGG
CCGCGGTCGGCCGCGCTCGAGGGGTCCCCGTGGCGTCCCCTTCCCCGCCGGCCGCCTTTCTCGCGCCTTCCCCGT
CGCCCCGGCCTCGCCCGTGGTCTCTCGTCTTCTCCCGGCCCGCTCTTCCGAACCGGGTCGGCGCGTCCCCCGGGT
GCGCCTCGCTTCCCGGGCCTGCCGCGGCCCTTCCCCGAGGCGTCCGTCCCGGGCGTCGGCGTCGGGGAGAGCCCG
TCCTCCCCGCGTGGCGTCGCCCCGTTCGGCGCGCGCGTGCGCCCGAGCGCGGCCCGGTGGTCCCTGCCGGACAGG
CGTTCGTGCGACGTGTGGCGTGGGTCGACCTCCGCCTTGCCGGTCGCTCGCCCTTTCCCCGGGTCGGGGGGTGGG
GCCCGGGCCGGGGCCTCGGCCCCGGTCGCGGTCCCCCGTCCC**GGGCGGG**GGCGGGCGCGCCGGCCGGCCTCGGTC
GGCCCTCCCTTGGCCGTCGTGTGGCGTGTGCCACCCCTGCGCCCGCGCCCGCCGGCGGGGCTCGGAGCCGGGCTT
CGGCCGGGCCCCGGGCCCTCGACCGGACCGGTGCGCGGGCGCTGCGGCCGCACGGCGCGACTGTCCCCGGGCCGG
GCACCGCGGTCCGCCTCTCGCTCGCCGCCCGGACGTCGGGGCCGCCCCGCGGGGCGGGCGGAGCGCCGTCCCCGC
CTCGCCGCCGCCCGCGGGCGCCGGCCGCGCGCGCGCGCGTGGCCGCCGGTCCCTCCCGGCCGCCGGGCGCGGG
TCGGGCCGTCCGCCTCCTCGCGGGCGGGCGCGACGAAGAAGCGTCGCGGGTCTGTGGCGCGGGGCCCCGGTGGTC
GTGTCGCGTGGGGGGCGGGTGGTTGGGGCGTCCGGTTCGCCGCGCCCCGCCCCGGCCCCACCGGTCCCGGCCGCC

GCCCCCGCGCCCGCTCGCTCCCTCCCGTCCGCCCGTCCGCGGCCCGTCCGTCCGTCCGTCGTCCTCCTCGCTTGC
GGGGCGCCGGGCCCGTCCTCGCGAGGCCCCCCGGCCGGCCGTCCGGCCGCGTCGGGGCCTCGCCGCGCTCTACCT
TACCTACCTGGTTGATCCTGCCAGTAGCATATGCTTGTCTCAAAGATTAAGCCATGCATGTCTGAGTACGCACGG
CCGGTACAGTGAAACTGCGAATGGCTCATTAAATCAGTTATGGTTCCTTTGGTCGCTCGCTCCTCTCCTACTTGG
ATAACTGTGGTAATTCTAGAGCTAATACATGCCGACGGGCGCTGACCCCCTTCGCGGGGGGGATGCGTGCATTTA
TCAGATCAAAACCAACCCGGTCAGCCCCTCTCCGGCCCCGGCCGGGGGGCGGGCGCCGGCGGCTTTGGTGACTCT
AGATAACCTCGGGCCGATCGCACGCCCCCCGTGGCGGCGACGACCCATTCGAACGTCTGCCCTATCAACTTTCGA
TGGTAGTCGCCGTGCCTACCATGGTGACCACGGGTGACGGGGAATCAGGGTTCGATTCCGGAGAGGGAGCCTGAG
AAACGGCTACCACATCCAAGGAAGGCAGCAGGCGCGCAAATTACCCACTCCCGACCCGGGGAGGTAGTGACGAAA
AATAACAATACAGGACTCTTTCGAGGCCCTGTAATTGGAATGAGTCCACTTTAAATCCTTTAACGAGGATCCATT
GGAGGGCAAGTCTGGTGCCAGCAGCCGCGGTAATTCCAGCTCCAATAGCGTATATTAAAGTTGCTGCAGTTAAAA
AGCTCGTAGTTGGATCTTGGGAGCGGGCGGGCGGTCCGCCGCGAGGCGAGCCACCGCCCGTCCCCGCCCCTTGCC
TCTCGGCGCCCCCTCGATGCTCTTAGCTGAGTGTCCCGCGGGGCCCGAAGCGTTTACTTTGAAAAAATTAGAGTG
TTCAAAGCAGGCCCGAGCCGCCTGGATACCGCAGCTAGGAATAATGGAATAGGACCGCGGTTCTATTTTGTTGGT
TTTCGGAACTGAGGCCATGATTAAGAGGGACGGCCGGGGGCATTCGTATTGCGCCGCTAGAGGTGAAATTCTTGG
ACCGGCGCAAGACGGACCAGAGCGAAAGCATTTGCCAAGAATGTTTTCATTAATCAAGAACGAAAGTCGGAGGTT
CGAAGACGATCAGATACCGTCGTAGTTCCGACCATAAACGATGCCGACCGGCGATGCGGCGGCGTTATTCCCATG
ACCCGCCGGGCAGCTTCCGGGAAACCAAAGTCTTTGGGTTCCGGGGGGAGTATGGTTGCAAAGCTGAAACTTAAA
GGAATTGACGGAAGGGCACCACCAGGAGTGGAGCCTGCGGCTTAATTTGACTCAACACGGGAAACCTCACCCGGC
CCGGACACGGACAGGATTGACAGATTGATAGCTCTTTCTCGATTCCGTGGGTGGTGGTGCATGGCCGTTCTTAGT
TGGTGGAGCGATTTGTCTGGTTAATTCCGATAACGAACGAGACTCTGGCATGCTAACTAGTTACGCGACCCCCGA
GCGGTCGGCGTCCCCCAACTTCTTAGAGGGACAAGTGGCGTTCAGCCACCCGAGATTGAGCAATAACAGGTCTGT
GATGCCCTTAGATGTCCGGGGCTGCACGCGCGCTACACTGACTGGCTCAGCGTGTGCCTACCCTACGCCGGCAGG
CGCGGGTAACCCGTTGAACCCCATTCGTGATGGGGATCGGGGATTGCAATTATTCCCCATGAACGAGGAATTCCC
AGTAAGTGCGGGTCATAAGCTTGCGTTGATTAAGTCCCTGCCCTTTGTACACACCGCCCGTCGCTACTACCGATT
GGATGGTTTAGTGAGGCCCTCGGATCGGCCCCGCCGGGGTCGGCCCACGGCCCTGGCGGAGCGCTGAGAAGACGG
TCGAACTTGACTATCTAGAGGAAGTAAAAGTCGTAACAAGGTTTCCGTAGGTGAACCTGCGGAAGGATCATTAAC
GGAGCCCGGAGGGCGAGGCCCGCGGCGGCGCCGCCGCCGCCGCGCGCTTCCCTCCGCACACCCACCCCCCCACCG
CGACGCGGCGCGTGCGCGGGCGGGCCCGCGTGCCCGTTCGTTCGCTCGCTCGTTCGTTCGCCGCCCGGCCCCGC
CGGCCGCGAGAGCCGGAGAACTCGGGAGGGAGACGGGGGAGAGAGAGAGAGAGAGAGAAAGAGAAAGAAGGGCGT
GTCGTTGGTGTGCGCGTGTCGTGGGGCCGGCGGGCGGCGGGGAGCGGTCCCCGGCAGCGGCCCCGACGGCGTGGG
TGTCGGCGGGCGCGGGGGCGGTTCTCGGCGGCGTCGCGGCGGGTCTGGGGGTCTCGGTGCCCTCCTCCCCGCCGG
GGCCCGTCGTCCGGCCCCGCCGCGCCGGCTCCCCGTCTTCGGGGCCGGCCGGATTCCCGTCGCCTCCGCCGCGCC
GCTCCGCGCCGCCGGGCACGGCCCCGCTCGCTCTCCCCGGCCTTCCCGCTAGGGCGTCTCGAGGGTCGGGGGCCG
GACGCCGGTCCCCTCCCCCGCCTCCTCGTCCGCCCCCCCGCCGTCCAGGTACCTAGCGCGTTCCGGCGCGGAGGT
TTAAAGACCCCTTGGGGGGATCGCCCGTCCGCCCGTGGGTCGGGGGCGGTGGTGGGCCCGCGGGGGAGTCCCGTC
GGGAGGGGCCCGGCCCCTCCCGCGCCTCCACCGCGGACTCCGCTCCCCGGCCGGGGCCGCGCCGCCGCCGCCGCC
GCGGCGGCCGTCGGGTGGGGGCTTTACCCGGCGGCCGTCGCGCGCCTGCCGCGCGTGTGGCGTGCGCCCCGCGCC
GTGGGGGCGGGAACCCCCGGGCGCCTGTGGGGTGGTGTCCGCGCTCGCCCCCGCGTGGGCGGCGCGCGCCTCCCC
GTGGTGTGAAACCTTCCGACCCCTCTCCGGAGTCCGGTCCCGTTTGCTGTCTCGTCTGGCCGGCCTGAGGCAACC
CCCTCTCCTCTTGGGGGGGGCGGGGGGACGTGCCGCGCCAGGAAGGCCTCCTCCCGGTGCGTCGTCGGGAGCG
CCCTCGCCAAATCGACCTCGTACGACTCTTAGCGGTGGATCACTCGGCTCGTGCGTCGATGAAGAACGCAGCTAG
CTGCGAGAATTAATGTGAATTGCAGGACACATTGATCATCGACACTTCGAACGCACTTGCGGCCCCGGGTTCCTC
CCGGGGCTACGCCTGTCTGAGCGTCGCTTGCCGATCAATCGCCCCCGGGGGTGCCTCCGGGCTCCTCGGGGTGCG
CGGCTGGGGGTTCCCTCGCAGGGCCCGCCGGGGGCCCTCCGTCCCCCTAAGCGCAGACCCGGCGGCGTCCGCCCT
CCTCTTGCCGCCGCGCCCGCCCCTTCCCCCTCCCCCCGCGGGCCCTGCGTGGTCACGCGTCGGGTGGCGGGGGG
AGAGGGGGGCGCGCCCGGCTGAGAGAGACGGGGAGGGCGGCGCCGCCGCCGCCCGCGAAGACGGAGAGGGAAAGA
GAGAGCCGGCTCGGGCCGAGTTCCCGTGGCCGCCGCCTGCGGTCCGGGTTCCTCCCTCGGGGGGCTCCCTCGCGC
CGCGCGCGGCTCGGGGTTCGGGGTTCGTCGGCCCCGGCCGGGTGGAAGGTCCCGTGCCCGTCGTCGTCGTCGTCG
TCGCGCGTCGTCGGCGGTGGGGGCGTGTTGCGTGCGGTGTGGTGGTGGGGGAGGAGGAAGGCGGGTCCGGAAGGG
GAAGGGTGCCGGCGGGGAGAGAGGGTCGGGGGAGCGCGTCCCGGTCGCCGCGGTTCGCCGCCCGCCCCCGGTGGC
GGCCCGGCGTCCGGCCGACCGCCGCTCCCGCGCCCCTCCTCCTCCCCGCCGCCCCTCCTCCGAGGCCCCGCCCGT
CCTCCTCGCCCTCCCCGCGCGTACGCGCGCCCGCCCGCCCGGCTCGCCTCGCGGCGCGTCGGCCGGGGCCGGGAG
CCCGCCCCGCGGCCCGCCCGGCCGCGCCCGTGGCCGCGGCGCCGGGGTTCGCGTGTCCCCGGCGGCGACCCGCGG
GACGCCGCGGTGTCGTCCGCCGTCGCGCGCCCGCCTCCGGCTCGCGGCCGCGCCGCGCCGCGCCGGGGCCCCGTC
CCGAGCTTCCGCGTCGGGGCGGGGCGGCTCCGCCGCCGCGTCCTCGGACCCGTCCCCCCGACCTCCGCGGGGGAG
ACGGGTCGGGGCGTGCGGCGCCCGTCCCGCCCCCGGCCCGTGCCCCTCCCTCCGGTCGTCCCGCTCCGGCGGGGC
GGCGCGGGGGTGCCGCCGGCCGCGCGCTCTCTCTCCCGTCGCCTCTCCCCCTCGCCGGGCCCGTCTCCCGACGGA
GCGTCGGGCGGGCGGTCGGGCCGGCGCGATTCCGTCCGTCCGTCCGCCGAGCGGCCCGTCCCCCTCCGAGACGCG
ACCTCAGATCAGACGTGGCGACCCGCTGAATTTAAGCATATTAGTCAGCGGAGGAGAAGAAACTAACCAGGATTC
CCTCAGTAACGGCGAGTGAACAGGGAAGAGCCCAGCGCCGAATCCCCGCCCCGCGGCGGGGCGCGGACATGTGG

CGTACGGAAGACCCGCTCCCCGGCGCCGCTCGTGGGGGGCCCAAGTCCTTCTGATCGAGGCCCAGCCCGTGGACG
GTGTGAGGCCGGTAGCGGCCCCCGGCGCGCCGGGCCCGGGTCTTCCCGGAGTCGGGTTGCTTGGGAATGCAGCCC
AAAGCGGGTGGTAAACTCCATCTAAGGCTAAATACCGGCACGAGACCGATAGTCAACAAGTACCGTAAGGGAAAG
TTGAAAAGAACTTTGAAGAGAGAGTTCAAGAGGGCGTGAAACCGTTAAGAGGTAAACGGGTGGGGTCCGCGCAGT
CCGCCCGGAGGATTCAACCCGGCGGCGGGTCCGGCCGTGTCGGCGGCCCGGCGGATCTTTCCCGCCCCCCGTTCC
TCCCGACCCCTCCACCCGCCCTCCCTTCCCCCGCCGCCCCTCCTCCTCCTCCCCGGAGGGGGCGGGCTCCGGCGG
GTGCGGGGGTGGGCGGGCGGGGCCGGGGGTGGGGTCGGCGGGGGACCGTCCCCCGACCGGCGACCGGCCGCCGCC
GGGCGCATTTCCACCGCGGCGGTGCGCCGCGACCGGCTCCGGGACGGCTGGGAAGGCCCGGCGGGGAAGGTGGCT
CGGGGGGCCCCGTCCGTCCGTCCGTCCGTCCTCCTCCTCCCCCGTCTCCGCCCCCCGGCCCCGCGTCCTCCCTCG
GGAGGGCGCGCGGGTCGGGGCGGCGGCGGCGGCGGCGGTGGCGGCGGCGGCGGCGGCGGCGGACCGAAACCCCC
CCCGAGTGTTACAGCCCCCCCGGCAGCAGCACTCGCCGAATCCCGGGGCCGAGGGAGCGAGACCCGTCGCCGCGC
TCTCCCCCCTCCCGGCGCCCACCCCCGCGGGAATCCCCCGCGAGGGGGGTCTCCCCCGCGGGGGCGCGCCGGCG
TCTCCTCGTGGGGGGGCCGGGCCACCCCTCCCACGGCGCGACCGCTCTCCCACCCCTCCTCCCCGCGCCCCCGCC
CCGGCGACGGGGGGGGTGCCGCGCGCGGGTCGGGGGGCGGGGCGGACTGTCCCCAGTGCGCCCCGGGCGGGTCGC
GCCGTCGGGCCCGGGGGAGGTTCTCTCGGGGCCACGCGCGCGTCCCCCGAAGAGGGGGACGGCGGAGCGAGCGCA
CGGGGTCGGCGGCGACGTCGGCTACCCACCCGACCCGTCTTGAAACACGGACCAAGGAGTCTAACACGTGCGCGA
GTCGGGGGCTCGCACGAAAGCCGCCGTGGCGCAATGAAGGTGAAGGCCGGCGCGCTCGCCGGCCGAGGTGGGATC
CCGAGGCCTCTCCAGTCCGCCGAGGGCGCACCACCGGCCCGTCTCGCCCGCCGCGCCGGGGAGGTGGAGCACGAG
CGCACGTGTTAGGACCCGAAAGATGGTGAACTATGCCTGGGCAGGGCGAAGCCAGAGGAAACTCTGGTGGAGGTC
CGTAGCGGTCCTGACGTGCAAATCGGTCGTCCGACCTGGGTATAGGGGCGAAAGACTAATCGAACCATCTAGTAG
CTGGTTCCCTCCGAAGTTTCCCTCAGGATAGCTGGCGCTCTCGCAGACCCGACGCACCCCCGCCACGCAGTTTTA
TCCGGTAAAGCGAATGATTAGAGGTCTTGGGGCCGAAACGATCTCAACCTATTCTCAAACTTTAAATGGGTAAGA
AGCCCGGCTCGCTGGCGTGGAGCCGGGCGTGGAATGCGAGTGCCTAGTGGGCCACTTTTGGTAAGCAGAACTGGC
GCTGCGGGATGAACCGAACGCCGGGTTAAGGCGCCCGATGCCGACGCTCATCAGACCCCAGAAAAGGTGTTGGTT
GATATAGACAGCAGGACGGTGGCCATGGAAGTCGGAATCCGCTAAGGAGTGTGTAACAACTCACCTGCCGAATCA
ACTAGCCCTGAAAATGGATGGCGCTGGAGCGTCGGGCCCATACCCGGCCGTCGCCGGCAGTCGAGAGTGGACGGG
AGCGGCGGGGGCGGCGCGCGCGCGCGCGCGTGTGGTGTGCGTCGGAGGGCGGCGGCGGCGGCGGCGGGGGTG
TGGGGTCCTTCCCCCGCCCCCCCCCCCACGCCTCCTCCCCTCCTCCCGCCCACGCCCCGCTCCCCGCCCCCGGAG
CCCCGCGGACGCTACGCCGCGACGAGTAGGAGGGCCGCTGCGGTGAGCCTTGAAGCCTAGGGCGCGGGCCCGGGT
GGAGCCGCCGCAGGTGCAGATCTTGGTGGTAGTAGCAAATATTCAAACGAGAACTTTGAAGGCCGAAGTGGAGAA
GGGTTCCATGTGAACAGCAGTTGAACATGGGTCAGTCGGTCCTGAGAGATGGGCGAGCGCCGTTCCGAAGGGACG
GGCGATGGCCTCCGTTGCCCTCGGCCGATCGAAAGGGAGTCGGGTTCAGATCCCCGAATCCGGAGTGGCGGAGAT
GGGCGCCGCGAGGCGTCCAGTGCGGTAACGCGACCGATCCCGGAGAAGCCGGCGGGAGCCCCGGGGAGAGTTCTC
TTTTCTTTGTGAAGGGCAGGGCGCCCTGGAATGGGTTCGCCCCGAGAGAGGGGCCCGTGCCTTGGAAAGCGTCGC
GGTTCCGGCGGCGTCCGGTGAGCTCTCGCTGGCCCTTGAAAATCCGGGGGAGAGGGTGTAAATCTCGCGCCGGGC
CGTACCCATATCCGCAGCAGGTCTCCAAGGTGAACAGCCTCTGGCATGTTGGAACAATGTAGGTAAGGGAAGTCG
GCAAGCCGGATCCGTAACTTCGGGATAAGGATTGGCTCTAAGGGCTGGGTCGGTCGGGCTGGGGCGCGAAGCGGG
GCTGGGCGCGCGCCGCGGCTGGACGAGGCGCCGCCGCCCCCCCCACGCCCGGGGCACCCCCCTCGCGGCCCTCCC
CCGCCCCACCCCGCGCGCGCCGCTCGCTCCCTCCCCGCCCCGCGCCCTCTCTCTCTCTCTCTCCCCCGCTCCCCG
TCCTCCCCCCTCCCCGGGGGAGCGCCGCGTGGGGGCGGCGGCGGGGGGAGAAGGGTCGGGGCGGCAGGGGCCGGC
GGCGGCCCGCCGCGGGGCCCCGGCGGCGGGGGCACGGTCCCCCGCGAGGGGGGCCCGGGCACCCGGGGGGCCGGC
GGCGGCGGCGACTCTGGACGCGAGCCGGGCCCTTCCCGTGGATCGCCCCAGCTGCGGCGGGCGTCGCGGCCGCCC
CCGGGGAGCCCGGCGGGCGCCGGCGCGCCCCCCCCCCACCCCACGTCTCGTCGCGCGCGCGTCCGCTGGGGGCG
GGGAGCGGTCGGGCGGCGGCGGTCGGCGGGCGGCGGGGCGGGGCGGTTCGTCCCCCCGCCCTACCCCCCCGGCCC
CGTCCGCCCCCCGTTCCCCCCTCCTCCTCGGCGCGCGGCGGCGGCGGCGGCAGGCGGCGGAGGGGCCGCGGGCCG
GTCCCCCCCGCCGGGTCCGCCCCCGGGGCCGCGGTTCCGCGCGGCGCCTCGCCTCGGCCGGCGCCTAGCAGCCGA
CTTAGAACTGGTGCGGACCAGGGGAATCCGACTGTTTAATTAAAACAAAGCATCGCGAAGGCCCGCGGCGGGTGT
TGACGCGATGTGATTTCTGCCCAGTGCTCTGAATGTCAAAGTGAAGAAATTCAATGAAGCGCGGGTAAACGGCGG
GAGTAACTATGACTCTCTTAAGGTAGCCAAATGCCTCGTCATCTAATTAGTGACGCGCATGAATGGATGAACGAG
ATTCCCACTGTCCCTACCTACTATCCAGCGAAACCACAGCCAAGGGAACGGGCTTGGCGGAATCAGCGGGGAAAG
AAGACCCTGTTGAGCTTGACTCTAGTCTGGCACGGTGAAGAGACATGAGAGGTGTAGAATAAGTGGGAGGCCCCC
GGCGCCCCCCCGGTGTCCCCGCGAGGGGCCCGGGGCGGGGTCCGCCGGCCCTGCGGGCCGCCGGTGAAATACCAC
TACTCTGATCGTTTTTTCACTGACCCGGTGAGGCGGGGGGGCGAGCCCCGAGGGGCTCTCGCTTCTGGCGCCAAG
CGCCCGGCCGCGCGCCGGCCGGGCGCGACCCGCTCCGGGGACAGTGCCAGGTGGGGAGTTTGACTGGGGCGGTAC
ACCTGTCAAACGGTAACGCAGGTGTCCTAAGGCGAGCTCAGGGAGGACAGAAACCTCCCGTGGAGCAGAAGGGCA
AAAGCTCGCTTGATCTTGATTTTCAGTACGAATACAGACCGTGAAAGCGGGGCCTCACGATCCTTCTGACCTTTT
GGGTTTTAAGCAGGAGGTGTCAGAAAAGTTACCACAGGGATAACTGGCTTGTGGCGGCCAAGCGTTCATAGCGAC
GTCGCTTTTTGATCCTTCGATGTCGGCTCTTCCTATCATTGTGAAGCAGAATTCACCAAGCGTTGGATTGTTCAC
CCACTAATAGGGAACGTGAGCTGGGTTTAGACCGTCGTGAGACAGGTTAGTTTTACCCTACTGATGATGTGTTGT
TGCCATGGTAATCCTGCTCAGTACGAGAGGAACCGCAGGTTCAGACATTTGGTGTATGTGCTTGGCTGAGGAGCC
AATGGGGCGAAGCTACCATCTGTGGGATTATGACTGAACGCCTCTAAGTCAGAATCCCGCCCAGGCGGAACGATA

CGGCAGCGCCGCGGAGCCTCGGTTGGCCTCGGATAGCCGGTCCCCCGCCTGTCCCCGCCGGCGGGCCGCCCCCCC
CCTCCACGCGCCCCGCGCGCGCGGGAGGGCGCGTGCCCCGCCGCGCGCCGGGACCGGGGTCCGGTGCGGAGTGCC
CTTCGTCCTGGGAAACGGGGCGCGGCCGGAGAGGCGGCCGCCCCCTCGCCCGTCACGCACCGCACGTTCGTGGGG
AACCTGGCGCTAAACCATTCGTAGACGACCTGCTTCTGGTCGGGGTTTCGTACGTAGCAGAGCAGCTCCCTCGC
TGCGATCTATTGAAAGTCAGCCCTCGACACAAGGGTTTGTCCGCGCGCGCGCGCGCGCGCGCGTGCGGGGGGCCC
GGCGGGGCGTGCGCGTCCGGCGCCGTCCGTCCTTCCGTTCGTCTTCCTCCCTCCCGGCCTCTCCCGCCGACCGCG
GGCGTGGTGGTGGGGGTGTGGGGGGGAGGGCGCGCGACCCCGGTCGGCGCGCCCCGCTTCTTCGGTTCCCGCCTC
CTCCCCGTTCACCGCCGGGGCGGCTCGTCCGCTCCGGGCCGGGACGGGGTCCGGGGAGCGTGGTTTGGGAGCCGC
GGAGGCGGCCGCGCCGAGCCGGGCCCGTGGCCCGCCGGTCCCCGTCCCGGGGGTTGGCCGCGCGGGCCCCGGTGG
GGCGGCCACCCGGGGTCCCGGCCCTCGCG

Supplementary Figure S1. The sequence of the human 45S rRNA precursor RNA45S5 (NR_046235.1), plus the 1 kb of 5' sequence on human chromosome 21 (taken from GRCh38 Primary Assembly). The transcription start site (taken as the 5' end of NR_046235.1) is highlighted in pink. Sequences 1 kb 5' of the mature, processed ribosomal RNAs are highlighted in grey. **Sequences of the mature, processed ribosomal RNAs (RNA18S5 (NR_003286.2); RNA5.8S5 (NR_003285.2); RNA28S5 (NR_003287.2), respectively) are underlined and in blue**. Sequences matching the ZTRE segment C-A/C-C-T/A/G-C-C-C/T are highlighted in yellow. Sequences matching the corresponding complementary ZTRE segment A/G-G-G-C/T/A-G-G/T-G are highlighted in green. **Sequence in red and underlined matches a ZTRE segment that overlaps with another ZTRE segments**. Cyan highlighting between two of these segments indicates a space smaller that 30 bases; blue shading indicates a space between 30 and 50 bases. Only regions within 1 kb of the transcription start site or the 5' end of a processed transcript have been mapped for the occurrence of ZTREs. Sequences corresponding to the RNA18S5 and RNA28S5 probes on the Illumina HT12 beadchip array are shaded purple. The regions amplified in RT-qPCR to measure 45S rRNA and 18 s rRNA are shaded red.
