# Supplementary material for: The Zinc Finger Protein ZNF658 Regulates the Transcription of Genes Involved in Zinc Homeostasis and Affects Ribosome Biogenesis through the Zinc Transcriptional Regulatory Element
Source: Mol Cell Biol. 2015 Feb 18;35(6):977–87. doi: 10.1128/MCB.01298-14 (PMC4333095; doi:10.1128/MCB.01298-14)
Supplement: Supplemental material [file MCB.01298-14_zmb999100760so2.pdf]

RPS18

AATAAAAGGAATTTTATCTAATTTGTACTCTGAGCAAATAAAATATGTAATTAACATGAAATCAAAATAC
ACATGGTACTAGCATTTTTTTGTTTTTTCATTGAGTAGTTGGAGAGGGGTGAGCCTTAGAGCACAAAGGT
ACCTTACTTTGAAAAAATTACCTAGTAAGCTGGGCACAGTGGCTCACACGTGTAATCCCAGCACTTTGGG
AGGCCAAGGCGGGTGGATCACCTGAGGTCAGGAGTTTGAGACCAGCCTGGGCAACATGGCAAAACCCCGT
CTCTACTAAAAGTACAAATTAAGATGGGCGTGGTGACAAGGGCCTGTAATCCCAGCTACTCAGGAGGCTG
AGGCAGGAGAATCGCTTGAACCCAGGACGCAGAGGTTGCAGTGAGCTGAGATCATGCCACTACATTCCAG
CTTGGGCGACAGAGCGTGACTTCATCTCAAAAAAAGGAAGAAAAAACTACCTAATAAGAAAGTTTAAGTC
TGTAAGGGTACCCTTCATAACCCATTTGCATTCTGGTTGCTTCCTTTTTTGAAAGAAGGGCAGAGCTACC
TCTCTAGTATGCAGAGGTGTGCAGCCTAAATGAGAAGGTATTCAAGACCCTACTAGTGTCTGACATAATA
AATGTCAACTTCATTGACGCAGTTCTTGGTATAACCAAAAAAGCTTGAGATGGCCATGATTATGTATGAC
TAGCTCTTCTTTAACATTTTTCTTAACATTATTTCTCCTAACATTATTTCAAACAGCTACAAAGATTTAA
CCAAAGGGAGGAGAAAAAAAAACTACCAATTTTACCCTATTATTTAAAGCAGGTAGTTCAAATAAGTAGC
TTCTCTCATAGACAGAAATTAATGTTGGCCGGGCGCGGTGGCTGACGCCTGTAATCCCAGCACTTCGGGA
GGCCGAGGCGGGCGGATCACGAGGTCAAGAGATTGAGACCATTCTAGCCAACATGGTGAAATCTCGTCTC
TACTAAAAATACAAAAATTGCTCCTCCACAGGAGGCCTACACGCCGCCGCTTGTGCTGCCGCCATGTCGC
TAGTGATCCCTGAAAAGTTCCAGCATATTTTGCGAGTACTCAACACCAACATCGATGGGCAGCGGAAAAT
AGCCTTTGCCATCACTGCCATTAAGGGTGTGGGCCGAAGATATGCTCATGTGGTGTTGAGGAAAGCAGAC
ACTGACCTCACCAAGAGGGCGGGAGAACTCACTGATGATGAGGTGGAACGTGTGATCACCGTTATGCAGA
ATCCACGCCAGTACAAGATCCCAGACTGGTTCTTGAACAGACAGAAGGATGTAAAGGATGGAAAATATAG
CCAGGTCCTAGCCAATGGTCTGGACAACAAGCTCCGTGAAGACCTGGAGCGACTGAAGAAGATTCGGGCC
CATAGAGGGCTGCGTCACTTCTGGGGCCTTCGTGTCCGAGGCCAGCACACCAAGACCACTGGCCGCCGTG
GCCGCACCGTGGGTGTGTCCAAGAAGAAATAAGTCTGTAGGCCTTGTCTGTTAATAAATAGTTTATATAC
CTATGAAAAAAATAAAAAATAAAAAAAATAAAAAATAAAAATACAAAAATTAGCTGGGCGTGGTGGCCCG
CGCCTGTAGTCCCAGCTACTTGGGAGGCTGAGGCAGGAAAATCACTTGAATCGGGGAGACGGAGTTTGCA
GTGCGCCCAGGTCACGCCACTGCATTCCAGCCTGGCGACAGAGCTCCATCTCAAAAAAAAAAAAAAGGAA
ACCATTCATAAAAAAAAAAAAACTTATACTTCCAACTTAGTAGACTGCTCGACAGGAATTAAAATTCAGAA
TATTCTCTAGTTAAGCCCCTAAGACAAACTTGACAGTGTTTTCAAAAAAGTGACTATCTGCCACAAAATT
ACCAATTTTGCAATATCCATGGTATGACATATTTTAACTATGATTCCTATGCACAATTTCATTAAAAATA
TCTGACATTCAAAAATGGCTTAAACTTAACAGCACTACTTT

RPS26

TTTTGATCAGCATTTATTAAAATAGAGAACAGAAAGATTAAGTAAAAATAAAGAATAGAACATACCAGCA
CACCTATGACAAGGGTAAGTATCGTTTCATAAAGCTTGTGTTTCAGTTTTGCATGTGCATGTAATCGTGT
TTGTGTGTGTACTGGGTACCAATGTAAAATGTATTCGTGCTATGAATCTGGGTGAAAAACGTTTAAGTCA
TTAGTCCAGGCTATTCCTCCATCCATTTGTTAGGTGACATACAGCATAGTACAGCGGAGCCAGAATACCT
ACTGCACGTCAGTTCTTCAATCTTTTTTTTTAATTGATTTATTTATTTTTGAGACAGAGTTTCGATCTTT
TTACCCAGGCTAGAGTGCAGTGGCGCGATCTCGGCTCACTGGAAACTCCGCCTCCCAGGTTCAAGATTCT
CCTGTCTTAGCCTCCTGAGTACCTGGGATTACAGGCGCCCACCACCAGGCCGGGCTAAATTCTTCAATCT
TTAAAGTGAGGATAATAATAGTAGCTACCTATAGGCTTGCTCAAAGTTAAATGAGTTAATATTTATAAAG
ACGCTTACCACAGGGCCAGGCACTGTGCCAGAGATAAAAAGATGAATGAGAAATAGCCCCAGCCTCAATG
CACTTCATTGTGGGATTTTCTCCATATTGGAAGGAGGGATTCACAAAATGCTTGTTTCCTCTCAGGCCTT
CCTCTTCGTAATCCTACACCTACCTAGTAGAAGGACTGTCAGAGAAATGAGCCCAGGGCGTCCCCCGGTG
GCGCAGCGCCGCCTTCGGTCTAAGCACTTGGGGCAGGGGGGAAGGGGGGGAGGGGGGAGCCTCTTGGCTT
CGCACTACAATTCCCACCAAGCATCTGTCCCTCTCTGGCCTCTAATAACGAGTGCGCCTTGTGGTCCAGC
GTCTGGCAACTTTCACGCCTAGCGTCGCGTATACCGGAAAACTGCATCGTCCAGAATTGCTTCCGAGTCG
GCGCGGCGCAAGGCTTGCTGGGAGACACATAACCTCGATTTTCTTCCGCCATCCGGCTAAATAGTCCCAT
GTGCACTTTGTTCCATGGATAAATAAACACTAGGAACGCATTTCCACCCTAGATTTCAGCAGAAATGCTG
AATGTAAAGGAATATTTGAGTAAAGTGAGTTGCCGTTCTTGAAGCCCGTCTCCTAAGGATTCTCCCGGTG
TCCGCGTAGGGATCTCATGCTATATAGGAGGGCCCTGCCAGGCACCGTCTCCTCTCTCCGGTCCGTGCCT
CCAAGATGGTGAGTCTTCTTGCGTGGTGAGGGTGGGGGTTCGGGTGCAGACTCTGGGATTGTGGGGAAGT
GAGAGCCTGGAGCACGGCTGAGGGGTGGACCGAGTGTACATTTCATTTGCTCTGGGGGTCGGCGGGATTT
GCGGAGAAACAGGAGATCCGAGCGGCGCCTTCCTGGAGGCTGCCGGTGCGGCTTGTGGCCGGAAAGGGAC
TGAGGCTGGGTGAGTTGCGCCGTTTTCCTAACAGTTTTCCCATCCTGTCGCAGACAAAGAAAAGAAGGAA
CAATGGTCGTGCCAAAAAGGGCCGCGGCCACGTGCAGCCTATTCGCTGCACTAACTGTGCCCGATGCGTG
CCCAAGGACAAGGCCATTAAGAAATTCGTCATTCGAAACATAGTGGAGGCCGCAGCAGTCAGGGACATTT
CTGAAGCGAGCGTCTTCGATGGTAAGTGGGTCACCGGCGCGAACTGTGTGAGGATCCCAGTATCTTAAAG
CCTTCGCCCAACTTCGCCCTTTTGGAGGCTCTGTTCTTTGGAGCCTCTCAGGCAATTTCCACGTATTTAA
GGTTGTTACTGGTAGAAGAGAATTCTCTTGTTTGCCGTTTTGATTCTTTTCTGGGCAGAGGGTGACTTTG
TGATAGAGTGCACAGCCTTTACTCTGAGGTAAAGGTTTGCCTGTTTCGGTTATGAGATTGCAAAAACTAG
AAACTTGGTAAATTTGACAATTCTTGTGCTATTGATTATTT

RPS27

CAATCCCTTCACCGGATAGGCATCCTCCCACGTTGTACATCTTCCATCCTCCAGATCCTCGAGCCCTCTC
CTTTTCACCCTTCTCATAGTCCCTGGAGAGCCTGCCTCCTCTACAAACCCTGTTCTGATCAGAAGACAGA
TGGCAGGTGCGGGACATGAACACAGACACTAGGCCCCCCTTGCTAGGCAAGAGCACTGAGCACCATACCT
GGTGCTCACCCCTGACCTGCACCCACTAGAAGACTAGCTGGCTAGCGGGCTCCTCCATCCTCTCACCTCT
GGGTGCCCCATTCCCTCATCAGGTCTGCAGTGCGTGTGCACAAGAGCTCAGCTGAACCCTGGGGGAGGGT
GGCAAAGCATGGAAGATCAGAAGTCTCTAGGCTAGATGCAGTGGCTCACACCTGTAATCGCAGCACTTTG
GAAGCCAAGGCAGGATTGCTTAAGGCCAGGAGTTCAAGACCAGCCTGGACAACACAGGGAGACCCTGTTT
CTACAAAAAATTTAAAAATTTAAAAAATATATAAAAAGAAGTCTCTATATAACATAAGATGTATAAAACC
TTCATGAAACTTTACAGAAAAACATTAAAGAAGACCTAATGTATGGTGAAATATACCATGTTCATGAATA
GAAATAGATAAGTAGAATACTATTCATATTCATGAATAGAAATATAAAGATAAGCCGGTCATGGTGGCTC
ACGCCTGTAATCCCAGCACTTTGGGAGGCCGAGGCTGGCAGATCACGAGGTCAGAAGTTCGAGACCAGCC
TGGGCAACATAGCGAAGCCCCATCTCTACTAAAAATACAAAAATTAGCCAGGCATGGTGGCACACACCTG
TAATCCCAGCTACTCGGGAGGCTGAGGCATAAGAATTGCTTGAACCTGGGAGGCAGAGGTTCCGATGAGC
TGAGATCATGCCACTGCACTCCAGCCTGGGTGACGGAGAAAGATTATATCTCAAAAAATAAAAGAAAGAA
ATACAAAGACAGCAATTTTGCTTTCCGGTGGTGACGACCTACGCACACGAGAACATGCCTCTCGCAAAGG
ATCTCCTTCATCCCTCTCCAGAAGAGGAGAAGAGGAAACACAAGAAGAAACGCCTGGTGCAGAGCCCCAA
TTCCTACTTCATGGATGTGAAATGCCCAGGATGCTATAAAATCACCACGGTCTTTAGCCATGCACAAACA
GTAGTTTTGTGTGTTGGCTGCTCCACTGTCCTCTGCCAGCCTACAGGAGGAAAAGCAAGGCTTACAGAAG
GATGTTCCTTCAGGAGGAAGCAGCACTAAAAGCACTCTGAGTCAAGATGAGTGGGAAACCATCTCAATAA
ACACATTTTGGATAAAAAAAAAAAAAAAAAGACAGCAATTTTCCCTAAGATGATGAATGAATTCAATGAAA
GGTCAACCAAAATATCAAAAGGGTTTGGGGGAAGAGGCTGGCTGACTGATTTTTGGTGAAACAAGACAAA
CTAATTCTGAAATGTATGCAAAAGAACAAAGGGCCAAGAACAGCAAAAATACTCTTGAAAAAGCGTAAGA
GGAAAGGATTTGCCCTACCAGATATTGAGACTTATTATAAAGTTGTAGTAATGAAGACAGTGCGGCATCA
GGATAGACAAACAGTCCAGTGGAAAAGACCCAGGCATACACAGAAACACAGGTGGCGGTACAGGTCTGTG
GGGAAAGGTGGACTAGTTAATCAATGGAGCTATGGAGGAAAAATCAGAGCCCTATCTTATATACAAATAT
AAATTCCAAATATACTAAGGACTTAAATATAAAAAGAAAAGCTTCCAGAAATCAGTCCAGGAACTCATCT
TCAGTCACTTACTCATTCATTCAACAATTCACAGGCACTGATTATGTTGCGACAAGCACTGTCTTACTAC
CAGGCTGTAAAGCTGAAGCAGGGCGCCGTGGCTCACGTCTGTAATCCCAGCACTTTGGAAGGCCGAGGCA
GAAGGATTGCTTGAGCCCAGGAGGTTGAGACCAGCCTGGAC

RPS29

AGGTCTCTGGGCCCACTGCATCTCTCCTCCCACAGCTGGACCTGAGAGTCAGGGAGCACCTCTTGTATGC
CAGCCATGCTGGGTCTGGGACCCCCTCACCCTCAGGGCCCTCAGGTGGTTCCCTCAGGCTGCAGCGGACA
TAGAGATCAGTTCTGTGTGTTCCCTAAGCTGACGTGGAAAGTTGGGAAGCACACGCTTGAGCCCTTGAAC
TTCCTCTTTCTCAGAGCCTAACAGTAGAGCCGGGGTGGGTTTCTTGCCAGAACCACCTCCTCGCAGAATC
CCAGGAGACACCAGCCAGGTCCTTTCACACCCCAAACAGTGTGAATGCGCATGTTTACAGCAGCACAATT
CGCAATTGCAAAAATGTGGAACCAATCCAAATGCCCATCAATCAAAAAGTGGATAAAGAAACTATGGTAT
ATATACATGATAGAATACTACTCGGCCCTAAAAAGGAATGAATTAATGGCAATTGCAGTAAACCGATGAG
ATTGGAGACAATGATTCTAAGTGAAGTAACTCAAGAATGGAAAACTAAATATTGTATGTTCTCACTCATA
AGAGGGAGCTAAGCTATGAGGATGCAAAGGCATAAGAATGACACAATAGACTTTGGGGACTTAGAGGGAA
AGGGTGGGAAGGGGGTGAGAAATAAAAGACTACAAATGGGGTGCATTGTTTACTGCTCGGGTGATGGATG
CACCAAAATTTCACAGATCCCCACTAAAGAACTTGCTCATGTAACTGAACACCACCTGTTCCCCAATAAC
CTATGGAGATAAAAAGATTAAAAACAGAAACAAAAACCGAACAGTGTGAACAGTGCACCCTGAGGTCTCC
CTGGGGTCCCTTGTACCCCTCAGGAGCCTGTGGATGACCCTGTGCAAACAGTTGGGTGGAAGACTGTGTC
TTCCTGTGTGTTTGGATGAGTACCAGAACTTTCTGCGTCTGTTTTTCACCCTACCCAATGCAGTGCTTAC
CTAAAAAATGCGTTGAATAGAGCTTCTTGCTTTTACCTCGTTGCACTCCTGAGAGCAAGATGGGTCACCA
GCAGCTGTACTGGAGCCACCCGCGAAAATTCGGCCAGGGTTCTCGCTCTTGTTGCGTGTGTTCAAACAAC
CGGCACGGTCTGATCCGGAAATATGGCCTCAATATGTGCCGCCAGTGTTTCCGTCAGTACGCGAAGGATA
TCGGTTTCATTAAGTTGGACTAAATGATCTTCCTTCAAAGGATTATCCAAGGCATATACTCAATGAAAAA
CCATGATAGTTCTTTGTACATAAAATAAACATTTGAAAAAACAACAACAAAAAATGCGTTGAATAAACGA
AGGCCAAAATGTCAGTTTCCAAAAAGAACCTGAGGCGGTTCTTTGGTCAGAGCAGCCCTGTTAGGGCTGA
GGGCCTCGGTTCCACCTCCTCCATGTTTTCCTCTCTCTTCCCACTGTGAGCTCTCTCAGACCTGGCACTG
TGGCCGCTCCCAGCTCCTCGCACCCAGCATGGGGCAGGTGCTGAATGTCTGAGGAGCTTAATTTCCCCTC
CCTCTTGCCTTCTGCCTGGGGTGAGGGCTCAGTTGCTCTGAAGCTCATGACCAGGCTTAATAGTCAGTTG
AGCTATAGGCCTGCAGATCGAGTATCCCTTTAAGTTTGTTTTTAAAATATTAAATTTACCAAGAAACAGT
AAATATTTAAAATTTTTGTTGGGTTTTGCTCTTTTACTGATTTCTACTAGATTTTTTGGCTCCTTTTAAA
GGTATAATTTATATGCTAGGAGTGGTGTCTCATGCCTGTAATCCTAGCACTTTGGGAGACTGAGGCAAGA
GGATCGCTTACAGCCAGGAATTCAGCCCCAGCCTGGGCAATGTAGCAAGACCCTGTCTCTACAATTAAAA
AAAAAAAAACAGAAGGAAAAAAGGTATAGTTTGTGTTCAGTAACATTCACACCTTTTAATATGCAATTCT
GAGTTTTAACAAATGCATAGTCATGGAACTACTACTGCAAT

RPL6

AAAATGTCAGTGAAGAATTCCATATCCAGAAAACCTTTGCTTCAAATATGGAAAAATTCAGACTTTTCCA
GATAAATAAAATCTGAGGGAATTAATTGCTAATATACTTTCACTACAAGAAATTCTACAGGAAACCCTTT
AGGCTGAAATGAAAGGAAACTAGACATTAACCCAAAGGCATATGAGAAAATGAAAAACCTCCATAAAGGA
ACTACATAGGCAAATGTAAAAGTCAGTATTATTGTATTTTTGTTTTGTGTCTGCATTTTAAAATTTCCTC
TGTGATTTAAAAATAGCACATGAAATAATAATTACAAACTGTGTTTATTGGCACACAATATATAAGGATG
TAATTTGTGACAATAACAACATAAATGGGAATGGAGCTTTATAGGAGTAGACTTTTTATATGCCATTGAA
TCTAAGTTGGTATAAATTCAAATTATATTGTTATAACTTACTAATGTAAATTAAAATACTAGAGAAACCA
CTAAGAAAATATCTAAAATTTATATGCGAAATTAAGTAAGAATGGTATACTACAAAAAAGAAATTAAACA
CAGAAAAAGCAATAATGAAGGAAATTAGTAATAAAAAGGTATAAGCCATAGAAAACAAATCATAAAATGG
CAGAAGTAAGTGCTTTCTTATCTGTAATTACTATGAAAGTAAATAAATTAAGCATTACAATCAAAAGGCA
AATATTAGCAGAATGGATTTTAAAAACCGTAGTTTAATTACGTTATCTACAAGAAATTCACATTAGATGC
AAATAAACAAATAGGTTAAAAGTGAAAGGGTAGAGAAAAGATATTTCATACAAATGTTAACTAAATGACA
GCTGGGATGGCTATGCTAATACATAATACAGGGTTTGAGTAAAATAGCTGCTGAAAGTGACAGAGAAGAA
CACTAATATTGGTAAAGGGTCCTTTCATCAAGAAGATATAACAATTGTAAACATATATGTCACACAGAAA
AGAGTCTAAAAAAGACACTGCTCTTTCCCATCTTGCAAGATGGCAGGTGAAAAAGTTGAGAAGCCAGATA
CTAAAGAGAAGAAACCCGAAGCCAAGAAGGTTGATGCTGGTGGCAAGGTGAAAAAGGGTAACCTCAAAGC
TAAAAAGCCCAAGAAGGGGAAGCCCCATTGCAGCCGCAACCCTGTCCTTGTCAGAGGAATTGGCAGGTAT
TCCCGATCTGCCATGTATTCCAGAAAGGCCATGTAGAAGAGGAAGTACTCAGCCGCTAAATCCAAGGTTG
AAAAGAAAAAGAAGGAGAAGGTTCTTGCAGCTGTTACAAAACCAGTTGGTGGTGACAAGAACGGCGGTAC
CCGGGTGGTTAAACTTCGCAAAATGCCTAGATAGTATCCTACTGAAGATGTGCCTCGAAAGCTGTTGAGC
CACGGCAAAAAACCCTTCAGTCAGCACGTGAGAAAACTGCGAGCCAGCATTACCCCCGGGACCATTCTGA
TCATCCTCACTGGACGCCACAGGGGCAAGAGGGTGGTTTTCCTGAAGCAGCTGGCTAGTGGCTTATTACT
TGTGACTGGACCTCTGGTCCTCAATCGAGTTCCTCTACGAAGAACACACCAGAAATTTGTCATTGCCACC
TCAACCAAAATCGATATCAGCAATGTAAAAATCCCAAAACATCTTACTGATGCTTACTTCAAGAAGAAGA
AGCTGCGGAAGCCCAGACACCAGGAAGGTGAGATCTTCGACACAGAAAAAGAGAAATATGAGATTACGGA
GCAGCGCAAGATTGATCAGAAAGCTGTGGACTCACAAATTTTACCAAAAATCAAAGCTATTCCTCAGCTC
CAGGGCTACCTGCGATCTGTGTTTGCTCTGACGAATGGAATTTATCCTCACAAATTGGTGTTCTAAATGT
CTTAAGAACCTAATTAAATAGCTGACTACAAAAAAAAAAAAAAAAAAAAAAAAGACACTGACAGGATTGAG
GGGGAAGTAGACAGTTTCACAGTAATACCTGGAGACCTCAA

RPL9

CCAGTCCCAACCTGTAACTCACATTCCCCCTCCCTTTCTTATTAGAGAAAATATTCACAATATCCAACTG
AGTCAGCTAAGATTGTGCAGTCCTACCCCAGCCCATGTTGGAATGACACAGAGGCAGGGGCTGTGTTAGG
GATAAGAACCCCTGCTCCACCCCGTTTGGTGTGCTCTTGCAATCATGACTAATGCAAGCAGCATACTTGC
AGAAGCAAATTGTCTTGCTGAGAAAACTTTTTTGCCTGAGTGCTGCTTCTTCCTCACAGCACCGATCATT
TGTTTCTAACAATCTCGCTAAAAGCAGCCTAGAAAGCAGCCACTTATGCAGAAAGAGTAATAATTTAGGC
TCTACAAGTCATATAAAAAATGAAGTTTCATTTGTTTACTGGCTAATTTACTTCCTGGGAGATATTTTTC
ATTCTAAAACAGTGATTCCCTACCAAGAGTTCACAGATGCCAAGAAGTCCATAAAAGGAGTAATGGAATT
GCCAAATTATGTTAAATACTTCAAAAGGACTCAAAGCCATATACTAGCTCCCAATAGGCCTGCATAAGTT
ATTAGAACAAGCTGCTTTGCATTTTGGGGTGATCAGAACCAGTAACTAGATGGCAATCACGTCTGTTACT
GAAGATGGAAAAACTATACTTAAGTTGGTATAACAATCTTTCATAACATGGCTTCATAGAAAAGAAGTAT
AAAAAGGATTCCTTGAGGTTGAAAAAGAGTGCTCTTTTCCCTTCATTATTTAAGATTAGGACAAATTTAT
AAAACAGGAAAAAAAAATAGCACAAATCCCTTGGCAAACAGAGTAAAACATCTACTCTGTTTTGCTTTTT
TTCACTTCTTACACTCTCTTTCATAGGAAGTCAATTTACAGACTTCCATCAAGCCCTTAGAGACCTTTTT
GTACTATCCATGACAAGCTCTTGATGTTATCTCTGCACTTTTGACAAATTCTTAGCAGTTAACTTACAAG
GCAGTTAAGATTTTTGTTCGCTTTCTTTGCTGCGTCTACTGCGAGAATGAAGACTATTCTCAGCAATCAG
ACTGTCGACATTCCAGAAAATGTCGACATTACTCTGAAGGGACGCACAGTTATCGTGAAGGGCCCCAGAG
GAACCCTGCGGAGGGACTTCAATCACATCAATGTAGAACTCAGCCTTCTTGGAAAGAAAAAAAAGAGGCT
CCGGGTTGACAAATGGTGGGGTAACAGAAAGGAACTGGCTACCGTTCGGACTATTTGTAGTCATGTACAG
AACATGATCAAGGGTGTTACACTGGGCTTCCGTTACAAGATGAGGTCTGTGTATGCTCACTTCCCCATCA
ACGTTGTTATCCAGGAGAATGGGTCTCTTGTTGAAATCCGAAATTTCTTGGGTGAAAAATACATCCGCAG
GGTTCGGATGAGACCAGGTGTTGCTTGTTCAGTATCTCAAGCCCAGAAAGATGAATTAATCCTTGAAGGA
AATGACATTGAGCTTGTTTCAAATTCAGCGGCTTTGATTCAGCAAGCCACAACAGTTAAAAACAAGGATA
TCAGGAAATTTTTGGATGGTATCTATGTCTCTGAAAAAGGAACTGTTCAGCAGGCTGATGAATAAGATCT
AAGAGTTACCTGGCTACAGAAAGAAGATGCCAGATGACACTTAAGACCTACTTGTGATATTTAAATGATG
CAATAAAAGACCTATTGATTTGGAAAAAAAAAAAAAAAGATTTTTGTTCAAGCACAATATAGCTAGACTAG
TGTCATATACTCAATGAAACAAATATTTACCAAGCATTTATTGAGTGGAAGATAAAAAGCACAAAGCATA
ATTATAAAATATTTTCCCCTGCCACCATAAAGAAATTGAACAGGCTTACAGAATATAGCACAAGACAACA
TGACCAAAGCAAAAATAGTAAGGACTAAAGAGGGGAGGAAGGGGAAATATCAACATGAACTGAATATGAC
CCAGAAGAGCCTTCATGGATGGTCAGACATGTAAAGACAAA

RPL17

CTCGTGATCCGCCCGCCTCGGCCTCCCAAAGTGCTGGGATTACAGGCGTGAGCCACCGCGCCCGGCCCAT
TGTCTGTTACTCTTGTCACCATGCAACATAAAAGAGGAACCCGGAGGAGAAGACAAAGAGTTCTGTTTAA
GGGCATGTTAGGTTTCAAATAACCTCAGGTCTTCAGGGACCTCCCAGAAAGGATGTCCAGGGAGTAGTTT
GCTATCAGGGTCTAGCTCAGGAGACAGATGTGGCGCTGGGGTATTAATCTGGGAGTTAGCATGCAGTTGA
AAGCTCATGTGAGGGGAGGGAATTACCGAGGAAACACCATGGAATCCAGCATCAGCAATGGAAGTGGAGT
CGAGGGGAGAGACTTAGGGCTGAGGTCTCCTACACTGAGGTCAAGGAAGCAAGTGAGCCTAGCCTGGAGT
CTACTCTCCTAGAGTTTGAGCTGCCTAGAAGGGTGGTGCCATGAAATAGAAGCCCTAACAAAAGCTAAGT
GCATCACTGACTATGAGGGAATAACCAGAGAGATGACCTGGGGAAGGAAAAAAGGGAAGAAAATGCCAAA
AGGTTTTCTCCTCCAGATGAAGATCTAGAAGCAGCGGGGAAAACGAGGATATTGAACCAGACCCTTCAGT
TAGCGCATTTTTCACACAGGTTATAATACAATTGATTGGATGACATTCCCATCCTGAGAATTTCCTGATT
ACCATAATTACGTCTCATCCTTTCATCTTTTTAATTGGCCATTTACAAACCACTTTCAGGAGTACAGGGC
CACATAATCAAACCTAACTAATGTTGACGAATAAATAAAATGTTTCTTTCATATCTAATAATACGTAGCC
TGTCCTAGAACTACAACCCTGTTTTTAATTGACTGACATTTAAAACCCTGAATTTTTATTTCTGTATACA
AAAAAGAGAACTTTGAGACAGTCCCTAAGTAGGCAATTCGTTTTCACACGTTCTTAAAACATATTCTACA
CTCCCTTCGCTTTCCGCCTTCCTGCCTCCTCAGATCTCGTTTCTTCGGCTACGAATCTCGCGAGAAGTCA
AGTTCTCATGAGTTCTCCCAAAATCCACCGCTCTTCCTCTTTCCCTAAGCAGCCTGAGGTGAGTGTTTCT
CCTGCGTTGCTCCGAGGGCCCAATCCTCCTGCCATCGCCGCCATCCTGGCTTCGGGGGCGCCGGCCTCCA
GGCCCCCGGGAGGAGAACTCCTAGGGCTACTAAATCCTCGCTGGAGGCGGTGGCTTCTTATGCGGGAGGA
CGTGGCGGAGGGCCTGACTTTGGGAGCCGGGGTCAGTCGGCCTCTGAGGTCCGCAGAGGGACGTGATGGG
CGGGAATGGGGACTACCGGGCTCCTCCACTGGTGGGGGCGCCGGCCCGCCGTGGGGTGCGGGCCGCCTGG
GGTCCGTGCGGACTCCGGAGGTCCGGTGTCTAGTGGTGAGTGGTGGCCGCAACGAGGAAAAGTTTTGGG
GGAAAGAAAAGTCGGGTGGAGGCGTAACACGTTACTACAAGAGTGTTGCGTACAGGAGGGCTCTTAAAGT
GGGTCATAGCCCGAAGGTGTTGAGAGAGACGGCACTCACTACCTGCAGCCCTGACAGCAAAGGGGTTTCT
GTAGAGCGGGAGGGAGGAGGTGTAGAGGGTTACGGTTGAGTTGTGCCCTGCGGATGCGTCGAGTCATTTT
ACGCCTGGAAGATCCAGCATTGGATTGAAACAGGCTGTATTTTCTTCCAAAGGGTTGACTGGATTGGTGA
GGCCCGTGTGGCTACTTCTGTGGAAGCAGTGCTGTAGTTACTGGAAGATAAAAGGGAAAGCAAGCCCTTG
GTGGGGGAAAGTATGGCTGCGATGATGGCATTTCTTAGGACACCTTTGGATTAATAATGAAAACAACTAC
TCTCTGAGCAGCTGTTCGAATCATCTGATATTTATACTGAATGAGTTACTGTAAGTACGTATTGACAGAA
TTACACTGTACTTTCCTCTAGGTGATCTGTGAAAATGGTTCGCTATTCACTTGACCCGGAGAACCCCACG
AAAT

RPL21

CCCCCTGAATCTAAAATCAAAGTTGAAAAACAATGAAATAAAATACAAATGTTAATGGACTGTTTATATT
ATTTGTAAGGCTTCCCGTCATCAATAGGCTATAAGTAAAGTTTTGTGGGAATCAAATTTATATGTGTATT
TTTGACTGCTAGTTGGGGTCAGTACCCCTAACCCTTGCATTCTTTGAGGGTCAGCTCTACTACTATATAG
TATACTGGAAATTTGTTAAGAGTCTAAATCTTAAATGTTCTCGTCACAAAAATATTAACCAGGTGAGAAG
ACAATTATGTTATTTAGCTTAATCTTGATCATCATTTTACAATTTATATGTATGCCAAAACATCACATTG
TATACCGTAAATATATACAATTTTGAACTGTTGATTATATCACTAAAGCAGGGGAAAAAACAAAGAACTT
CATATAAGACATACGAGTCTTAGATCATCACAAATCATGAGAAACCTGAAAGAAAACATAATTAGTGTGG
TAAATCCAAAAACAGAAAATAATGTTCTATATGCTTGTCAAGAGCAATGCATTTTTTAAATAAGAGAATT
TATCATCTAGACAAAATACATCATGGAATTTGTAGATTCCCCTTGTTTCCTTTGATTATAATTATACGAT
CATTTCAGGACACAAATTTATCTTTGATTATCTTCATCAGAACATGATGTCACTATTTCTGGCAGTAAAC
ATAAACATTTTTAACAAATAAGGGTAAATATTTTCTAGCCTACTTGCTTTGCATTACCTAGAATTTGGGA
AATCAAAGCTGGCATTTTCTACCTATTCTACAGTTTCTTTTATCTTTAGTCCAAAAAAGTAGATTATAGG
TTTACATTATTTAGGTAATCACAGAGAGTATTAACTTTTATAGCTGCAGAGAAAACCAATTCAAATTTCA
AAACTACTATGCCATTTCCCTACATTAGGTTACTCTCCTCTGCAAATCTGACAAGTTGGCATCCAAGCAA
AATTTAAATGATCACCTTGCCCTTTCGGCCGGAACCGCCATCTTCCAGTAATTCGCCAAAATGACGAACA
CAAAGGGAAAGAGGAGAGGCACCCGATATATGTTCTCTAGGCCTTTTAGAAAACATGGAGTTGTTCCTTT
GGCCACATATATGCGAATCTATAAGAAAGGTGATATTGTAGACATCAAGGGAATGGGTACTGTTCAAAAA
GGAATGCCCCACAAGTGTTACCATGGCAAAACTGGAAGAGTCTACAATGTTACCCAGCATGCTGTTGGCA
TTGTTGTAAACAAACAAGTTAAGGGCAAGATTCTTGCCAAGAGAATTAATGTGCGTATTGAGCACATTAA
GCACTCTAAGAGCCGAGATAGCTTCCTGAAACGTGTGAAGGAAAATGATCAGAAAAAGAAAGAAGCCAAA
GAGAAAGGTACCTGGGTTCAACTAAAGCGCCAGCCTACTCCACCCAGAGAAGCACACTTTGTGAGAACCA
ATGGGAAGGAGCCTGAGCTGCTGGAACCTATTCCCTATGAATTCATGGCATAATAGGTGTTAAAAAAAAA
AAATAAAGGACCTCTGGGCTACAAAAAATAAATAAATAAATAAATAAATAAATAAATAAATAAATGATCA
CCTTAAGTATCAGTTTCTTTTGTAAATGAAACAAAATCACAGAAGATGAGTAGTGACTCAGCAAAGATGC
AATATTGTCTTATTAAGGGTAATTTTTCTTGATTCATGGTCTTCCACAATTATCATGACCTACTTAGGAA
AATTCTTGCAAGATGGTTGTAAACATGTACTGAGGACAGGTTTATTTGCCTCAAATTATATATATAAATT
TCTTCAGTCCTATGTCAAACAATGGTTTTAACCTTTCCCTGCAGGTAGGGTCACATGACTCCCAGTGCAG
ACCAAGCCACAGCCAAATGTTTAAAACTAATCTTGCCCCTTGGGCAAAGGACTTGAAGAACACAAGTCC
ATAATCACAGGGTAAGTATCAGTCCAGTTTTGCTGCCTGTC

RPL23A

AAAACTTTATGATTTATAAATATAAAGCAAAACTTCATGGTTTAGAAACAAAGGATTGTGCTTTTCCATA
TCAGTGGAAAATCAGAACATCAACAGTTGGAAGAACTCATAAGATCTGAGTAATTAAAGATAAGATTTGA
GGAACTCACAAGATTCTTTACTCAGGAACAGGATGTAACCCATGCATCAAGAGATCTTTTCCGGGGGCTA
ATTGGGACTAATGTGACTGGTAGGTTATCAGCAGAGACCACGTCTGTCTTGTTCAACCTGTGTCCCAGGT
CCTAGCACCATGCCTGCTACCATTTAAGTGTTCATGTGAAGTTTGTTGTTGAGAAACTAATTACCTAAAA
GTTAGAACTTGACCTTTGCATTTTATCGAGGAGGCTATGAGACTCAGAGGGTTACACAGCTAGTGAGTGG
CAGAGCCTGGACTTGAACTCAGTTGTGCTGAAGCCAGAATGTGTTCCTTCCTGAGCATTCAAGGACAGGG
AAGGTGAAACTCCAGCTAGTGGGCAGCCCGTGTGCTTGTTCTAGGGAGACTGCAGGCCGATTCCTGGGAG
CTGGGTCTTCATGGTGACACAGTAAGAACAACTCACCTATTGGCTGGGCGCAGTGGCTCACGCCTGTAAT
CCCAGCACTTTGGGAGGCCGAGGTGGTTGGATCACTTGAGGTCAGGAGTTGGAGACCAGCCTGGCCAATA
TGGTGAAACCCTGTCTCTACTAAAAATACAAAAATTAGCTGGGTGTGGTGGCTCATGCCTGTAATCTCAG
CTGCTCGGGAGGCTGAGGTAGGATAATCGCTTGAACCTGGTGGGGCGGAGGTTTCAGTGAGCCGAGATTG
GCACCATTGCCCTCCAGCCTGGGTGACAAAGTGAGACTCTGTCTCAAAAAACAAAGCAAAGCAAAACAAA
ACAAAACGAAACAGAACTACTCACCTATGTTGTGGATCAACTGTGTCCCCACTGTACCAAATCCTTATTT
ATTTAAAAAAATTTGTTTTGGACCCTTTTCACAAGATGGTGCCGAAAGTGAAGAAGGAAGCTCCTGCCCC
TCCTAAAGCCGAAGCCAAAGCGAAGGCTTTAAAGGCCAAGAAGGCAGTGTTGAAAGGTGTCCACAGCCAC
AAAAAGAAGATCCACACGTCACCCACCTTCCGGCGGCCGAAGACACTGCGACTCCGGAGACAGCCCAAAT
ATCCTCGGAAGAGCGCTCCCAGGAGAAACAAGCTTGACCACTATGCTATCATCAAGTTTCCGCTGACCAC
TGAGTCTGCCATGAAGAAGATAGAAGACAACAACACACTTGTGTTCATTGTGGATGTTAAAGCCAACAAG
CACCAGATCAAACAGGCTGTGAAGAAGCTCTATGACATTGATGTGGCCAAGGTCAACACCCTGATTCGGC
CTGATGGAGAGAAGAAGGTATATGTTCGACTGACTCCTGATTACGATGCTTTGGATGTTGCCAACAAAAT
TGGGATTATCTAAACTGAGTGCAGCTGCCTAATTCTGAATATATATATATATATATCTTTTCACCATATACA
TGCCTCTCTGTCAATTTCTGGTTGGGCTGGGAGGCCACACACAGACACTGACATGACAGGGCTTGGGCAA
GACTCCTGTTCTACTTATCCTTTTGAAATATTCACCCTGCCACTCCACCATGTGTGATCACTCCAGAGAT
CTTTGTGACTAGAGTTAGTGTCCTAGGAAAACCAGAACTCAGAACTTGCCTCCATGGTTGAGGGTAACAA
GAAGCTGTACAAGAACCCCTTTTATCCCTGGAAGAGGCTGTGTATGAAACCAATGCCCAGGGTTTGAAGG
GCGTTAGCATCCATTTCAGGGGAGTGTGGATTGGCTGGCTTTCTGGTAGCATTTTGTCCTCACACACCCA
TCTACTGTGTCCAACCGGTCTGTCTGCTTCCCTCACCCCTTGCCCAATAAAGGACAAGGACTTCAGAGGA
AAAAAAAAATTGTTTTAATTATTACATTTATAGCATCCCAG

RPL26

AAAAAAAAGTTATTTTCTTTTTTTGACCCAAGTTGACTCTTTGCAATATCCCCCCATTAACTCTAGTACT
GCCCCTAGAGTGAGAGAGAGCAGTATAGCAGAATGACAATGATCACAGCTCTGGAGCCAGATGGCCTGAG
TTCAAATCCAAGTTCTTCCACTTCTTTGCTGGGCGCCTGTGCCTCATTGTCCCCACCAGAAAAATGTTAA
TTCTATTGGTGCCTACTTGATAGGGTGGTCATGAGGAAAAAATGGGTTTATATGTGTAAAGAACTTAAAC
AATGTATGGGACTTGGTAGGCACACAATTAATGCTAGCTATTGGCAACATTATTTCATCTCTTCATATAC
CTTTTTTTTTTTGAGACAGAGTCTTGCTCTGTCGCCAGGCTGGAGGCTGAAGTGCAGTGGCGCGATCTCA
GTTCACTGCAATCTCTGCCTCCTGGGTTCAAGCAATTCTCCTGCCTCAGCCTCCCAAGTACCTGGGACTA
TAGGCACATGCCACCACACCCAGCTAATTTTTGTATTTTTAGTAGAGACAGGGTTTCACCATGTTGGCCA
GGATGGTCTCAATTTCTTGACCTCATGATCCACCTGCCTCAGTCTCCCAAAGTCACATACCCTTTCTTTA
AAAAAAAAAAAAATGCTATTTGAGGCACTGCACTGAATGTATTCTGAAGCTCTGTTCAGTGAATGCCATG
ATTGTTAAGTAATGTCTGCCACTGGTAGAAGATAAAATGATGTCACATATGTCACATACTTGCCATCTGT
CATCCAATCCTGTTTTCTCATGACAGTTTTAAAAATATTAAATAAAATAGCAAAATTTCCTGTCTTAGCC
TCTCATCCAGGCTAAAGCACCTAGTAGAAATACTGAAGTCTGTAAATTCTGGTTGATATCAAAAGATGAC
CCCTCAAGGCATCCTTGAAGTGGCCTTAAAATTAGAGCAAGAAAGAGCATTTTATTTTTTCATTTATTAC
CTTTTATTAGATACCTCTGGCTCTTCCCTTTTGCGGCTATCACCGAAGCAGGAGTGGCCAAAATGAAGTT
TAATCCCTTTGTGACTTCCGACCGAAGCAAGAATCGCAAAAGGCATTTCAATGCACCTTCCCACATTCGA
AGGAAGACTATGTCTTCCCCTCTTTCCAAAGAGCTGAGACAGAAGTACAACGTGTGATCCATGCCCATCC
GAAAGGATGATGAAGTTCAGGTTGTACGTGGACACTATAAAGGTCAGCAAATTGGCAAAGTAGTCCAGGT
TTACAGGAAGAAATATGTTATCTACATTGAACGGGTGCAGCGGGAAAAGGCTAATGGCACAACTGTCCAC
GTAGGCATTCACCCCAGCAAGGTGGTTATCACTAGGCTAAAACTGGACAAAGACTGTGAAAAGATCCTTG
AACGGAAAGCCAAATCTTGCCAAGTAGGAAAGGAAAAGGGCAAATACAAGGAAGAAACAATTGAGAAGAT
GCAGGAATAAAGTAATCTTATATACAAGCTTCGATTAAAACTTGAAACAAAGAAAAAAAAATACCTGTGA
ATACCCAAGAGTGCCATAATTATTTGGAATCTCTCTAAAAACACTGATTTTTCACAACTTAATAATGAAA
TTAGTATTCTCCTACCACCCATTGCACACACATTGCTGTTGGTGTGTAGTGTGTTGCTGTGGAGAGAACA
TTAGATGGGGAGTTAGGGAGTGTTATGGCCCGAATTATGTCTCACCAAATTTCATATGTTGAGGCCCTAA
CCCCTAGTCAGACTCTATTTAGAGACAGGGTCTTTAAGGAGGTAATTAAGCTTGAATGAGGTGATAAGGA
TGGCACTGTTCTTCAAAAGAACTGAATTCCTTATCGAAGGGGAAGAAGCCTCATTAGGGCATGCATGCAG
AGGAAAGACCAGGTGAGAACACAGCAATAAGGCGGGCATTTGCAAGCCAAGGAGAAAGCCTCACCAGAAA
CCAGCCCTGCCGACACCTTGATCTTGGACTTCTAGCCTCCA

RPL31

TTTCTGCAGGAAGAAAACTTTTCCTATAATGGATTTCTAACATATTTTTAATGAAGACGTGCCTAGACTC
TGTGGCTTTGGAAACTCAAGATATAATTAAAGGTATGAAATGTAAGGAAATGCCCTAATCTTTATACAGC
ATTTACTCTAAGTTTTTACTGTATTTTGTTCATCCTTTTACTTGTTAAAAATATGCATATTGAGGCTGAG
TGCAGCGGCTCATGCCTGTAATCCCAGTACTTTGGGAGGCCGAGGCAGGCGGATCACCTGAGGTCAAGAG
ATCGAGACCAGCCTGGCCAAGATGGTGAAATCCTGTCTCTACTAAAAATACAAAAATTAGCTGGCCGTGG
TGGCGTGTGCCTATAATCCTAGCTACTTGGGAGGCTGAGGCAGAAAAGTTGCTTGATCCCGGGAGGTGGA
GGTTGCAGTGAGCCGAGATCACGCCACTGCACTCCAGCCTAGGCAACAGACGGTGACTCCATCTCAAAAA
AAAAAAAAAAAAAAAATATATATATATATATATATATATATATCTTATTTATTGATGTCTTGTTCCCGAAAA
GACCATGGATGGAATGGCCCTGCTAGAGATTTCATGATCTAAATTGCTATTACAAAATTTAACAATCAAG
TCCTATTAAGTATTCACTTGCAATAATAAAATTATCTCTCCTCTACAAAAGATTCACTGAGTAGGCCAGG
CACAGTGGCTCATGCCTGTACCCAGCACTTCGGGAGGCCGAGGTGGGTGGATCACCTGAGGTCAGGAGTT
CGAGACCAACCTGGCCAACATGGTGAAACCCCGTCTCTATTAAAAACACAAAAATTAGCCGGGTGTGGTG
GCACATGCCTGTAATCCCAGCTACTCAGGAGGCTGAGGCAGGAGAATCACTTGATCCCAGGAGGTGGAGG
TTGCAGTGAGCTGAGATTGCACCATTGCACTCCAGCCTGGGCGACAGAGCGACAGAGCGAGACTCCATCT
CAAAAAAACAAAAAAACAAAACTTCCTTTCCAACTTAGACCCGGCAGAATGTCTCCCGCAAAGAAGGGTGG
CGAGAAGAAAAAGGGCCCTTCTGCCATCAACGAGGTGGTGACCCAAGAACATACCATCAACATTCACAAG
CGCATCCATGGAGAGGGCTTCAAGAAGCGTGCTCCTCGGGCACTCAAAGAGATTCGGAAATTTGCCATGA
AGGAGATGGGAACTCCAGATGTACGCATTGATACCAGGCTCAACAAAGCTGTCTGGGCCAAAGGAATAAG
GAATGTCCTATAGGGAATCCATGTGCGGTTGTCCAGAAAACGTAATGAGGGTGAAGATTCACCAAATAAG
CTCTATACTTTGGTTACCTATGTACCTGTTACCACTTTCAAAAATCTACAGACAGTCAATGTGGACAAGA
ACTAATCTCTGATCATCAAATACATCAAATAAAGTTATAAAATAGCAAAAAAAAAAAAAAGATTCACTGA
GTAGTGGGAATTAGCAATGATTGATATGTTTACATCTTTGCCACAGTTAATGAGCACATTTTATCTTGAA
ACACAGCTTTTTAAACTTGTGTTTATGTCCCTCCTACTCCAAGTACCCTGGCAGATTTAATTTGGGCTGA
GATTGAACTCCCCTCAGTTGGAATCTGTGGTTACTAACCACAATTAAATTCCTAAAGAAATGGAGATTCC
CACTAGAAGTATCTGTCTAAATTATTCTCTCCCCAGTGACCATTGTCGAGACTAGGTTTAGACAAACTAA
ACATGGCTTCAAATCAACTCCAGCTCTAGAGACAAGAAACACTGGATCTTTGACCAACATACCCAGGAAT
ATCCTGGATAGGAGTGCTGTCAGCCTCATGCTTGTTTCATTATGGTGGCAAAATTGTCACCACAGTTCCA
GATATCACATCTAGGTTAAAGTTTCCTACTTTATTTATTTATTTTTGAGACAGGGTCTCATTCTGTCACG
TAGGCTGGAGTGCACTGGTCAATCATGGCTCATCGCATCCT

RPL32

CCCGCTTCCCATGAATCACTAGGTCACAAAGCCCCACCGGGTGAGGGAACGTGTGACCCACTCAGACTGC
TGTTTCAGACTCTGCTATGAGGGTCATGGGAAGAGAGCACTGGCCCTCCTTGGGAGAAGATTCTGGTCCA
AATCTATGGTGTGGCTCAGCTATCAGCAGTGAAGATTGAGGGGCACTGACTGGTTTCTTTCCCCACATTC
ACCCTCACCAGCCCCCCGTGGTGAAGAAGGTTGGGTGTCACTTCCTCCAACCTCTGCAGGGCAGAGTGTG
CTGCTATTACAGGGCCTGCTGAACCAGCATCTCACCCCTACTGAAGAATGGGTCAATAAAGATAGAAAAG
TTATGACAGAAAAGGAGGGAAGGAAGGAGAGTCCCTTCCAATGGTCCTCACAATAATACCACCCACAGAG
ATCGGTGTGAAATTTTGCTTTCAGAAGTAGAATGTGCCCAGACAGAATCAGAACTGCTCATTTAACATGG
GAACGGCGAAGCGCCCACTCCAAGACTTCTGCTAGTGGGTGGCCAGAAAGAAGCCATCCTCTCTCCCTTC
CTGACTCAAATTGTACAACTGCTCAAAGGAAGAAAAAGAAGCCAGAGGTTCCTCTGCATCAGCCAGAGGC
CAATCACCCTGATCCAGGCTGCCAAGTTAGAACCCAAGCTCCCAGATCAAGGGACAAGTCAGAGGTGGAG
CTCTTTGATCAAGGGGCAGGTCAGAGATCAAGGTCCCCATTGAGGCCCGGCAGGGACGAGGCAAAAGACT
GGGGCAGGCTGGCAATCTTAAACCCCATTTGCCCCAAGCCCCTAAAAAAAAAAAGTATTCCATAAATACT
TGCTGAATCCAAGAACCAAACCTAGTACTTAGCTTATACTGAAAGCTTTAATTATACTATCTCATTTAAT
CCTTACAACAGCCCTTTAATTGTTATCACCCTATTGTATATAAGAAAAAAACTAAGACCTCAGTAAAAAA
AAAAAAAAGAAAAGAAAAGGCTCTCTTCCTCGGCGCTGCCTACGGAGGTGGCAGCCATCTCCTCCTCGGC
ATCATGGCCGCCCTCAGACCCCTTGTGAAGCCCAAGATCGTCAAAAAGAGAACCAAGAAGTTCATCCAGC
ACCAGTCAGACCAATATGTCAAAATTAAGCGTAACTGGCGGAAACCCAGAGGCACTGACAACAGGGTTCG
TAGAAGATTCAAGGGCCAGATCTTGATGCCCAACATTGGTTATGGGAGCAACAAAAAAACAAAGCACATG
CTGCCCAGTGGCTTCCGGAAGTTCCTGGTCCACAACATCAAGGAGCTGGAAGTGCTGCTGATGTGCAACA
AATCTTACTGTGCCGAGATCGCTCACAATGTTTCCTCCAAGAACCGCAAAGCCATCGTGGAAACAGCTGC
CCAACTGGCCATCAGAGTCACCAACCCCAATGCCAGGCTGCGCAGCGAAGAAAATGAGTAGACAGCTCGT
GTGCACGTTTTCTGTTTAAATAAATGTAAAAACTGCAAAAAAAAAAAAAAAAAAAAAAGAAAAGAAAACTA
AGGCCCAGGGAGACGAAAGAATTTGTTTGTTCAATGCCAGACAGCTTATCAGTGTCAGAGCTAGCAACTG
AATGCCCGGTCTCTAGACTCCAAGATTCATACTCTTATTCCATAACATAATTTTTTTAAAAGGGGGAAAA
AGACTAGAATCAGGAAGTAAAAAGATAAATGTAACAGTTTCAGAGATTAAAGGAGCAAACCACAAGGCAA
GAGACAGGTATCCTCCTTGCTCATCCACTGGGAGAGTCTAGAAGCCTGACACCACAGCACCAACTAACAC
AGCAGGCCCAGCTCTTGGCTTGTAATCTCAATCTCTTATCAAAACCCAAGGACTTCTTGGAGAAAGGGTC
CATTCCACAGCCAGGGCAGGAGAAGAATTCAAACCTGGAACTTCCTGTAGTGTACAAGAAAATAAAGAAG
TGCTCGAAAAATGATGCAAACATGTTAACAGGAAAAGTCAA

RPL37

TTGGAAGGAACTTAGTTTATAGTTTGAAACAAAGACATTAACAGCCCTTTCCCAAAACAAACCCCCTTCC
TGCTTGGGGACTAGACTGCCTTTGTAAGACTAACAAATTAGGCACAAGGTGAGAGATGATGGTTTAGGAG
TCATGTAGCTGGAGGCTACAAGATTCTCACCCTTCCCAGATTGTTCCTGGGGATAACATTACTATTATAA
AACCTAAGATCAGTGCTTGAGATATTTTGCATTCCCTGCACTTGATGGATCACCTGACACCAACCCAGAT
CCATAAACTGGCTCATCTGGTCTTGTGACCCCACCCAGGAACTGACTCAGTGCAAAATGACAGCTTCAGA
TCTCTATGATTTCGTCTCCTACCCAACCAATCAGCACTCCCGACTCACTGACACCTACCTATCAAATTAT
CCTTAAAAACCCTGATCCCCGAGTTTTCAGGGAGACTCATGTGAGTAATAAAAAAATTTCAGGTTCCTGT
ACAGCCAGCTCCACATGAATTAAACTCTCTCTTGCAATTCCCCTGTCTTGATGAATCAGCTCTGTCTAGG
CAGCAGGGAAGGAGAACCTGTTGGGCAGTTACAACCAAAAGGGAGGGTTCTTGGATCTCATGCAAGAAAG
AATTAGGGGTGAGTTCACACTGTAAAGTGAAAGCAAGTTTACTAAGCAAGTAAAGGAACAAGGCAGGGTG
CAGTGGCTCACGCCTGTAATCCCAGCACATTGAGAGAGGCTGAGGTGGGCAGATTGCTTGAACCCAGGAG
TTCAAGAGCAACCTGGGCAACATTACAAAACTCTATCTCTACAAAAAATACAAAAATTAGCCAAGGGTAA
TGGTGTGCACCAGTAGTCCAAGCTAGTTGGAAGGCTGAGGTTGGAAATTGCTTGAGCCTGGGAGAATTTT
TTTTTTTGAGGCAGTGTCTGGCTCCGGTCACCCAAGCAGTAGTGCAGTAATGCCATCTCGGCTCAGACTC
TAATTCAACACGGAATGTTGAGCTTCTGAGCGTGATATAGCGGAAGTGCCTTCTCTTCCGGTCTCTCTGG
TCTTGGCCGCAGAAGCCAGATGACGAAGGGAACGTCATTGTTTGGAAAGCGTCGCAATAAGACGCACACG
TTGTGTTGCCGCCGTGGCTCTAAGGCCTACCACCTTCAGAAGTCGACCTGTGGCAAATGTGGCTACGCTG
CCAAGCGCAAGAGAAAGTATAACTGGAGTGCCAAGGCTAAAAGACAAAATACCACCGGAACTGGTGGAAT
GAGGCACCTAAAAATTGTATACCACAGATTCAGGCATGGATTCTATGAAGGAACAATACTTAAACCCAAG
AGGGCAGCTGTTGCAGCATCCAGTTCATCTTAAGAATGTCTATGATTAGTCATGCAATAAATGTTCTGGT
TTTAAAAAATACAAAAAAAAATACTGAATGTTTACTCCTGAAACTTATGATGCCTAGGTTGAGAAAGACT
CAATTTTCCTCATGCTAAATACTTTATACATGTCCTTTATTCATGCATACATAGGACACTTTACACAATA
TAATTGTTGAAAGTGCTTCTGAAATACTGTTCAAATTGTCTATAATATTTTTACACATGCACTATAAAAA
TTAAATTTATCAGACAATAAATATTAAATAATTTAATAAAATTGCATTTTTCAAAGGCTCTAACAATGCA
TATTTGAGTTATCATATTAAACATTGTATTCTATGATTTTTCAACTGTAATATAATTTTTTTTGAGATGG
GATCTTGCTCTCTTGCCCAGGCTGCAGTGTGGTGGTGCAATCACGGCTCATGCAGCCTCCACCTCCCCAG
GCTCAAGTGATCCTCTCACCTCAGTTTTTGTGTTTTTAGTAGAGATGGGGTTTTATAATGTTGCCTAGGC
CGGTCTCAAACTTCTGGGCTCAATCTGTCCACCTCAGCCTCCCAAAGTGCTAGGAATACAGGCATGAGCC
ACAGAGCCCAGCCAAAACTACATTTTAAAAATAATTTTTTA

Supplementary Figure S2. The occurrence of the ZTRE in the region 1 kb 5' and 1 kb 3' to the transcription start site for ribosomal protein genes with transcripts increased in abundance by knockdown of ZNF658 in Caco-2 cells. Transcription start sites (taken as the 5' end each sequence specified) are highlighted in pink. Sequences matching the ZTRE segment C-A/C-C-T/A/G-C-C-C/T are highlighted in yellow. Sequences matching the corresponding complementary ZTRE segment A/G-G-G-C/T/A-G-G/T-G are highlighted in green. **Sequence in red and underlined matches a ZTRE segment that overlaps with another ZTRE segments**. Cyan highlighting between two of these segments indicates a space smaller that 30 bases; blue shading indicates a space between 30 and 50 bases.
