# Supplementary material for: The Zinc Finger Protein ZNF658 Regulates the Transcription of Genes Involved in Zinc Homeostasis and Affects Ribosome Biogenesis through the Zinc Transcriptional Regulatory Element
Source: Mol Cell Biol. 2015 Feb 18;35(6):977–87. doi: 10.1128/MCB.01298-14 (PMC4333095; doi:10.1128/MCB.01298-14)
Supplement: Supplemental material [file MCB.01298-14_zmb999100760so3.pdf]

VPS2 (adjacent to RPS18)

ACTTCCCCAGGTGAAATGTTTTGGCTTGACCTTAGACCACTATCTTGGCTTTAATCACTTATTTTCTGTG
GTAAATGCTAAAGGGGTCAGTCTCTCCCTAAACCACTTTCTTTTCTTAATCCAGCAGTCAAGGTGATCTG
GATGAGACTCAAGTCTCCCCAGCCTTGCTTCATTTTTGCTTATGTCTTCACAACCCAATTCTCATTTACA
TTCCCTATTCCTTACCCTACTTCACTTACCTTAATGGCAGTGATGGCAAAGGCTATTTTCCGCCGCCCAT
CGATGTTGGTGTTGAGTACTCGCAAAATATGCTGGAACTTTTCAGGGATCACTAGAGACTAGTTGAAAAA
GTACGGGGGAAGAAATCAGACACAGTAAACTATCAAACAGTAAGGCCGATAAGGCAAACGAACTTCATAC
AAAAAGGTTAAGCCTGTCACACAAGTGGCAAGACACCCGCCCCCCATTCCTTAGGTTTTCACACCACAGA
GCAAACTCCTCAGTCCTGCAGAGTAGGCACATGAAGCGTTCAAGCTTGGGAAAATGCAACCTCTGGAGAG
AGGAAGCAGGTCTCCTGCCCATTCCCAAAAGTGCGTGGCAAAAGCCAGAGTTCCCCATATACGCCCAAGC
CTTAGACAGCTTTCGTGCGCCCGAGCAGTGACCCTTCCTCGGGCCCGCTCTCCGGAATTGGAAATCTTTG
GTGTCCCTTTCCAGGGACGCGATCCAAGGCTCCATACAGAAAGTCGCAGGCCCTCAGGGCAGGACGTTTC
CAGAACCCTGACAGCCGGCTTTCCTTGCCCGAGCTGCGATGCCGCTGGATCACGGCACGGATTCCAGTCT
TACCATGGCTGCAGCACAAGCGGCGGCGTGTAGGCCTCCTGTGGAAGAGAGAGCGGAAGTGACGCGATAT
CATTATATAGCTTCCAGGAGGAAGAGGCGGTGCGATCTAGGAGAAGTGCTTCCGGAAACTGTTTCAGCGC
GACCTGGCTGGATTTATGAGAGTATGGCTTGGGTACCACAACTTCCGGTGCGCCTTTCTTTACAGTTCGT
AAGGTTCATAGGTAAGAGAACAGCGAAGGTTCCGGGGCTAGTTTGTGTTTCAGACTTCCATATTTCCATT
TGCAGTTGGGTGAAGTGGTTAGAGCTGAAAGGGGACAGCTGGAGTCAAACAACTTAACTCAAATATCTGG
GAACGATTTCGCGCTGAGGAAGACCCTTTGGGACTTGTAGCTCCACTCCGGGGAACGGACTCGCCGGGAC
TGACAGTTGCCGGAAGTGAGGCTGCGGGGAATGGCCGCCGCTGCGACCATGGCGGCTGCGGCCCGGGAAC
TGGTGTTGCGGGCTGGGACCTCAGATATGGAGGAGGAAGAGGGCCCGCTGGTGAGAGCGCGGAGCTGTTT
CTCCTCCGTAGTTCCGGTGCTCCTAGCTTCAGAAAGACCTGAGCTGGGTTTAGGCACAAGGGTGGGAACT
TGGCTGCTACCCGTGATATTACCTGTTTGGCTCCCCGTACCCTTCCCTGTGATCAGACCCGACCCTGAGC
GGGACCCTAGTGGCTGAAACTCATGTTCTGGGGCACAAAATTTCAACTTCACTCCACATTTTGGGGTAGA
GTCACGAAGTAGTATAGTGTAAAGGTTAAGAACATGGCCTCTGAAGCCAGACTGCCTGAGTTCTATCTCT
GCCACTTACACGCTGTGTGCCCTTGGGCAAGTTAACCAGCTTCTCTGTGCCATCATTTCTCACACCTTTA
AGGTGAGGAAGATAGAACCTATTGTATACATCGTCGTTAGGATTAGATGAATATGTGTAAGATTGCACCT
GGCATGCAGGTAAACACTATGTGTTTTGACATTTCTAGAAAGCCTGGGGTTCCCCAGAGGTGTGATAGAA
CACCCAAGATGGCAGGAGTCTGAGGGTCTTAAATTTAAGTGCAAGTTCTGCTCTATCCTCTGCAATTCCT
AGCTTTCCTCATTCGGTCATCTCCACTGCACTTTTTTTTTT

IKZF4 (adjacent to RPS26)

AACTCCTCCTCTGTACTTTGAGAATTTTAGATGTCAGCAGCTACCTTAGAAAGGGAAAAGAGAAAGTCAA
AGTAAGGCTACCATTAGCCCTGAAAAGTACCCCTCTTAGGACTGGGCATAGCTGCGCTCATCACTGACCC
ATCTCTCCTGGAAGAGTCATATGGAAGCAGAGGGAAAGGCTGCAGCATGCAGCCCTCCCGAAGCACACTG
CGGGCCTCCACAGGCCATCATAGTTCTTCTGCTCCACGCCAGGTGACCTTCCCTCATCAGGCCACTTGAT
GAAAAGGGTATCTCTCCTGTTCTTCCAAGAGATTTGAATTCCTCACAGAATGTGATTCAGAAAATCCATC
TTTTCTCCCCTTTTCTCTTTTTAAAGGTACAGATCATTCCCTTCTGGGGCAGGGAAAAGGGGGAGATAAG
CAGGAGTCTATTAATTTATGGCACAGATAACCACCCCCTCCATTTTCCTATCTTTCAAGGGAAACAGAGT
TGCTGAAACCCAGCTCCTGCCATTAATTGACTGAGCAGGGCTTGCCCCAAGTTTTCTGAGACTGTGAAAT
GTTTTAGAGAACTTTAGTAGAAAGGGATTCAGGGGTAGGCAGCCCGAAGTTTCACAGCCCCT**CCCACCC**C
CAAATTCTTGGAATCAGAAAATGTAGTCTTCCAAAGTAGGTCAGTTTGTCAGTGAGCAGCTCCTCTGAAA
GGGAAGGGAGCAGAGGCCTTGTCCTGATAGAGGGCGGATTCATCCAGGGCTTTCGGCTGCTGGGGCCCCA
GGGGCACAGGAAGGGGGGGAAATGGGGAGACCGTACCAGCCTTTTTGCCCGGGATCTGAAAGGTCTCAGG
GCCACGGAGCAACACTAGCCTGACACCAGACTCTGCTGGGCCCTCTCACTGCTGGAAAGGAGGAGGCTGT
CAGTCCCCACATCTAGAGAGCAACCTTCCTCCTGGAGGAGGAGGGAAAGAGTTCGGTGGGGGAGGGGCTT
TCTGCCTCTCTGAGAGCCTTGAAGCTGTCCGTGTCCTGGGCCCCATGACCTCTGGGGCCTTGGCTTCCCC
AGCTGGCAGAGGATTGGGCCTTCCCTAGGGCCCCCCCTTTCTCCCT**CCCACCC**GCAGGCCCATCCATCTC
TCTCTCTCTCTCTTGCACACACTCTTGCCTCTCTCAGGCATTTGTTGTGCAGTTCCTCTTTGTCTGCTGG
GCACGAGGGGCAACAGCATCTGCCTTTCCCTCCCTGTGCACACACCCACCACCCACCCCCTTCACTGTCT
TGGAAAAGGGATGCTGTAGCCTAGCATCTCCCCCACTATATACACATATACATTCTCTCCAGCCCCCTCC
CCAAGCACATCCAAGCGTGCTCTCCCCTCTCCTTCTCTCCCTCTCTCTCTCTCTCTCTCACACACACA
CACACACACACACTCAACACACATACACCCTGGGCTGAGCTGCTCTTGCTGGCTGCAGCCGTGGGCCTCT
GCTCACCGTGCCGCTGCTGCTGCCTGCGAAATGACGGCGGTTCCCCTCACTTCCAGGAATCCACGCTTCC
TGGAAGGTGAGTGGCTGGGCTCACCCCTGCCTGCCACTGAGACGCAGACATGCATACACCACCCGCACTC
CCTCGCCGTTTCCAAGGCGGCGGCCGCGTTCGCACCCCAGGGTCTCACCGGCAAGGGAAGGATAATGTAA
GTTCAGGCAGAAGGCGGCTAGTGGAGGGAGGAAGGGGGGTGCTGGGGCTAGGGAGCCAATTCAGTAACTA
CTCCCAAGCCTGAGGAGATGGAGAGGATGGGGGCTGTGTGTGTGTGTGTGTGTGTGTGTGTGTGTGTGTG
TGTGTGTAGCGGGGGAGGGGGCGTTCCTCCTTATACTTGCGGAGGATGGGAAAGGGTGTTTGGTGTTGCT
AAGCAAAGACGGCGACTTGGACAGTGGGAGCCTATGGTGACATCTTGATTAGGCCTTGGGGATAGAGGTA
GAGCTGCCTGGGGTGAAATGCCGAGACAGGCCTGGGTGGGA

CREB3L4 (adjacent to RPS27)

GAGCGTGTGCGCACAGCTGCTGTTCTCCATTAACGCCACACTGCTCGGCTTGCGGACGGGGAAACCCGCC
ATCGCGCTCCTGCCGCCTGCCGAGCGAGGATCACCGCTCCACTGGGGTCAGCGGGCTGTTGACTCCCCGC
CTGGGTGAAACCGGAGCTTCCGAGTCACGTGGCGCGCGAAGACAAACCTGGCTGCTCAGGGTTTCCCGGA
GCTTTCTCCCAGCTTCCCGGCGATTCATATTCTGGCGCTTACCCCTCCCTTTAGGCTGGCGGAACCCAAT
CCCCGCCTGACAAATAAGTCCCGCCGGCCGGACGCAGTGGCTCGCGCCTGTGGTCCTAGCTGCTCGCGGT
GCTGAGGCGGGAGAATCACCTGAGCCCGGGAGGTCGGGGGCTGCATGGAACCGTGATCGCACCACCGCAC
TCCAGCCTGGGCAACAGAGCGAGACCCTGTCTCCAAAAATAAGAATAAAGAAAAAAAAAAGAAAGAACTG
CGGCCCCATTGGCCTGACTCTCCTGAACAGCCAAACTGTCTCCGACCAGTTACGGAAGAGGCGGCCTTGA
GAGGGTAGTGTGCCATTGGCTCGATGTCCTGCCCCTCCGAAACCTAAGTCTTCAGCTTCCAATCAGGACT
CAGCTTTGGGAAGAGCGCCACGCGGTGGGCGAGGGGATGCCCTAGTAAATTCGCAGGTCTCTTGACTCTT
TCCGCCTTTGTTTACAACCCTGCCATGATCTCCCTCTTGCAAAAGCGAGGGCTACAGAACAGGCATTCAG
GAGTCCTGTGCTCCAGTCACAGCCTTTTCTGTTCTTCAGCTAGGAGACACCAAACCCTCAGGAAGATTTA
CTATAGCTAAGAGAAAACTGCAGCAGAAAGGGCGCGGCTACCTACTTCTTAAATTCCGTTTGTGGACCCT
CAGACTCTTAGTCCCCTACTCCCAGATACAGCGGCCCTACCGTGGCTCCTGGCAAGGTGGCATCCACTTT
TGTAGTGTAAGGCTGG**GGGC**GGGGTTTGGCTGGCAGGTCCAGGATACGAGATCCTGGAAGAAAGAAAAGG
TGTAGTGTTTGGGGAGGTCAACGGGCTATGCTGGCTTGACAGGGCTGGGCTCTTCAGAACAGGTAATGCT
TGGGGGGGGCCTCTTTGTCTAAACCTGAGGGATAGGGCTGAAGGTGAAATATCGAGGGGACAGGTGAGGG
GGCTTGCTAGTGAGGAAGGAGTGGGGGGGCGGGGATAATGGAAAAGGGAGCAGAGGAGCCCAGAAACTGT
AGGGGGTAGTAAGCAGCCATCATTCCGTTTCTGCAACCCTCCGTCCCACACGCCTTCCTGCAGAAGCATG
GATCTCGGAATCCCTGACCTGCTGGACGCGTGGCTGGAGCCCCCAGAGGATATCTTCTCGACAGGATCCG
TCCTGGAGCTGGGACTCCACTGCCCCCCTCCAGAGGTTCCGGTAACTAGGCTACAGGAACAGGGACTGCA
AGGCTGGAAGTCCGGTGGGGACCGTGGCTGTGTGAGTGTGACGAGTGGGAGTGGGGGTGGGGTTGAGACA
CAACTCTGTGATAAGAGGCTTCAGCTCCCACTTGGAGACAGGTCTGAAAACAGACCACCCCCAAAATAGA
CGTAAGTTGTATAATAACTGTCTCCATGCCCAAGAAGTGAAAGGACTGAAAATGAAGCATAAGTCAGGGA
GGACTTCCAAAATTCTTCCCTGCACATATATATAACCTTTTCCTACTGTAGGGCCTTCAAGAGAGTGAGC
CTGAAGATTTCTTGAAGCTTTTCATTGATCCCAATGAGGTGTACTGCTCAGAAGCATCTCCTGGCAGTGA
CAGTGGCATCTCTGAGGACCCCTGCCATCCAGACAGTCCCCCTGCCCCCAGGGCAACCAGTTCTCCTATG
CTCTATGAGGTTGTCTATGAGGCAGGGGCCCTGGAGAGGATGCAGGGGGAAACTGGGCCAAATGTAGGCC
TTATCTCCATCCAGCTAGGTCAGTGTTCTTTGTGGGAAGGG

LRR1 (adjacent to RPS29)

GCCAGTGCGCGATTACAGCTCACCGGAGCCTCTCGACCTCCCCAGGCTCAAGTGATCCTGCCACCTCAGC
CTCCCCAATAGCTGGGACTACAGACACGCGCCACCACGCCCGGCTAATTTTTTTGTATTTTTCTTAGAGA
CTGGGTTTTGCCATGTTGCCCAAACTGGTCTTGAACTCCTGGGCTCAAGCGACCTGCCTGCCTCAGCCCC
GCAAAGTGCTGGGATTACAGGCGTGAGCCACCGCGCCCGGCCATAATTTTATCTTAATCACTTTTTTCCA
GGAACTATTACGTTTCAAAGAATGAGGAATTAACTGACAACACTTCAGGTTCAACATGTAAATAGGGACG
CTCATTTGAGAATAAATCACATTTTGTCACAGTTGTATTGAAAAATCGGTATTTCTGAACGTCATTTTAC
CATTACGGAAGTATAACTCAGACACAATAAAATGTTTAATATACGGCTAGGATTAGGTTAAGGTGAGAGA
GGCGCCTAGAGCACAAATTTTAAGGAGGCAAGACCACGACCCCGACCGTGAGCGCCTGCTTAACATTTGC
TCCCCAGGCGCTTCCCACTCCCCAGCCGGGCCCCGTCCTAGTTCAATCAATCAAGAAAATCAAGAGTACG
AAGGCCTTGTGCGGTCTGGACGGTCTTTCCACGTCTCAGGTGCAGTTAAGCCAGTCACACTCCATCCACC
TACCCGCCATGAAAAATTATTTGTTCAGCAGGAATTGCCAAGTCAAATACAGCCATATAAAGCCCCCCTT
CGACGTGCCAGGTGTGCCTCCGGAGAGCAGCCACCAAAACCACCAGCAGCCCGTCCGCGCCGGGAAATGC
GCCCTCGCTGGCTTACGGGGCCACTCAGACAGTTCTAAGTGCCTTTCCCTGTAACGCAGAGGCACACCTG
CCTTCCCTCGGGAAACAGCGGCCCGACGTGCGCCCGCAACGCGTAAAGCGTCAACAGCTGAAACCCTCGG
AATCCTCCCCGAACAGAAACAACCACCGCTGCCAGCTGCGCGCTCGGGGGAAAAGACGTTGCGCCCCCGC
CGACTGCCGGTTTCCCGGGCGCGAGCCCGGATCCAGGTGGTCAGTCCCGGTACGCAACCACGGCGAGAAC
CCGGCCCTGCTAAGGGAGAAGGGAAGCCGTTTCCCGCGGGCTTCATACAGACACGGTGCTAAGACAGCCC
GAGTCGCGGACCAGTCCAACAGGCAGGCAGATCAGTGGGCAGCCCGCGTGCGCGAGGACCCCGATGGCGG
CGCCGGGTGGCGGGAAGGAGGAAGTTTCAAAGCCAGCTTGACGTGGTTGTGGCCGTTGGGCGAGATGAAG
CTACACTGTGAGGTGGAGGTGATCAGCCGGCACTTGCCCGCCTTGGGGCTTAGGAACCGGGGCAAGGGCG
TCCGAGCCGTGTTGAGCCTCTGTCAGCAGACTTCCAGGAGTCAGCCGCCGGTCCGAGCCTTCCTGCTCAT
CTCCACCCTGAAGGACAAGCGCGGGACCCGCTATGAGGTGCGTGAAGTGGGCAGGCCCTGTCAGTCTCGC
GTTCTTCTTGGAAGCCGAGACGCGGGCCACCCTCGGTCCTCATGCTCCCGGCTGCTCCCTAGGCGAAAGC
CCGCCTTGGGGGTTCCTGAACTCCCAGCCTTGAGACCTACCATCAGCCCGACCCCAGGGTCCTGTGCGTC
TTCCTACGGACCCGAAAGAAGAAAGCTTTGAGAGTGTACCTTTTCGCTATTTTTCCTCCCCACTTTTACG
ACTTTGAATTTACAGTGTTGCTATTTAGTAGTGGATGGCAATCCCGCCTGTTTCAAGTTTCTGAAATTTT
GCGTGAAACAAGCGCAAATGAAGCAGCTGTCCAGTTGGGGAACAGTAAAATAACTGCAGTTCTGTTCAGT
GAATTCTTTCTCCCACAATATTCTCCCTAGCTTAAAAAAGCAGGTCGGCCGCCCTCCCCACAGTTGCCGT
CTCCCCCGGGGCCGGCCGGTCTTATGATCCGGCGGATCCTC

HECTD4 (adjacent to RPL6)

GGCCTCAAACTTCTGGGCTCAAGCAGTCCTTCTGCCTTGACCTCCGTGCTGGGATTACAGGTGTGAGCCA
CCGCACCCGGCCTAGTGAGCATATTTTTAAGGTGTATGTCAATTAGTGTGGAACACTGATGGAACGAACA
TAGCAATAATTAGAATTGCCACTCATGGAGCACATATTAAGTGTCGGGCATTGAGCACCAAGCATTGTGG
ACAGACGCTTTATTTTCACCAGGTTTTGAGTGCTTACTGGGTACTAGACTTTTCAGTCAATATTAATCAT
CTACTATGTGATTCTACCAAGTGCTTATGAACAAGTGTGCAGCGCCTATTCTGCGCCAGCCTTTTGCTTG
CATCTCATTTAAACGTGAAAACAAGGAGTGATCTCTATGCTCTGCCCAGGGTGGGGGCGCGGGCCCGAGC
AAGGGGTGCACACCTGAGTAGGGACAGGACGGGGGAAGCGGCGTCCTCTTCCCTGGCGCGCTGCAAAGCC
AGGGCTACCCTGAATGGCCAGGGGGAGAAGGGGCCGGCGGAGGTCAGGCCGAGGGCAGGCGGCAGCGAAG
GGCTAGGCGGACGTCCAGGCCACCCGGGTGGCCTCGTCCGCGGAGCGGTGGGCGGAGCGGCGGGCGGGGC
CTGTATTGTCCGCTCCGCGAGGCATGCCGGGAGCCCCGCCCCCAACATGGCGTCTCACTGACAGTTGGTT
GCCGAGCGGCAGAGGCGGATCCTGCTCCTGAGGGCGGCGGCGGCGGCGGCGGCGGCGGCGGCAGGGC
CGGGGTCTCCCGGGCCGGGGTCGGGGGTATGGCGACTCGGACCGCCTAGGGCGGAGTTGCACGGGCGAGG
CAGCAAGTGGGGCGCCGCTGGCGGGCGCGGCGGCCGCTGCCATGGACTGGTGATCGCGGCGGCTCCTGCT
CCGTTTCCCCCGGCCGGGCGTCTGCTCCTCAGCGCCAAGCCTTCAGCCGGGGCCATGGGCTCGTCGGCGG
CCGCGGCGGCGGCGGCGGCGGCGGCCGCTGACTCGGCGCAGTGGCTCTCGGTGAAGGAAGAGACCATCTT
CCTGCACGACGGGCTGATCCGGGTCACCGACCTGGCCGAGCTGCCCAGCGAGATCCTCGGGGCCCCAGAG
GCCGCGGACACCGACCTGGAGGTGAGCGAGGCGGCCCCCGGCCCCCGCCATCGGACTGACCCACTCGCCC
GGCACCCCCGGGTCGGGCCCCCTCCTCGCCTCAGCCGCGGCCTCGCCTCCCGGCGGCTGCCGGGGCAGGT
GTGTGTGGGCGCGGGGCGGGCAGGTGGGGCCGCGGCCGCAGGTCTGCGGACCGCCCGCCCCGCTCTCCCT
CCCTCCCTCCTCGGGGCCTGGCGCCCGCAGGGGATTTCATTTGTGGTTTTCTTTTGAGCGTTCCGACACC
CGGCATTTTATTAGGCTAGCGCCTGGCTCGGCCTTCATGCCCCAGAGGAGCCACCGCCACCACCCGGGCC
CTCCCTCCCCTCCCCCACGGGCCCCCAGGACCCACTTCCAGGGCACACCCAGGTTGCTTAGCTTTGGGAA
CATTTTGATTTTCACGCTGTGTACCCTCCCATCCTTTTCTTCCCTAGTTCTGTTTTTTTTGTTTGGTTTG
GTTTGGTTTTTTTCCGGGAGAGGGTAGTGGCAGCTGCGGGTCCGCGATGGGCAATCCAGCAACTTCTCTG
TGTTGTGTCTCGGATGGATTGACACGGGGCCTTTGTCAGGGAAGCGGGCGCTGTGGACAGCGAGAGGGAT
GCTCCAGCGGCCCTCCATGCACTCTCGGGGAAGGGTCACTGGCGCGTTAGGCCGCAGGGCCTTTTTGTGT
GCCCCGGGGGTCGCCAAGCATCCCGGGTTCAGAGCCGTGGCAGGTGCAACAGCAGATCTCATCAGTTGAG
CTGCTTGGCCTTGGGGGCAGAAGGGAACCAAATATGCCTGGTTGGTGGGAACTTTCCATCGACTTCACTT
GGGTTCGTGTTTGCCTAGAGGTGGGAATACTGGCAGTGTCG

UGDH (adjacent to RPL9)

TATATGGTATTTTAATGTGCCTTTATAAATTACTAATCACTTTCTTCATGATGTATGTTAAAATGGCTTA
TGTCACCTGATGTAATCCTTAATGAGGGAAATAAAAGGCCATGATTATGCCTGGCTTTTTTTCTTATATG
TTAGATTTGAGTGTTAAAATGACCTCGAAGCGGCGAAAGAAAGGAACGGAACTGCCCTCCACATTAAAAA
TGAGCAGGCTCCATAGGTTAATTTTACTCTTCCTCACGCACGGCACTTACATGTTGCAAACTCTCTGCTC
CACAGCACGCACAGACATCCCAAATCCCGGATTGGGCCTGAGGAACATCTCTGCAAAGAGCGCCAGGA**GG**
GCGTGGGGGTCCCGCCTCTCTCGCCAGGGACCAGGGAGTGGACACTCACTGGTCCAGTCATGAGGAACTC
AGCATCCTCCTGACGGGAGGCGCCTCTTTCCCAGAAGCGGTGGGTCAGGAGGAATCTGACTAAACCCTGG
GTTGCCGGCCCGGAGGGCGCGGTTGACCCCGGGGCCTCGAGGTTGTGGGCGAGGAGTAATCCGCCCGCCG
AGACCGACCCGCAGATCCAGGAGACCGAAACCCGAGGGCGGCGCTCCAGGGTGCTGAGGAGACCGGAAAG
GCGGCCAGGACCTTCCGGTCGCCTCGGCCCACCACCCACCCAGTGTCCGCGCAGCCCTAAAGCCTGGCAG
ACGCGGAGCGCACGCGGCCTTTGCCTCGGCCAGGACAGGGCAGCGGGGAGAGGGCTGCTAGACAACAATT
CTTGACCCCTCCTCGACCCACGACCGCCAGGTAGGCGCGCAAAGCTCCTACACCTGCGGCGCGCCCCCGT
CCCCTGGTCCAGCGGACACGCCACGCACGCAGCCCCGCCCAGCGCCGCTCGCGGTAGGGGCGGAGCCGCT
GAGCTGGCCAAAGCGGATTTGCCCAGCTAGGCCAGGGGCGGGGCAGGCCCTCCCAGCCAGGACCAGCCCC
AGTCTCCCCGCCCCTGAGCCGTGAAGGAAATAGGGACCTGGCCCTGGGCCTTGTGTAGCGGGAGGGGGAG
CTAGGAAGCAGCTGAGGGCAGAATCCAGGAGGGCCTGGCTGCGGGGGAATGAAGCCTCCGCCTTCGCAGG
CAAAAGCCTTTAAATACGGGCTCAGGCCCGGGACTCAGAGTGTAACGCGTGGCAGCCTGAGGGAG**GGGCG**
TGCGCCGAGAGGGAGCTCAGATCGAGCGGGGCGCGGGTGGAGAAGCTGCGGCGGCGCGGCCCGTAGGAAG
GTGCTGTCCGAACGATCGGGATAGGAGCGGTCCCTGCGCTTGCTGCTGGGAAGTGGGTAAGCGCCGCTCC
CAGGCTGGCCCCGCCGCCCGGGCGGGATGCTCCCAGGTCTGGGCGGGATGTGGAGGGAGCGGGGCTGGGG
TGTGCGCGCTGCTCCCGGTCGCTCGCTGCGCTGGGTCCCGGGCTTCCCGTCTCACGACCTTCGTCTCTTT
GGGGCTTAGGGCGGGAACCGCATTTTGGAACTGCTGTCCTGGGGATGTGGTCGTCTGGGCTTCTTATTCA
AGACTCGTGAGTCTTCCAGCCCCTTCTCTTTCTGCCTTTACCCACTTTCCTGACTTGGATGAGATTTCCC
TGCCGCCTCCAAAGCCGAACGCCCACCACAGGAAGCGGTTCTCTCGTTGGGGACCTCATTTTGGCCGCAG
AGTCGTAGTCTTTTGTTTGTGTTTAAGAGTCTGCTCACCTGAGGGGCTCCTGCTTTTCTCATGCCGCCCC
TCTTAAGAAACCAAAGAGAAACGAAGCCAGAAAGCCCTCTTGAGAGCAAAATACTAAGACTGCCGTGATA
TCTGTCCGGGATGTCCTGGAATCCATTGTTGGCCCCCTACACCACAGAATTGCTCTGTTTGATGTCGGGG
AAGGCCTTTCCTGGGTGCCTCGGGAGACCATGTTTGTGTGAGATCTTGAAAAATTATACGTCCTATAAAT
ATATAGGAAGATAACTTAGTAGGGTTAGGAATTCTAGTATA

C18orf32 (adjacent to RPL17)

ATGTCAGAACCCTGTCTGTTAAAAAAAAGTAAGTGTGGTGTCACTTGCATGTAGTCCCAGCTAATTGGGG
GGCTGAGGTGGGAGGCTGCAGTGAGCACTACTGCACCCCAGCGTGGGCAAGACAGAACAAGACCCTGTCT
CAAAAAAAGTTTCATTATCCCAAAAGGAATTTGCAGTGGTTTTCTTTGCAGAACAATCCAGTATGTGAAT
ATACCAGTTGATCATCCCCAGTTGGAAATGGCTCCATTGAGCATTGCCTTTGAGTATGATAACAGCACTC
AGGCTCAGGTTTTGAAGCTTTTTAGGTTGATGCTCAACCTGTATCATTTCGTCAATTGATAAAGGGGTTT
TGTTTTTGCTATTAGGAACGATGGTACTGTGAACGTTTGTGTGCAGATTTTTGTGTTTTCATTTATTGAG
TATATACTAGAGTGGAATTGCTAGATCATAAGGTTACCTGAGTAAACATCTGAGGAACTTCCAAATATTT
CCAAAAAGGCTGCACGCTACATTTCCACCAACGGTTTATGAGGGTCCCAGTTTTCACACAACTTCGCCAA
CACGTACTGACATCCTAGCGAGCGTCAGTACTTTTCGTTTATAAAGTGATGTAACTGCTTCTTTTAAGCA
ACTGACGTTTGTAAAGTTGGCCTTAATGTTAGTTTGCTCTGCCCTTGAATTTAAGAAACCAGCAAAACTA
ATAGCTTATCCACAGTTGCCTCAACGGCCTAGCTGCCACCTTCCGTGTGGGTGCCGCGCCGTTTGCACAG
GCATCATCCTTTACCGAGATCCTGCGTTCTGGGTCTGGGTGTAGTCAGGTCAGGCTTTCTTCCCGCAGCG
AAGCTTTTAGTTGGGCATCTGGGACGCGCAGGACTTTCGCGCTACCTGCAGGCCGCAGAGCCGGAAGCCT
CGCCGCGCCCCTTGGGCAACACTGCTGCGTGCTGTGCGACCCCGGGTTGACGCAAGCGCGCCTTTGTCGT
CCCCGTTGAAGGCGCCCATTGGCTCGGCTGCGGATCGTCCCTCCCTCCGGAAGTGCGGACATTGTCAGCT
GCGTTTCCGCGGTCGCGGGTGAGTGTGCCCGGGCTAGCGGCCTGGGTTGGGCTTTGTAGCTGCTCCGCAG
GCCCAGCCCGGGCCGCGCTCGCAGAGTCCTAGGCGGTGCGCGGCCTCCTGCCTCCTCCCTCCTCGGCGGT
CGCGGCCCGCCGGCCTCCGCGGTGCCTGCCTTCGCTCTCAGGGTACCTTCCTTCCGCGAGCGCGCTCCTC
CGCCCTCCGCCCCG**CCCTCCC**CTCCCCTCTGTCTGTATTCTTTGTCCACCACCGGCGCCCTCGGAGCTTG
CATTTGCCTCTCCGATACTTTATATTGATTTTGTTCTTTTCCGTGGCCAAGTATCGGTTTCACATCCTTT
CTATGACCTTCACCGTTAGGCCTCTGTTCTCGTCTCTTTTCAATCATTGTCATTGACTCAACCTGTCCCC
GCTCCCTCTGTCTCCACTAAGATGACATGCTCAACTGGATGACTTGGAGGAAGGTGACTTTCCCTGTTAT
GCCTTCCCTATTTCGGATGGAGCCTCAGACTTTAACTGTCTACTCTGTTGTCTGGACTTAAGTTGAGTGA
AAGAAGATTCCTCTGCAACAGCTTTAATCCGTTTTGCTCGCTTTGTTGGCTTGGAGCGAATAATTAAAAT
AATAATATCTCGTAGCCACCTTCTAGACTTTTCTGACCCAGTTTCCATATGTGTGAAGTAAAATATCTTA
CCGAAGTATTATTATGAAGATTAAAAGATAAGATACAAATAGGCTTCATTCCGAAGAGAGCTAAAAAGTC
TCTTAAAAGCCGTAATTGTGGCGTAAGTGATTAGTAGTATTTGTCCTCGAGAGTTTATGTACATCGTACT
GTGTGCGAACTTACTTAAAACACAATTGCAGAAGTAAAAATGAATCTTTATGATGGAGGGCCTGTCAGTC
ACTAAACTAGTACTGGTAGCATTGTCAGTTGTTTTGAAGGG

RASL11A (adjacent to RPL21)

CCTCCCTCATCACAGCCAACAGCTGCCAGACATGAGTAAGGCTATCCAAGGGCAGCCAGCCCCCACTTCC
AGAACCTGGAAGCTCCCTGCAGATGCATCAAAGGGCTCAACAACTGTAAGTGATTGTTGTTCTAAGCCAT
CTAGTTTGGTGTGGCTTGTCATGCAGCAAAAACTGGTACAACCAGGGTAGAGAAAGGTATGAATTAGGGT
GGGGCTGTCTAATAGGCGATTAGGCAAGTGAGGCTGGAACACAGGAGGTCAGAGGCATGAAGGTCTTCTA
ACTGTTCAAGGTTAAAGTCCTGAGGCTTCTGCTGTGCTATTTCTGGTGGGCTCCAGAAATCCAAGAACCA
GTCTATAGCTCTGTGTCCTGTTCCTCACACTGCCCCCCA**CCCCACCC**TAGACCCCACCATGCACCTAGAA
GTAAATATTTGGATACTGAAGCAAATGCATGACTCAAGCACATAGGATAGTATTAATGATGGACACTTAG
TGCAGGGCCAGGGCCCCCAGCCAGTGGAGTGCTCATGAGTGTTCAGGAATGAGCCAGTGCAACCCCTGCC
TGCAGAGGAGGGGCTGGGCATGGATACTGAGATGCTAACCATCACTGCCCCTAAGGGGACTTCATCTATC
CCTAGCAAGGTCACAGTGACTGCAGTTGCCTCCACTGATACTAATTCAGAGCTCTTGCCCCCCGCCC**CCC**
GCCCTTTTCCCATATTAATGTTACCCCTTAGCATCTTTTCTTTTCTTTCTTTTTTTTTTTTTTTGAGAC
GGAGTTTCGCTCTTGTGGTCCAGGCTGGAGTGCAGTGGTGCCATCTCGGCTCACTGCAACCTCCGCCTCC
CTAGTTCAAGCGATTCTGCTGCCTCAGCCTCCCGAGTAGTTGGGATTACAGGCGCCCACCACCACGCCCC
GCTAATTTTTGTATTTTTAGTAGAGACAGCGTTTCGCCATGTTGGCCAGGCTGGTCTTGAACTCCTGACC
GCAGATGATCTTGCCCACCTGGGCCTCCCAAAGTGCTGGGATTACAGGCGTAAGCCACCGCGCCCAGCCC
CTTAGCACCTTTTAAAAATAAAAAGGGCAGCGTTTTACAGCAATCTGTCCCATTGTTGCAGAAACATCCA
ACTGATTGCACTTTTTCTTCTTCTCTCGTCAACTCGTAACTCCTATCTCAAGTTCACCCACAGGTCTAGT
GACTCCGGCTGGCTGCTGCGGGGCGAGGAGACTCGCAGAATGCCTGACTAGAGGCCACTCGCGCGCTCTG
GACGGTGGCCGTATCTCCAGGGTCTGAGCCCCGGAGCCGCAGTCCTGCCGCAGAGCGCCTGGAGCCGCGA
GCAGCTAGCAGCCCGGCGAAGCGCACAGCCTCGGCGATAGTCAGCTCCGCGGCCTCTGCT**CCCGCCC**CTC
CCGGCCGCTCCGGGAAGGGGCGGAGACGAGGAGGAGCTGGGACGGCCGCCGCGCTCCCGGGCGCACCAAG
CCCTTTAAAAGCCGCGGGCCTTGACAGTTCTGCAGGCAGCCGCCGCGGTCCCGGACCTCTAGTCCCGCAC
TCCCAGCTGGCGAGCCGGCTCCGGGTGCGGCGAGGCCCAGCCCTCTCGGATTGCGCGCCGGACCCCGGGA
CGCGCTCCATGCGGCCGCTCAGCATGTCCGGGCACTTTCTGCTCGCACCCATCCCCGAGTCCTCCTCGGA
CTACCTACTGCCCAAGGACATCAAACTGGCGGTGCTGGGCGCCGGCCGCGTGGGCAAGAGCGGTGAGTGC
GGCGGGGACCCCCGGGCGGCTTCGCTCCCCGGCTCCGGCCGCGACCACTGGGGGCGCTGCGGAGAGAGGG
CGCCTCGGCCGAAGCAGAGGGCGCGGGTGCAGGTAGAATCGGGCTGCTTTCGCGCTGGGTGGGTCCCGGT
CCGTCCCGGCCTGCTTCCCTGGCGTTTCCTGGTGCATCTACTCCTGGGATCTCGGCGGGATTGGCGCCTG
TTCGGGGATGGGGTGCGGGAGAGGTGTCCCGCCAGTCGTAC

TRAF4 (adjacent to RPL23A)

GAAGTTTGCAGTGAGCCGAGATCGCACCACTGCACTCCAGCCTGGATGACAGAGCAAGTCTCCCTCTCAA
AAAAAAAAAAAAAAAAAAAAAAAAAAAAGTATTTGGTGCTTACCGGGACTCTATCTCCATACTTTGGAGC
AGGGCAAGCCAGGGCCTGCCTGGACTGGCCATTAGGCCTGCCTAGCTTCTGCATAGCCCGGGGCTGGGCG
GTCCCCGGGAGTGCTGCCACCTCCCCTACACTTTCATCCTGAGGCAGGATTTGCTAGACGTCGGCTTGCC
TCTCTTCCCCAGCAGGAGGGCAGGAGTCACTAAGTGCTGCAGAAAAGATCCGCGCTGGAGATTGCCCGTC
CCCCCCATGCCTTTCCCCAGAAACAGTTGAGAGAATGGGTAGCTGGTGGGACCAGACCGGTTTCCCAGAG
GACAGGAAGGCCAATAAAAATATCAGCTGCCTGCCTGGAGTGTGGTGTCTTCAAGGGTGGGGGTGGCGAC
CAGGCACCCCAGGACTGGGCTGGGCCCGACCTCGGTGGCAGGGCTGCGGCCAAGGGAGGAATGTGGGCGC
AGCCGGAGGCTGGGGCCGAGGTTCCTCTGGCCCAGCGCCCAGTCCAGCCTCCCCGCCCCAGCTGGGCTCC
CCGGAGCTGGTTGCCAGGCTTCGGCTGCCTAGCACCTGGAAGCTGGGGCCACGGTTCCTGGGCTGCTCAC
GGCCCCGGAGGCCGAGCTGTTGCCTCTGGAGAAGAAAACCTTTGTGGCGCGGCCCGTCCCCTTTTCCGAC
CCGCACTTCCCCGGGGCCCGCGGGCTCTGGGGAGAAGCGGAGCCACGATCTCTGTCCCCCAGGGGAAAGA
AAAGCCCATGATCCCGTGGGTGGGCGAGACTGGCGGTCACCAGACCCAAAAACCATGCACTCGGGGGT**GG**
GCGGGGCCGCGAGGGCGCGCATTAGGACCGCGCGGCCGCGGTCCGCCCTCTCTTCCGGAGGGCGGGCTTC
ACAGCCGAGCCCTGGCCGCGACCGCCAGTCGGCGCCGCCCGGAGCCGGGAGCGCCGCTCCAGCGAGGCGC
GGGCTGTGGGGCCGCCGCGTGCCTGGCCCCGCTCGCCCGTGCCGGCCGCTCGCCCGCCATGCCTGGCTTC
GACTACAAGTTCCTGGAGAAGCCCAAGCGACGGCTGCTGTGCCCACTGTGCGGGAAGCCCATGCGCGAGC
CTGTGCAGGTTTCCACCTGCGGCCACCGTTTCTGCGATACCTGCCTGCAGGAGTTCCTCAGGTGCTGGCC
GGGGAGCAGGGGACAGCGGGGGCG**GGGCGGG**GCGGGGGGCGCCGCCTGGGGAGGGTCTCGGGCCCGGGGC
AAGGCGGGCCTTTGTCTGCGCTGCGACCCTGTGACCTCAGGGGCGCCCCGTGACGTCACGGGGAGGGATG
ACGTCAAGCCCCGAGGGAGGCCCCCCCCGCAGGAATCCGAGGGGTCCGGGCGGGTCCCAACTGCCCGGCC
CGGGGCGGAAGACCTCGGCGCCTTCTCGTGGGGTCAGGAGGGGAAGAGGCCCTTCCCCGTCACCTGAGAG
CCGGCGCGGCCTCAGCCTTGAACTTTGAATCCTGGGGGAAGAGGACAAAACCCGCTCCCGCGTGGCAGTT
TGAGCCTCCGGAGCTTTCCCCCATCTAACCTCTTCTGATTCTTCGAACGTTCAGAGAACGGTAGCTATTG
TTCTCCCATTTCACAGATCATGAAACGAGATTCAAAGAAAGGCTGCGACTTGCCCAGGGTCACATAGTTA
CTTAAAGGCTTAACTAGGATTTAAACCCGGACTGCTTATGATCACACAATGGAGGAAGGACTGAATCCTC
CCCCGAAGAAGGACCCCTCTAGGCGGCTCCCCTCTCTCCCGGGGGCTGGAGTAAGGCGGGTGGTCCATAA
GTCATGAAGAAGGGGGTCGCACTCGTGTTGTTACTGAACTCCCGCTGCCCTCTCAGGACGCAGGTTAGCC
CTTGGCTCTTGGGCATCCATCAAGTTTCTGGGCGCCCTGTA

RNF222 (adjacent to RPL26)

AACCTCCACCTTCTGGATTCAAGCAATTCTTGTGCCTCAGCCTCCCGAGTAGCTGAGACTACAGGCTTGC

ACCACCTTGCCCAGCTAATTTTTGTATTTTTAGTAGCAACAGGGTTTCACCATGTTGGCAAGGCTGGTCT

CGAACTCCTGGCCTCAAGCAGTCTGCCCGCCTCAACCTCCCAAAGTGCTGAGATTACAGGCATGAGCCAC

CATACCCAGTGGATGACTTTTTCTCTAATCACAAAAGAAACATACATTCAGGGTGAAAATGCAAACATCA

CAGAAATATAAAACTTAGAAAGTCTCTCCAGAGATAACTGCTATTAACAGTCTGATGTGTTTTCTTCCAG

CTTTTCCTCTATGAATATGATCATGTATTATTATCTTCATAAATAGAATTGTATTACACAGGCTGTTCTG

AAAGTTGCTTCCATTTATCATGATGCCGGGGACTTCTTTCCATATCTGTGTGCATTCCTGCTTCACTGGT

CTTACTCGCCTCGTTCTTTATGCCAGTGGTGCTGGGTCTTTGGAGTTCTAAGCGCCTTTGGGTGTTCCAT

GAATGACAAATGCACACAGGCAGATCCCACAAATATATGACTCTGAGTGTCATAGACTTCCCGAGGCTGA

TCTACTACTGACCCCTTCACCCTGTGCATGGTGTGAGGATAGGGGTGTGGGGGGAAGATGGGGATCACAG

AAATGGGTCCAGGA**GGGTGGG**AAGGACAGTGTGGGTGACTTTCCTATTGATAGGGAGACCCATGCCTGGG

GAGCCTCAGTCAAAGGGGTCCTGGGTCACTGCCTCCACTGTGAGACCCTCTGACTCTCCTGAGCACCCAC

CCCTGGGCTCCTCCTCAAGCTGGGGGTTCCTCTCCTGGCATCCTCAGCAAGATCACCTGACTCCAGCAGC

TGCCAACCCCCTGGGCAACGCCAAAGCCTCCTGGCCTCAAACGGGTTGGGCGACATTCACTTCCTCGCGC

TGTCCTCCCGACCTGCTCTGTCATTAGGAATTGCCTCGGCGGTGGCGGTCACACTAACAGCAACAGGAGG

AGATCGTGCTGCTGCTCAGCACCTCGGGGTGGCTCAGATCTGCTGGAGGCCCCAGGAGGGAAGGTGGATG

CTGCTCTCTCCGGTCCCAGCTAAAGTGAGTCTAGGGGCCTGGATGGGCGTGGGGAAGGAGCTTGGCAATG

TTGGAAGGTTGCCAACATTGGAAGGATGCAGGAGAATGAGAGAGAGAGAAATTGAGAGAGAGAGAGAAAG

AGATACCTGTGGGTGAGCAACTAGTCACAGCTAAAGCTATAGGACCTTGCACTGGGGCTCCAGGAGGGAA

TCCCAGGTGCCTGGAAGTATCATTCTGTCCCACTGGCTCCTGTGCTGAGGTGGGGATGGGGGGTGTTTTC

TCTGGGGTGGGGACACCTGCCCCCACACTCTGTTCCGGAGATGGGCTACTGCTGTGGGGGAGGCTCAGTA

CTGGGGCTTCCCAAGGTCAGCTCCCTTGGGCCAGCCATTGCCTTCCCCCTGCCTCCCAGGTGGCCAGAGG

GGCATCCTTGTTTAGGGTCACTCTAAACCTAAAGAGGAAGGAACTTTGGGTAGTCACTTTTCTAACAGTG

CTATTTCTTCTATTCCTTTGACCAAAGTCTGAAGACAAAGTCTTAAGCTCTGAGCCTCAGGTCCCTTATC

TGTAAAATGAAGATAATCCCTACGCCCCAGCCTTGTTGTCAGGATCAGAAGAGGAAGGGATGCAAAATGC

CAAGCATATAATTAGGATTCAATAAATGGTCGCTGTTGTAAATAATAATAATTATTTATTATTAGCTCAA

TACTGGTCCCACTTCACCTAGGAATACTTTCCAGCCCTCCTCTCCCCTCAGTTTCCTAAGGCACCAACAG

GGATGCTTTGGCACTTACTGCATTCTTCTTCAACTCGTTTGTGCTCAAACTAGAGTATGTTCCTTGCATT

TCTTCTGTTGCCCTCTCCCTCACCCCAGCATCCAAACAGAA

NPAS2 (adjacent to RPL31)

CCTTCAGCCTCCCACTCCCACAGCTGGCATTGGCCTCATGAGTGCCCTGAGAACGGTTCTGCTTGATGTG
CTGACTTGGGGGGCGCACCCCAGACAGGTGGGGCTCTGGGAAATGTGTGAATGAAGAAAGGAGCCAGGGC
TATGCAGCTCTGAGGGGTGGAGGGAACCCAGGCCTGCGTGTGGTAGGAGAGGAAGCTGGATCTGTGGGCC
TGAGAAGCATCAAGCCAGTGGCCTGGGAGAGTAGAGGCTCCGGTGTTGAGGAAGAACATTCCTGGATTGT
TACGTAATCTTTCACATCTGTGCACCTTCGCTGTCTCAAGGAGAGTAAACGCCCTAAGGGAGGAACTGCG
GATTACACTCCTTTGGGGCTGATTCTTCCAGGCCACTTGTCAGAGGAGCTTGTGTGGTTTGAATGGACAG
ATAAACACTCTCCTTTAGGATGTCATAGGCCACTGGGGTCCTGCAAGCAGGAGTGAACAAGGCCCGCTGT
GGTGTCGCTAACGGATCCCAGGCAGGCACTGGGAGGTGCGTGCGACCTAAGGCTTGAGAGGAACAGATGC
TGCTTCACTGAGCAGGTCTGGACAGCACTTCTCAAAGTGTGGTCTGAAGACCCTTGGGGTCCTTGAGGTC
AAGACATTTTTTCAAAATATAATTAACTCGTTATTTGCTCCTTTTAAAAGAGCTCTCCTTGTCTCATGAG
TGTACACGACATAATTGTTCTGACTTTTCCAGAAGTTACTTGACATCTGATGACTTCATTGCTCCGATGC
CCAATGAAATGAGTGCCTGTGCATTTAAACATTTCTCAGCCTTAACCCACATACACAGAAGTGCTTTGGA
GACCTCAGTCCATTTTGAGAGTGTAAAGGGGTGCCAGGACCAACAAGTTTGAGAACCACTGGGCTAAGAC
CAAAATGACAACAACCTGAAAGACTTTTAAGAAAGATGTAGAAGCAGACAGTAATTATCCATTCTTTTTA
ATTTTTTTTTTTTTTTTTGCAGGAAAAACTGCATAGAAAATCTAATGGATGAAGATGAGAAAGACAGAGC
CAAGAGGTAAGATGCAGCTGTCCCCCTGCTCAGCAGAGCTCTCTGGCCCCCGGGGGTCTGCCTGGCAGAA
TCTCGCTGGCTTTCACTCTGTGCAGGTGAGGCCACCAGCAAGATAGGCAGCTGTAGGACACACTCTCTGT
GACATATTGCAGTGCTCCATGAGAAACCTTCTAGAGAGCTCACTCAGCCCTGCCCCTCACTGTGAAATGA
CACATTGTCTAGGACTGGCTGAGTGGTCTGGGTTTTTGTCATGCTCCTGTGTACCCCTGTTGACACTGGT
CATCTTTCAGGGCTTCTGGTTACTGCTTTTCCAGGACAGACACTGCTTGACAAGTCAGGCTGACTGGAGC
CCAGCAGGCCTGGATTCAAGTCCTAACTCAGTTGCTGGGGCTCTGTCTGGCTTTGGATAATAATAATGAT
CATTGCCACACAGTGAGCATGCAGTCATCACCCAGCACTGTGCTAAGCACTTTTGTATACATAGATTAGC
TCAGTTTCCTCGGCTGTAGATTGGAGATGTCATAGACACCTTGCGCTGTGGTTGGGTCAACTAAAAATAC
TAACTTGGCAGCTGGCATGTGAGAGCCCTGATAACCTGTCTATGTTGATTCCTTCATTGGACATGTATTG
AATGCCTACTAGATATGCAGTGGAATCATAACAGTGACATTGAGAGGGTCAGGAACAGTTGAATGTCATC
AGTTTCACACGATTCACCCAAGTCGGCAGTTACTGTGCCATGCTCTGGGGAAAAAAACGTGAGCAGGAAG
CTCCTCAGTGGCTCACTGCAGGTCATTCGGGTGGAGGCCCAGCCACGAGCCTTGGCAGCAGGGGACATCC
TCTCTGGAGACACTCTTCATTGCAACCCCACTGGACCAGCCTCCTTGCTGTGATCTCACCCCTTTCTGCC
TCTGCCACTGCTTGTGTGTGGCAGGGCTCTCCGTCCTCTGT

IQSEC1 (adjacent to RPL32)

TGCAATCCTATATCTGCACACTTACTTCCTGGCTTCCCCTTAGACTGTAAACATCATGAGGGCAGATACT
TGCCCGGCACATGGAAGGTCCTCGGGAGAGGAAGAGGCACCATAGCTAGACCAGAAATTCTCCCTAGCCA
CGTGCAGCCCCCTTTGGGACAGGACTGGCACAAACGAGGACTGCTCTGCCATCCACCGTGCAGCTACTGG
CGTGGTGCGCCCTCCTGGAACCCCGTGGTATAGCTGTTATGATCCCTTTCACAGTAGCATAATCAAGACT
CCCAGGAGAGACGCGGCTTGCCAAGGTCTCATCGCCCTTCAGTGGCAGAGACAGCCTTGGAGTCGGCCGG
CCTCACTCCAGGGCCAGAGCCCCGAGAGCTCCGCGGCTCGCGTGTCTCAGTGCCGAAGGCAGAAAAGCCG
CCTTGGAAAGTAGTGTCATCGTAGGCTCCGCGCGGCGCGTGCATTTTAGCAACAGACTTCCAGGTTTCCA
GCGCGGGCCAGGAAGGGGCCGGCGCCCCGCGCCCCGCCGGGTGTGTGACTTTGCCGCGAGGGCGCAGCCG
AGCGGCCGCGCCTGATGCTGCTGTTGCCATGGCGACCAGCGCCTCGGCGATTGGTCCGGCGCGGCCGCAC
TACGTTGCTCCTTGGGCGCGGGGGGTGGGGGAGGCTCCGGCGCGCCCGCCCCTTCTCCGGATGCTGCGAG
AGCAAACCCGGGGCAGCCTTCTCTCGACCCT**CCCCTCC**TCATCCCCGCGCCCCGCACCCCGGATGGACGG
GAGGGAAAAACCCCAAGGTGGGCCCAGGTAGCAGCGAGGTGGACGCCCCCGCCCAGCCTGGCGACCACGG
TTACTCGACGTCCTCGCTCGCTCCTGGGCACGGTGTGAGCGCGCGCTACGTGCCAGGGCCCTCCACAGCC
CGCTGCTGGCGCCGCCCCCCGCTCCCGCCCCAGCCTCCCTGGAGGAGCCCGCCACCCCCGGGAATGTGAC
TCGCCCCGCGATTTAATTTTATTCCCCTCCACTTCTTGCCTGAGCCGCCTGCTCCTCTTGGAAACACGTT
GAGCCTCCCCGCTGGAGAGGGAGCCAGAACAGGGAAGAACGGATTCACACAGGATGGCTTGCAGAAGACG
CTATTTGTGAGTATATGTGGCAGGTTTCTGCCTGAAGCCAGAGGCCAGAGGGGCCCGGCTGAGCTGGGAG
CTGGAGTGAGGGGCATCGTTGGGGGAGGGGCAGGTGGATGTGCAGGTGACTTGCAGAGGGAGCTGGCCAC
CCAGCAGGGATGCCGGCGCTCGGCCCCTGCAGCGGAGGTGTTTGTTGGTGATGACGATGAGAAAAAGGCT
ATTAGTCAGCAGGTCTTCAGCAGGCTGTGAAATTCCCAACTGTGAGAGCAGGTATCCCCCCTCCCAGATC
CACATTCAGGGTGCCCAGACCGGCTCCTACTGTTTTGGCTTTGGTTTAGCCCATAGCCTGTTTAGGAAGA
GAATCTCTGCCCAGCACGCCCATGGCCAGCCCTTCAGCCATGCCTGATAGAAGGTCGCAGGCAGGCTGAG
GCAGTGGCCAGGATGCTGCGTCCTCCAAGGGCAGGTGAAGGTCCAGCCCACCGTCCCTAGCACGGAGGCA
TCAGTGTCCAGCTGTGTGCCCAGGTGTTCTGTGGGCTGGCAGCAGAGCGGCTGTGTGCTGGGCCTCACGC
CAGCAGCAGAGGCTGTGAAAGTCTGCACGGGGCGGTGACATCACGGGCCCGGGTATTTATGGAAACCAAA
GCCTGCGCTTCGGTTAGCCAGGCTTCCCAGGGGGCAGTGGGGTTCGAATGCCAGCTTCCCTCACAGCTCT
CTGGGCGCTGCACTGTGCTGTTCTAGGAGGAGCAACCTTTGCCCTCCTCTGGGACCCTCGGCTGCGGGGG
TTGAGGCCCAGCGTCAGGTCTCCACCTGCCTAAACTATTTGCCCAGGACCCACTCATTTGGGGCTGTGCA
GTGACCTCAGACCTTAGGAGGTTAGAGAGCATGGCTGAGTC

PRKAA1 (adjacent to RPL37)

CTAATCAGAGGAGTGGAATTGAGTAACAAACTCAAACATTTTATGAAAAATAAACAGACTTTATGCTATG
GTATGGTTCCATTTATGTTTATATAATAGACTAAGTATTACTCATTACAAGTACAATAAATATTTGTTGA
TCCGGTTGATGTAACCGTAAGCCTTGTTCTCTCTCAGTCCAGTGAAAGTATACATTTTACTGATAATCTA
AAAGTATTAATTCCTATACACCAAAAGATCTGCGTATCATCCCCCTCTTGGAATACACATTCACTTGCAC
ATATCCCCAGCCTGCTCCTCGCAACAGAGCTTGCTTTGGGGAGGAAATTTCAGGATCACCTCCAGAAGAA
AACACCGACAGCGCTCCTAGAGCCATCGTGGTGCCCTCTAGCAACTTACGGGCTCCAAGCGAAAAAAGAG
CTCGGGTTGGTGCGGGGGGGCGGGGAGCGGGAAACTCCATCACAGACGCAAGTGAAAACTCCTGCGGCTT
CTAGATTCCATCCGAGAAGGAAAGATGAAAATCAAGGTTAGAAAGCAGTGGCAGGGCGGTGTCCCCTGCC
TTGCTTTAATTCACGCGGCTCTCGTAACAAGCCACAGATGTGCATCACTCAAGCGGGCTTCCCAGTCCTC
ATCTCCCCTCTGGATGGTCTTCTGCGACCGCAGAGTGGTAGTTAGGGAAGATCGCAGCGGGCAGAAACTC
CGCCAGGGGAGAAAGGAGCCTGGGGGCGACGGTCCGCCTAATCGTTCCAGGAAGCCCCTCCGCGTGCCGG
TGGGCGGGCGCTCAGGCCCGAGGATGCGGGGCGAGGAGGCGGGTACTGGTGATTCTCCTGGCAGCCCCCG
CCCGCCCCGCCAGGGGGAGCGTCTGCGCCCGACGTAGGCTGCGGGGCCTGGGCTGGCGGCGCAGGGCGGT
GACTCGGCTCGGTGGCTGCCCTCCCGCGTC**CCCACCC**CCTC**CCCGCCC**GCCCCCGGCGCGGCCCTGCCCG
CCCCCTCCGCCCCCTCCCGCAGCGCCATGCGCAGACTCAGTTCCTGGAGAAAGATGGCGACAGCCGAGAA
GCAGAAACACGACGGGCGGGTGAAGATCGGCCACTACATTCTGGGTGACACGCTGGGGGTCGGCACCTTC
GGCAAAGTGAAGGGTGAGAACCCCGCGGGACTAGGCTTGGCCCCAGCTGAGGAGCCGCACGACTTCCGCT
TTCCGCACCTCTTCATCCTCCCCCGCCCGCTGCGTCCCTTCTTTCCAGGGCTGGGCAGCCCCTGTCCCGG
ATCCTGCGGGAGCTCTCCCCAGCCGCCGCGGGGCTGCGGCGCGGCCAGCACAGGCCGGGTGG**GGGTGGG**G
GTGGGGTAGGGGCCAGTCGCCATGGCGACGGCGCCGCGGCCTGAGGGGCTGGTAGGTACGAGGGGCTGGG
ATGCGAAGGTGCAAACTTTCCCAGGTGGGGCAGGCGGGCGGCGCGGCGCGCTCTGGGTGCTGGGAGGGGG
CGCCGGGCGCGATCCCCGGGGAGGCGGCATCTTGTGCGCCCGGGTTGTGCTCCCTTGGCTCAGGGCCAGC
CCATCGCTTCCTTGGCGGTCCTTGGCGTTGTCGGTATTTCCGTCCTCTGTAGGAGGCTGTCGATTTACCG
GCCCCTGCTGTTTCCCGAGTGCGAAATGTGTTTCCGCAGAAGTAAGGTTTCTTTGTGGGTAGCCGACATC
ATTTTTTTTAAAGACCATGTCGTCGGAAAGGATCACTGGTTTCCTTCGGAGCAGTTAATGAGTATAAACT
GGTTCAGTATTTGCGATGCATGGTTTACGTTCTGAATGAGACTGTGCCTTTAGTGTTTCCGAATTAACAG
GAGTTTCTCCGTGCGAAACTTCGTTGTTAGTTCAGATGTTGCCTCCAGATAGTATTTATTACTTTATTAG
TACAGTTCATTTCCACTTATTTTCAAAGGTTATTTGTATTCTAATAAACTCTTTAAGTGCACATCATTCG
AACTTTTGTTTTAAGAAAGGTGTATTGGGTGCATTGTACAG

Supplementary Figure S3. The occurrence of the ZTRE in the region 1 kb 5' and 1 kb 3' to the transcription start site for genes adjacent to ribosomal protein genes with transcripts increased in abundance by knockdown of ZNF658 in Caco-2 cells. Transcription start sites (taken as the 5' end each sequence specified) are highlighted in pink. Sequences matching the ZTRE segment C-A/C-C-T/A/G-C-C-C/T are highlighted in yellow. Sequences matching the corresponding complementary ZTRE segment A/G-G-G-C/T/A-G-G/T-G are highlighted in green. **Sequence in red and underlined matches a ZTRE segment that overlaps with another ZTRE segments**. Cyan highlighting between two of these segments indicates a space smaller that 30 bases; blue shading indicates a space between 30 and 50 bases.
