# Supplementary material for: The Zinc Finger Protein ZNF658 Regulates the Transcription of Genes Involved in Zinc Homeostasis and Affects Ribosome Biogenesis through the Zinc Transcriptional Regulatory Element
Source: Mol Cell Biol. 2015 Feb 18;35(6):977–87. doi: 10.1128/MCB.01298-14 (PMC4333095; doi:10.1128/MCB.01298-14)
Supplement: Supplemental material [file MCB.01298-14_zmb999100760so4.pdf]

POLR1A

CAGTATCAGATGGAGTGGTTTCTCTTTCTTGAGGGGAACTAAGGGAGAGTACATATAAAAACATATTGTA

AATTACATACCCCACTTATACCTCACAAACTTTTCGGATGGTTATTACCAACTCTAGTCTGCAGATGAGG

AAATCTGTCTATTATCATCCAATTAAGTGAATGGCAAACACTAAACCTAAAATTAGGCTTTTAGCAAGTT

TTCACTACAAATTACTGCCTCTCATATTCCCACAATGCCACTGTTTGTACTGAGTGAAAAAAAAATTACA

CAGATAGGTCATACATGTTCCAAGCTATAACTATCACAGATCAAAAGGGCATAGTCAGATCTTTTAGACT

TTTCTGCTTTTACGAAAACCTGATAACTTCTTTAGCCAAAGTCCCCACGGTGGCGACCAGAGACTGCCTT

TTATTTGGGAAGATCTTGTACGTTCTCCTCGCTCCTCCTGATTGGGTTAAAATGAGTAATGGCGGATAAT

TTTTAATTTGGGGTAGTGTTGCTAATAGCCACACTCACTGGAACGCAAGCTTCATGAGAGCAGGGTCCTA

GCTTTGCGGATGGTGTTAACTACAGAATATTGCAAGCAACCAATAAGTATTTCTCTAATGAATAAATGCG

TTGCGAATCCTATTTCAAAGGGCCACCTCCGCGTACCTGGTCTTAAGGGCACGCGACCGTTAAGGGCGCG

CATGAGCAAACCATTTCATCGTGCCTACTTCCACAGCCAGCTGGGAAACATAGGACTAAGGTGACTCCGC

GGTCTTCTTCGCGGATACCCTAAGCGTCTCTTACCTGCAGCTGCGTGCCTGTTCACACAAACCCGCCCGC

CGACCCGTCAGCGGCTGGCCAAGCCTGCTGCGGAGGCCCAGCCAGCGAACAGCAGATACAACCGCCATCT

TTGATTTCTCTGCAGAGCATGTTGGGAAAGCGTGCCTCGACCTTCAGGAAGGTGGGGCGCAGTGGGCGGG

GAGCAGGGAACAGCTCCGCTATTGGCTGAAGCGGAAGAAGACGAAAGCAATCATAAAATGGGAGGTTGCA

AGCTCATGGTTTGAAAGACTTCGTCACGGAAGCTAAAAGCTCTATACACCCGATTTGCCTCGGAGGAATT

TTCCTAAATGATTATTTTGATGTCTTATATATCTTTGATTGTTTTCAAAACAAAAGAGCGAGCAGAGAGT

CGTACAACTATTTGTTCCCCCCCCATGTAGAAGTGATCTCATCCACGTAAATGTCGTTCCTGCGACCGCT

TCCGCGCGCAAGCGCACGTTGAATCGCGTGGTGACTCCGGGCTTGAGGTTGAATTAAGAATAGTCAGGTG

GTGAGTGGAACGTCTCTTGGGGTGTCGGAATTCAAAACGGACCTGGAGGATGTTGATCTCCAAGAACATG

CCCTGGCGGCGGCTGCAGGGCATTTCCTTCGGGATGTATTCGGCTGAAGAGCTCAAGTAAGGAGTTGGTC

GGGTAGCGTGCCCAGCCTGGATCTTGAGGTCGGCGCCCTAAACTACGATTCCCAGAAGCGCTTTTGCCCA

GTCTATCCTCTTGAGTCCAGGCTTGTCTGGCGCTTGTCCTGCTGGGAGCCGTAGTTCTGGGAACGTTCCT

GGGCCGGCGTGGACTGCCTGGATAAGAGGCCGGTGGTGCTGGGGGAAAGAGTTTGCGCTGAGGTAGGGGA

ATATAAGGGTG**GGGAGGG**TCCTGGTCTGCAGCGTTATCATCTGCCAGTCTCCTCGTTAACTAACAAGCAT

TTGTGGAATTAACCATAGTGTACCTGACATGGTTAATTAGGCACGTAGTAAGTTTCCTCTTGTTTGTAAA

ATGATTGTCCTTCAAGTGTCAGTTCAGGCGTTAACTTCTCTTTGCTAGGACCCTGCTATATTCTCGGTCT

GGTTGTTAAATCAAGGGAACTCTCTTTAGTCTTCATTTTGATCAAGGTAGCATTTGACATTCTCCAATGC

ACTCTCTTGAAACTTTCCTTTCTTCCAGAACCTTATTTGTC

POLR1B

GAAAAATATCTAGTATTTGATAGCACAACAGGGTGACTGCAGTCAGCAATAATTTGTTGTACCTTCAAAA
ATAACTGAGGAAGTACAATTGGAATGTTCATGACAGAAATGATGAATGCTTGAGGTGATGGAAACCCCAT
CTACCCTCCTGTGATGATTACATCGTATGCCTATATGAAAACATCTCATATACCTCATAAATATGCTAAT
TATGTACCCATAAATTTTTTTTTAAATCCTCATGACCTTGGATTACACAATGATGTCTTAGATATGATAT
AAAAAGACAGGCAACAAAAGAAAAAAACTACACTTCAAAATTAAAACGTTTTGCTTCAAAGGACACCAGC
AAGAAAGTAAAAACCCACAGAATGGGAGAAAAATATTTGCAAATCATGGATCTGACAAGGACTAGTATAG
GCTATATAAAGAACTACAACTACTCAGTTGGCCGGGCGCGCGGTGGCTCATGCCTGTAATCCCAGCAT
TTAGGGAGGCAAGGCGAGAGGATCACTAGAGGCCGGAAGTTCAAGACCAGCCTGTGCAACAGACTCCCGG
CTCGAATTTTTTATAAAAAGTGATATAGGAATGAAAAGTACCAGCTGAACTAGGAAAGGGGAAGTTGACA
GTAAGGAGGGACTATCAGGGGCAGGAGGATACTGGGTAGTGGTGGTTTAATTCATTGTTTTAATTGTGGT
GATGGCTGCATGGTTGTATACATAGGTCAAAACTTAACGCGTACACTTTAAATGTGTGGTTTATTGTATG
CCAGTTATAACGCAATATAGCTGTTAAACACAAACACAATAAAGTTGAAAAACCAGCAGGCATTCCCTGA
TTAATCAGTATTCTGGGGAGGTAAGGGGCTCACAGGAACGCAAAAGAAATTCAGGCTGTAAATCCAGTTG
GACGCCCCGCTGCCCCATCCCCTCCATCCGGGACCGCCCGACTCCTCTCCCACCTCCTTCCCCAGGCCTC
CCCCAGGCCTCCAGTCCGGAAGATGCGACGAGGTTAGCGGGGCCCGACCACTCCTTGGCTTCCCAGGGGT
GAGCCTCGCGAGTTAGGAGTTGGGTAGAGAGTCAGCCCGGGGCCCGGCATCCGCTTTTTCGTTGAAGCAA
CGACTTTGGCCGGATGACTCCCCAGGGTCGGCATCAGCGTGGGACTGGGAACAGGTGAAGGGAAACAGAA
GAGAGCCTGAGAAGACCGCTCTCCTCCGATCCTAATTGACTGAGCCAATGAGAGCCAAAGAGGTCACATG
CTCAACTGGGCGGGGAGCGGGTTTCCACCTGCGGCATCTTTCGCGAGCGGGGAGATGAGTGGGGCGGAAT
ATGGGAGAAAGGGAGGGCCCGCCACGCTCTGGCTGGACACGGCCTTAATCGGCCCGTTCACTCGACGTTT
TTGGTTCTACGTTGACCCCGAGAAACCGAAACCGCAGGGCCTAGGGCGGGTGGAACGAGAGGGAACTACA
TTTCCCAGCAGGCTGCGGAAACGGGACTGCGGCCACTACTTCCGGCGTGTACCGAGAGACTGGCGTCCGG
CGTGTACCGAGAGACTGGCGTCCGGTGTGCAGGTGGCCACATGGATCCTGGCAGCCGGTGGCGGAACCTG
CCCAGCGGGCCTAGCCTAAAGCACTTGACTGACCCCTCTTATGGAATCCCGCGGGAACAGCAAAAGGCAG
CGTTGCAGGAGCTGACGCGGGCGCACGTGGAGTCCTTCAACTACGCTGTGCACGAGGGTCTCGGCCTCGC
GGTGCAGGTGAGCGCGGCGTCCGCCGGCGCCCTTGCCGCGGCGAGGCCAATCCCAGGGAGGTGGCTGGGA
GGTCACAGTATGACCCCAGATGCATGTGCTCCTTGATCCTGACAGGCTTGGTGGGTAAGCGGGAGAGCAC
AACATGTTAAACAGGACGCGGTACTGGCCTTCGTGAGACTTGGAGTCGAGGCGTCTTAAGAGATGTATTC
GTGATTCTGGAGTGAAAAGTGATTGTGAACCGTAGTTTGGA

POLR1C

GCTAGAGCCTGAGGTATCATCCATGTTCTCCATGTCAATCACAGCTGAGCCTCCGCCCTGTGAAGACAAT
GGCTCCTAGAGTGCAGGACTTTTCTGTTCCATCCATTAAGTCCTCTGCTCATCCCAGGGTTTAGGGCTTT
GTTCTTCCACTGCTGAGGATACGGCTTGGAATCACTACAGATCATGCCACTCCATTCCCCCTCATATGCT
CTCAGAACGCCTGGTTTCTAGCTTTGGCTATCTTTTCTAGCCTGGGAAACCTACTAGTCTTCCATTCATG
TCTTTCTCCTATGTTACCTATCAAAGACTAAAAGGCTGAAAAAGTGCCCATTTCCTTCTTTCCTAAGCCC
CAAACAAAACTATACACATAGTATGTTTTCAGCGAATGCCAGTGAATCCCACCCAACCCCACTCCATGCC
TTACCCCGACCAACCTCCTAACTTGTTCCAAGAATACCTTCTTCCTAATGCCTCGGGCGGGCCCTGAAAC
CCGAGATCCTCAGGCTGGAGTAGTAGTCTGGCCCTTTCAGGTCAATGTTAATCCCACTGACTTTAGTGTG
CACGAAGACCCAAAGAAGAGAGTTTTGTAAATGGAGCGGACTTCCAGGCCCAGAGGGGGAATCCCCAAGA
CACTTCTGGACATCGCCGGCCCTCCGGCTGCTGGCAGCTGGTGCCTTGGGGCCATTACCTGGATGTTTTC
TTCGAACCCTCCCCATTCCGGGCCAGCTCCATTTCGGGCGCCGCCCGCCGGCGCCGCTGTAGTTGCCATG
TCCCTTGAGGGCTCCCGTCGCTGAAACCCGCGCTAGCCCCGCGCGCGGAGTGGGCAAGATGTGGGCCTCC
GGAAGGTAGACGTCCAGGGTCGAGGAGAGGTGGGATCGGCCGGAACACAAAAAGGAGAAGCCCGGATGTT
TGGACAAAAGCAACTTCCGGAAGCTGGGGACGTGGACGGGGGCGGGGAAAAATCTCGCGATATTTAAGAT
TCCAGGAGGCGGTGCGTTGCCACGGAGACGGAGCAGGGCTACTAGGACGCACGCGCGAGATAGAACCTCT
AGTCTCGTGGAGAGATTGAAGATGGCGGCTTCTCAGGCGGTGGAGGAAATGCGGAGCCGCGTGGTTCTGG
GGGAGTTTGGGGTTCGCAATGTAAGCCTTGTGGCCTTGAGCTCGGGCGGGAGGAATGAGAGCGGAACAGG
GATGGGTCTTGGGATTGGCGTGGGGATAGCTGTGGGCTCACTGTCCCTTCGTGGACAAATTCGTCCCTTT
GCTCTAGGTCCATACTACTGACTTTCCCGGTAACTATTCCGGTTATGATGATGCCTGGGACCAGGACCGC
TTCGAGAAGGTAAGTGGGGCCGGAGTTGTCTGGGGAGGGTTATGAAGGCCAGACCTTGTGGTCTGAGCAG
CCTGTGTCTGTCTCAGAAGCTGCTCTTGGGGGTCGCTCCGTTTGTAAGTTTGTTGAGCAATGTGCTAGAC
GCTCCGTTTACAGGCCAAATTAGACACGGTTTCTACCCAGTTCTGCAAAGATGTTCAGTCTTGTTGAGGA
ATGGTGTGTATAAACAGTGAATCTAGTGTAATATGGTGAAGGTGGTTGATAGCGATGAAGACGGCAGGGT
TGGGGTGGGGAGCATTACGCTGAGACCAGGAAGAGGGGCGGAGAGGTACTTAACATTTCAAGAGGAGGGT
ACATCTCAATTGAGCCTTGAATGGTAAGTAAAAAAGAACAGGAGTTAAATTATGAAGGGTTTGTTTCAAG
TCAGTGAAGTTCAGGATATACAGGTTTTGTGTGTATTTAGTCTTTTGTATTGTTTCACTTAGCTTAAAAT
AAGGGTTAAAATTGCATTTAAATAGATTACTGAATGTTACCGTTTAGGCAACTGACCTTTGCGTGCAGCT
TATAAACATGATCTGTAAATCAGTGCTTGGGAAAATTTTACTTTTTTAACCTGTTGATACAAGGAATAAA
GTATGAACAATGAAAAAGGTTAATGCTTGAAAAAAAATTAT

POLR1D

GGGCTGTCCCCGCCGCCCGCCTGATTCCCCTGCGGAAGCCCCGCTGGCTAGGTGTCTGCGCGCACCCCCG
GCCGCCGCCAAGAGTGGCACAGCTGGGGAAGGAGTGAACATCCAGAGGAATCGCGGGGCGGGGTGGGGGG
AGGCCAGGAGAACAGGTGGCGCAGGGAAGCAGAAACGTAGTTGCGCTGGACAGCTGGAGTCGGGCAGGGT
CGGGGCCAGCGGCGAGGGGACAAGAAGGGCCGATTCGAGAGGAAGCGCACCTGAGGGAGCACAGCGAAGG
CCGGGACGCAGGGAGCTGGATGGAGAGCGAGCCTGGGCGGCGAAAGGTTGCCGAGGCGGGGGCTGGGCGC
CTGGGAACAAAGGTGAGGGCGGGTTCCGCAGGTGAGGAGGTTTGAGGAGCCACTGGGGAGAAAGGGAAAA
GGAAGTCTACAGCCGGAGAGGACGCGCTCTGGGGGTAGAAGGAATCCTAGGACGGAAAGGATAAGGTGGG
AAACAGTTTTCTCCCAAACTCTCACTCCCCCAAAAGAGACATTTCTTTCTTGCCCTATTTTGACCCAGTT
GAAAATGCAACTGCAAGCTCCCCGGTGAAACAAATACCGCCTTCAACCTTCGTATTCTGTGGTGACTCAT
CTCGCACGTGACCCCCCTCCCTCTTCAAGGTGTGTTGACCGACTACAGTTTAAGCCTGTCGTCCCACAAA
AGGCCCGGGAGGGCCGGGGTCCCAGGAAGGGCCATCCTGGGTCAACACCGCCCCAGTTGGGAAGTCGGGC
CGCTTTCGGACTTGCCACTGGGAAGGGAAGTGATTTGGGGGCGGCAGGTAAGGCCGCGCCCCTTCGCCAT
CACCTGGGGAAAGGTACCAGAGTTGAGCCGGGGCGCCCGCAGGCCTGTCTGGGCCTCGGGGTAAGGCGGC
GCTGGCCCCGCCCCCTGCCCCTCCCGCCGCGAGTGGCCTCGCGTGGTCGCCCAGGTGAGCCGCGGGGCCG
CGAGCCACCGCTCACCCCGCGTCGGGGCGGGGGCTGGGGGTGGAGCCTCATGCCCCGCCCCGCGGCTGGG
CCCTGCCGCGCCGCTGCGGCTCCTCCTCCCTCCTTCCGTCCTCCGCGCCTTCCGTCGGTCGGTCCTTGCT
TCCTGCTTCGCCTCCGCGCCTCGCGCTATGGGACAGAGCCCCCGATCCGCCAGCACCACCTGAGGATCCA
GAAACCGCCCCAGCGATGGAAGAGGATCAGGAGCTGGAGAGGTAACGGCCGAGGAGGAGGCGGGCGGAGC
GGGCCGCGCCCAAGGGGAGCGGGTGGCCGAGGGGCACGCTGCCTGGCCTGGCAGCCGGGGCCTGACTCGG
GGCGCAGAGAAGAAGCATGGAGAGAGAAGTGGGGAGGAGACATGAATGTGTTTCAGTTGAGAGGCTGAGA
GGTACACTAGCTATCAAGGAGGGAAGACAAGTTGGGAGACCAGAGTTAAGTATCCGAACTGCCCACCCCC
GTTACAACAGCAGTGTGTAAAAATCACGCCCCGGGCCTCTGCTCCTGAGGACGGTACTCGTGCCTCTGTC
CCTGTAACTACGGCTCACCTTCTTTCTATGTGCAGACTGCTCCTAAAGCCACTCTGTGCGCTGAGCTCTT
TTTCTCCCTGCTGCTTGACTCCTGAACCTTCCATTCTCCTAGTTTCCTTCTCTTGCAAAATGGAAGCCGT
AGTTGGGCCTGCACATTGATCATCTGTATCCTCTATTTTTATACTAGAGCAATCGGGATTTCTATGCCTT
TCAACCCTCTCTTCATCCTCTAGCAGGACCAGTCCTATTCTAATCCTATCCCTTGCTTGTGACTCTACCA
AAAAATATTTTTGCAAACACTTAAGTTTGTGTTTTTCTAAGAAAACTCCCCTTGTGGCCAGTGTGGATGG
TCAAATGGAGCAGGGAAAAACTGGAGGCAATAAATTAAATATTTATTGATAGAGTTTGTGGAGGCAGGAA
AATGAGAGGCGAATAATTCTGTGTAGCTGGAGTAGATTAAT

POLR1E

TGTTATTTAGGGAATGTCTGTAGGAAATACTATAGTCTTCAGGGTTGAAAGGGTATCAGGTTGACAACTT
ACTCTAAATGGTTCAGGGCAAAAACATTTCTTGCAGTGTTTCTTTACGTTTGAGATTGGGTAATTAGAAA
AGCGTGACAAGTGCTCAGGCAAAGGGGCATGCAGGCTTTAGGGTTATTCCTAGGATCCTGGGTGTTGGAG
GCGGGTGTTGAGGGTGTGTGGAAGGAAAAGAGTCTTGAAAGAAGGTGATTTGGAAGACAGAGGGGAGTCA
ACCGGAGTTCTTGCCCTTCAATAGTACGCATCTTTGGGCAAAGGGGGCCTCGGCCAGCGTCCATGCTTAA
AGGCCCTGCTTTCGCCTTCTTTCTGCTCTGAAAACTCCCCGTGCAGAAGAAGGGTTGAATCCGCAGTGAA
AAAGGGACATGTCCATCAGCTCGGACTCCCTCTTCTATTCCAGTCCAGAATCTCCTGCCCAAAGGCTCCG
GGGTTGGCTGGGCTTCTGTTCCATCACCTCAGTGTTGATTGGGCCACAGGTGTGCACGCCACAGCCCATG
GCGGGGAGCTGTTTGTCGGACTGTGGACAAAGTTTGTACCTAGAGCTGGATGTCCACGTGGGT**GGGTGGG**
AAGCCCTAGAGAGGGGCGGCTATTCGCCAGCGGCCACATTTAGTTGGGGCTCAAGCAGTTCCTGTCAGAG
AATTCTAAATTTGAACTTGGTCCTTCAGGTCTTGGGAAGATGAGTTTGTCACGGTAGGAGAGAGAGACGA
CACTCTCTTTAGAAGGTGCTGAGCCGGAAGAAGGCTCATAGCAAGCTTAGGCTGAAGCCTTTGGCGCCTC
GGGCTCTGGACCTACAAAACCCAGAGTGCACGCGAGCGCTGAGCCAATCAGGTGCTGCGCCTCGGCCAGT
CAAGCGGGTGTGTGGCACCTTGTGGGCGGAGACCACGCCTCTCTACGCGGGGGGCGGGGACACGCGGCTC
GCGCGCTGTGGGCGGTGCCCGGCGGGGGCCACGCCTTTTCCGGCCCGCAGCGCGGCCTGGGCTCCCGCGTG
TTTAAAAGTGCGCTTGTGGCTGCTGCTGTCTTAACTCCTGTGCTTGGCGGACAGACAGGCGAGATGGCGG
CGGAGGTGTTGCCGAGTGCGAGGTGGCAGTATTGTGGGGCGCCCGACGGGAGCCAGAGAGCTGTACTGGG
TAAAGACTGCGGAGCTGGAGCAGTGCTCCTCTAACCCTCTCCCCTTCCGTCTCGGTACCTCAGCTGTCCT
CTCGCCCTCCGTTGGGCTTCTCCCCAGCGGGGCCGGACCCCTCTAGTCTCCACCACTCCCCTGGGCTCT
GTCAGGGCTCCCCTGTCCGTCTTTCTGTCACTACCTCCCAGCCAGATTGGGACATCCCACTCACCCGCTC
CCCAGAGCTGTGTCCAGGACCCTGCTGACCCCCTCACCCACCAGGTCTCCCGCCTCCCCCATTTCCCTCT
CTTCACTGGGCAGGGGTTCCTTCTGTCACTTTAGGTATGTACCAGGCCTCTGCTGTCAGCCTCCTTCCGC
GAGACATTCCCTCCTGTCACAGCCCCTCCCCAGGGTTCTCCCACCTCCCTACCTCCTCTTCTCAGCTGGC
CCCGGATCTCCTCCAGTTCCCTTTGGGTCAGGACCCGAGCTTCCTTGCCATCCCCATTCTGACACT**CCCT**
CCCAGTGACAGTCTAGTCCCACCGTACATTGTGTGGTACATTGTGTGGCCTTCTGCTCTCATCTCATTCT
TGGGCTGCACTCTTACAGTCCAGTTCTCCAACGGGAAGCTACAGAGTCCAGGCAACATGCGCTTTACCTT
GTATGAGAACAAAGATTCCACCAACCCCAGGAAGAGGAATCAACGGATCCTGGTAAGTGAAGCAGTTGCC
AGCTGTCTCCACTCTGCAGGGCTCAGAAGGCCCCTGGGAGTGGGTGGATGTGGGA**GGGAGTG**AGGGGAGA
AAAAGGGGAGTAGTAGCAAATGGAGCTTTGAAGAACTCTGA

Supplementary Figure S4. The occurrence of the ZTRE in the region 1 kb 5’ and 1 kb 3’ to the transcription start site for RNA pol I subunit genes. Transcription start sites (taken as the 5’ end each sequence specified) are highlighted in pink. Sequences matching the ZTRE segment C-A/C-C-T/A/G-C-C-C/T are highlighted in yellow. Sequences matching the corresponding complementary ZTRE segment A/G-G-G-C/T/A-G-G/T-G are highlighted in green. **Sequence in red and underlined matches a ZTRE segment that overlaps with another ZTRE segments**. Cyan highlighting between two of these segments indicates a space smaller that 30 bases; blue shading indicates a space between 30 and 50 bases.
