# Supplementary material for: The Zinc Finger Protein ZNF658 Regulates the Transcription of Genes Involved in Zinc Homeostasis and Affects Ribosome Biogenesis through the Zinc Transcriptional Regulatory Element
Source: Mol Cell Biol. 2015 Feb 18;35(6):977–87. doi: 10.1128/MCB.01298-14 (PMC4333095; doi:10.1128/MCB.01298-14)
Supplement: Supplemental material [file MCB.01298-14_zmb999100760so5.pdf]

Supplementary Table S1. Sequences of siRNAs used to knockdown expression of ZNF658 in Caco-2 cells. The 4 siRNAs from Dharmacon were supplied and used as a mixture (Dharmacon Smartpool).

Analysis downstream of ZNF658 knockdown	siRNA sequence (supplier); numbered in accordance with NM_033160.5	Negative control siRNA
RT-qPCR or reporter gene measurement	siRNA1: $_{2143}$GGAGATCTTTCACCTACAATT$_{2163}$TT (Ambion)<br>siRNA2: $_{289}$CAGUGGGAUAUUGCAUUACUA$_{309}$TT (Qiagen)	Ambion Silencer Select Negative Control #1
RNA hybridisation to oligonucleotide microarray	$_{2541}$CCGUAUGAAUGUAACGUAU$_{2559}$TT (Ambion)<br>$_{958}$GCUCUGAUCUUAAUGAAUA$_{976}$ (Dharmacon)<br>$_{2814}$GGGAAGACUUUCUCCAAGA$_{2832}$ (Dharmacon)<br>$_{1544}$AGAGAAACCUUGUGAUAAC$_{1562}$ (Dharmacon)<br>$_{1000}$CCGCUGUUGAAUACAAUAA$_{1018}$ (Dharmacon)	Dhamracon negative siGENOME Non-Targeting siRNA#1
